# Supplementary material for: Cunninghamia lanceolata genome illuminates the evolutionary dynamics of gymnosperms
Source: Cell Rep. 2026 Jun 18;45(7):117566. doi: 10.1016/j.celrep.2026.117566 (PMC13415678; doi:10.1016/j.celrep.2026.117566)
Supplement: Document S1. Figures S1–S19, Tables S1–S10, S16–S22, S25, S27–S31, and Methods S1–S4 [file mmc1.pdf]

Cell Reports, Volume *45*

Supplemental information

Cunninghamia lanceolata genome illuminates the evolutionary dynamics of gymnosperms

Si-Zu Lin, Yu Chen, Chao Wu, Wei-Hong Sun, Zhen Li, Heng-Chi Chen, Jie-Yu Wang, Chang-Mian Ji, Shu-Bin Li, Zhi-Wen Wang, Wen-Chieh Tsai, Xiang-Qing Ma, Si-Ren Lan, Fei-Ping Zhang, Ya-Cong Xie, Lei Yao, Yan Zhang, Meng-Meng Lü, Jia-Jun Zhang, Di-Yang Zhang, Yi-Quan Ye, Xia Yu, Shan-Shan Xu, Zhi-Hui Ma, Guo-Chang Ding, Guang-Qiu Cao, Zong-Ming He, Peng-Fei Wu, Kai-Min Lin, Ai-Qin Liu, Yan-Qing Lin, Shao-Ning Ruan, Bao Liu, Shi-Jiang Cao, Li-Li Zhou, Ming Li, Peng Shuai, Xiao-Long Hou, Yi-Han Wu, Nuo Li, Sheng Xiong, Yang Hao, Zhuang Zhou, Xue-Die Liu, Dan-Dan Zuo, Jia Li, Pei Wang, Jian Zhang, Ding-Kun Liu, Gui-Zhen Chen, Jie Huang, Ming-Zhong Huang, Yuan-Yuan Li, Qin-Yao Zheng, Xue-Wei Zhao, Xiang Zhao, Wen-Ying Zhong, Xue-Wen Zhang, Zheng-Bao Xia, Ying Yu, Zhi-Wei Liu, Hong-Kun Zheng, Ray Ming, Yves Van de Peer, and Zhong-Jian Liu

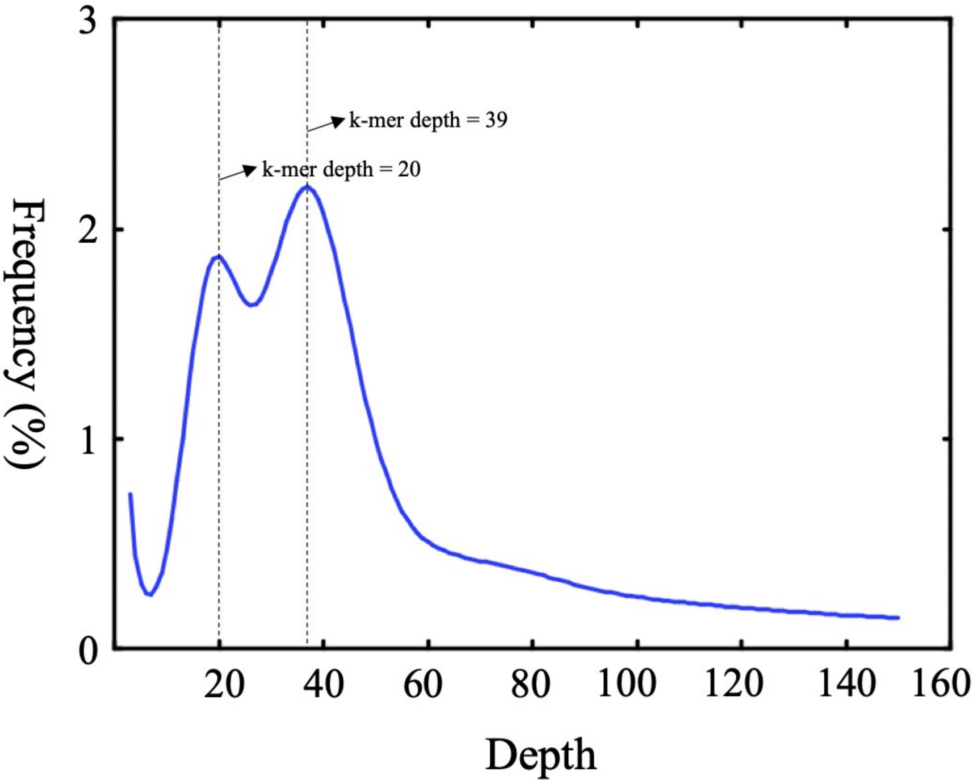

Figure S1. Genome size and heterozygosity of *C. lanceolata* estimation using K-mer distribution.

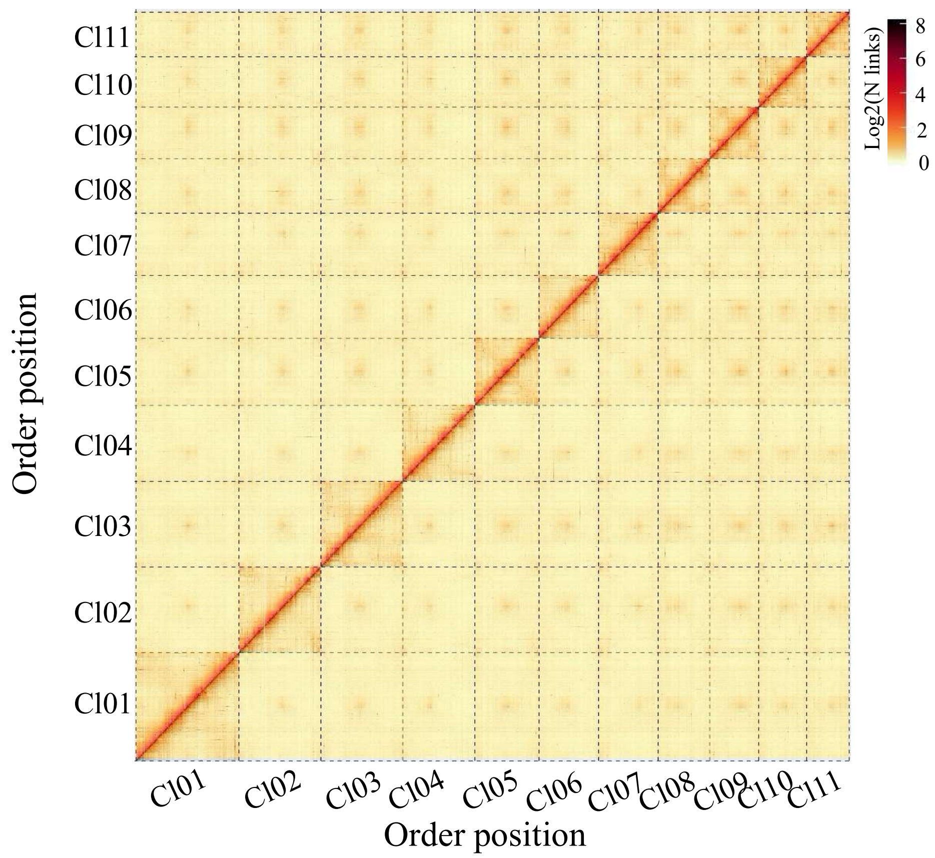

Figure S2. Hi-C interaction heatmap for *C. lanceolata* genome showing interactions among eleven chromosomes. Darker red pixels denote higher contact probabilities. Most interactions were observed within the chromosomes.

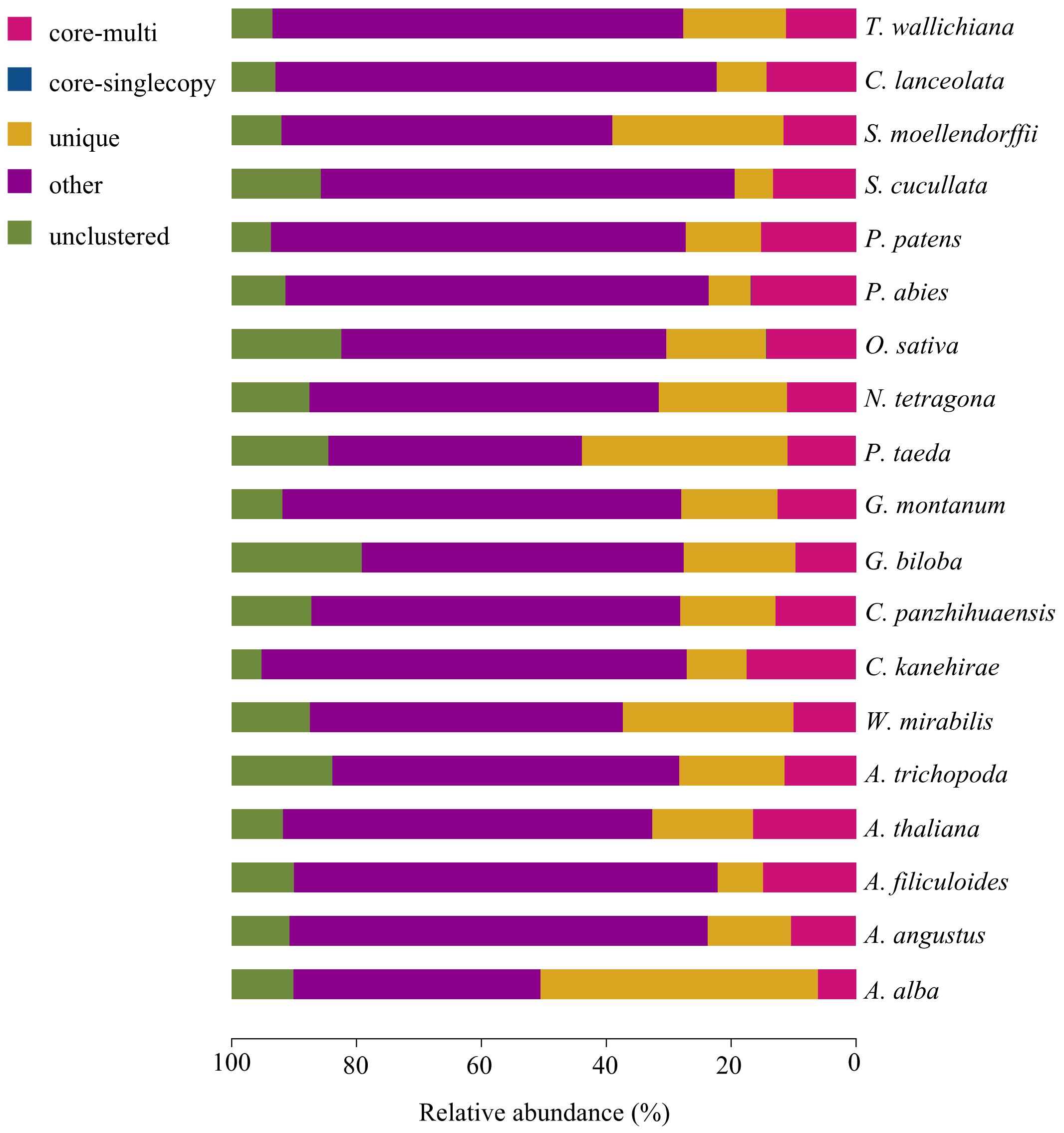

Figure S3. Orthologous genes in *C. lanceolata* and other species.

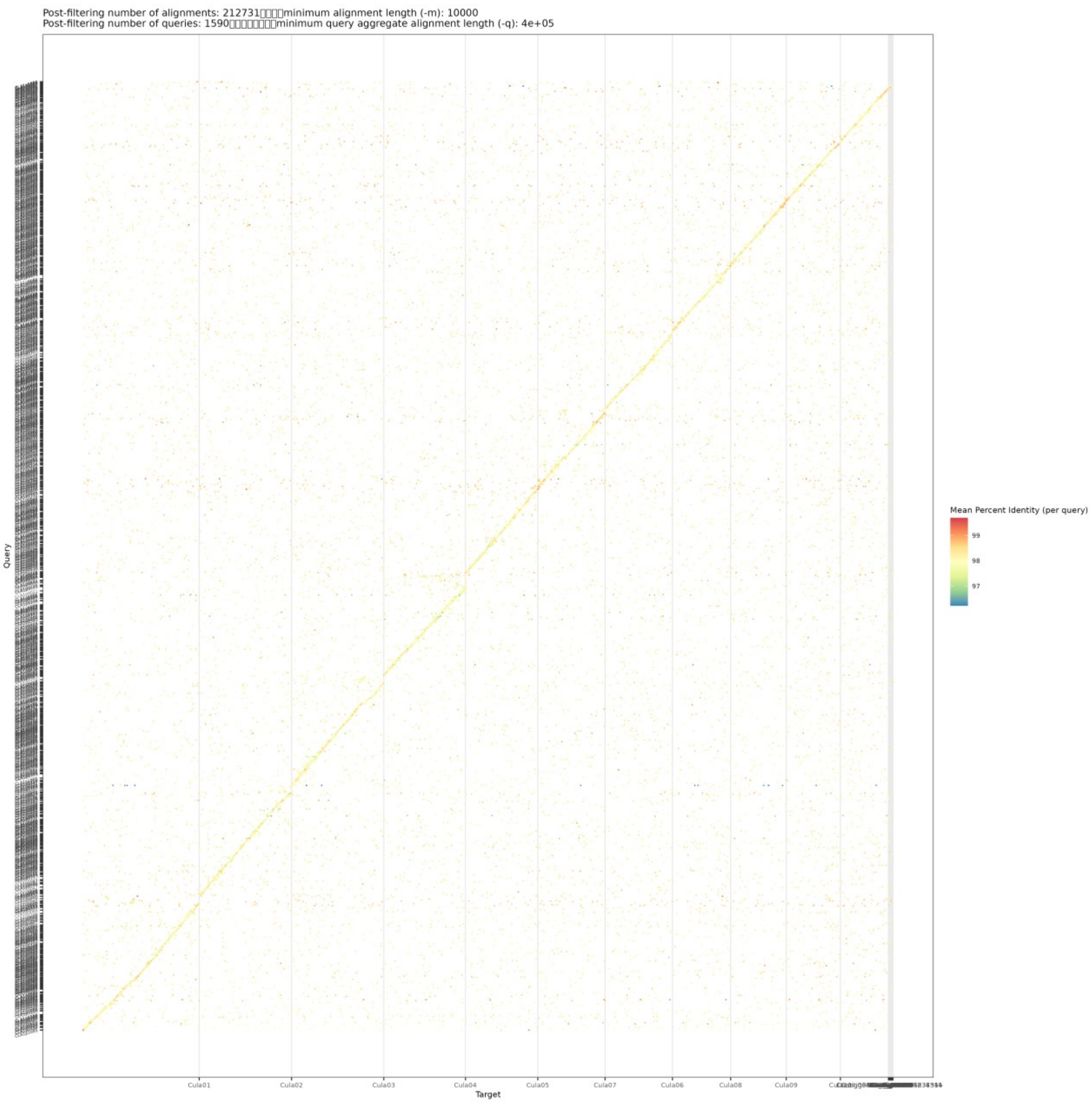

Figure S4. Visualization of the overage of the assembly map by Shirasawa et al. on chromosomal assembly in this paper.

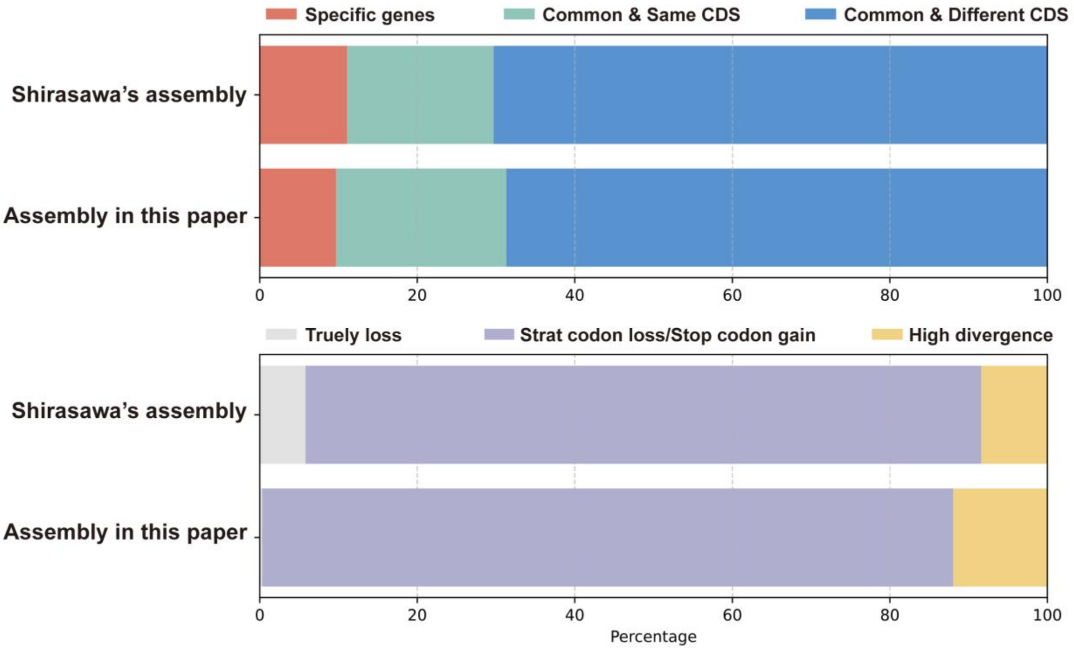

Figure S5. Comparison of annotation results between the two *C. lanceolata* genomes. Specific genes represents unique genes in the Shirasawa's assembly. Common genes represents the shared genes between the to genomes. Truly loss represents the genes completely lost in assembly in this paper. Star codon loss/Stop codon gain represents the mutations occurred in the CDS regions of genes in assembly in this paper, resulting in start codon deletion or premature stop codon acquisition with CDS sequences. High divergence represents orthologous genes exist in both assemblies but with substantial sequence divergence, with identity and coverage both lower than 80%.

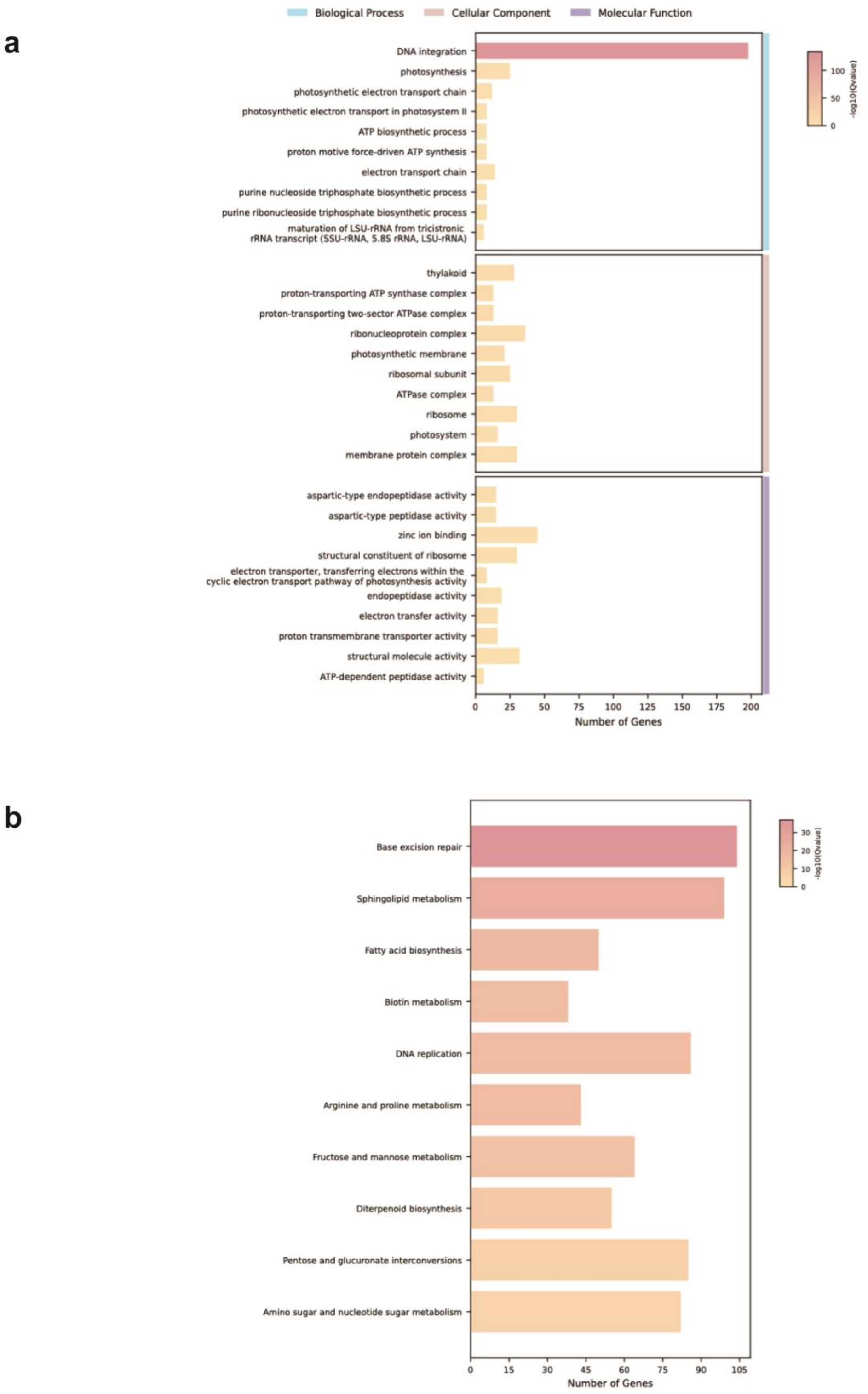

Figure S6. Enrichment analysis of specific genes. (a) GO enrichment. **(b)** KEGG enrichment.

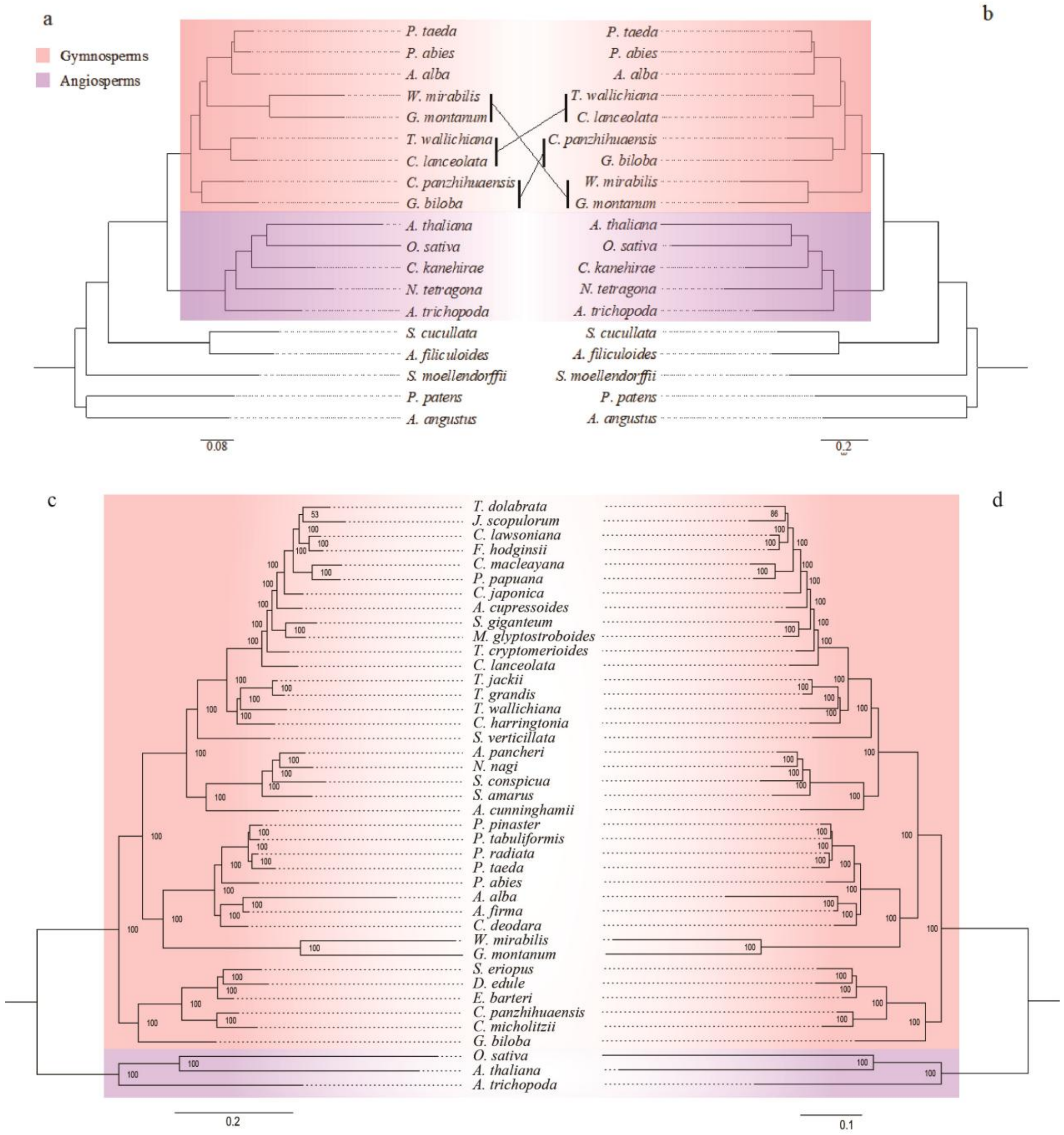

Figure S7. Phylogenetic tree constructed by different methods based on single copy-genes. **(a)** The concatenated tree based on amino acid, concatenated tree constructed by the first and two codons, and the Bayesian tree. **(b)** The concatenated tree based on nucleotides, ASTRAL tree, andASTRAL, tree based on constructed by the first and two codons. The topological structure ofconcatenated tree based on amino acid, concatenated tree based on constructed by the first and twocodons, and the Bayesian tree are the same, while the topological structure of concatenated treebased on nucleotides, ASTRAL, tree, and ASTRAl, tree based on constructed by the first and twocodons are same. **(c)** The concatenated tree based on amino acid sequences from genomes and transcriptomes of 32 species.**(d)** The concatenated tree based on nucleotide sequences from genomes and transcriptomes of 32 species.

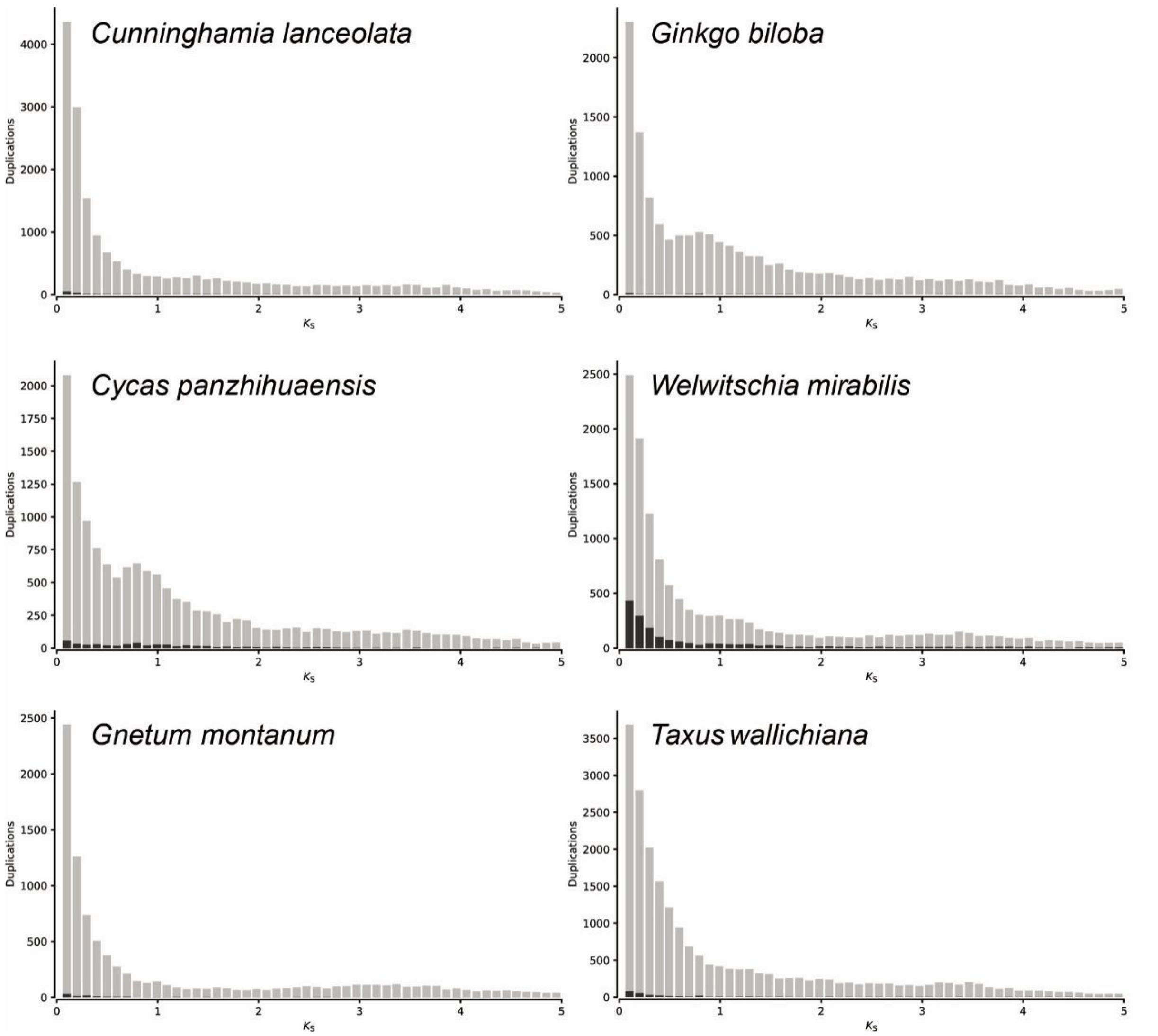

Figure S8. Distribution of synonymous substitutions per synonymous site (K_S) for paralogous gene pairs (gray bars) and syntenic retained paralogous pairs (black bars) in six gymnosperm species.

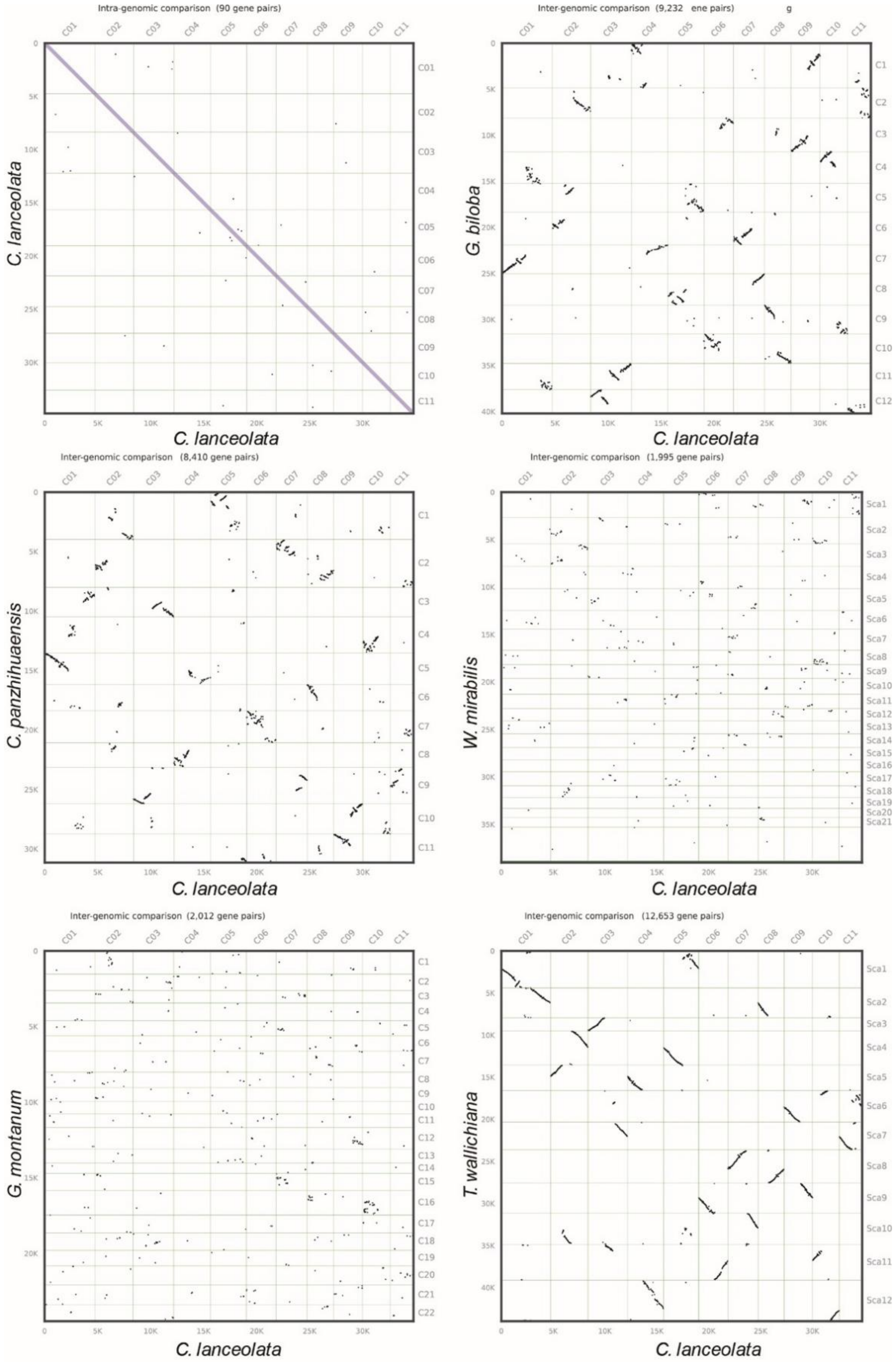

Figure S9. **Colinear dot plots for intra-genomic comparison within *C. lanceolata* genome and inter-genomic comparisons between *C. lanceolata* and each of five other gymnosperm genomes** (i.e. *G. biloba*, *C. panzhihuaensis*, *W. mirabilis*, *G. montanum*, and *T. wallichiana*)

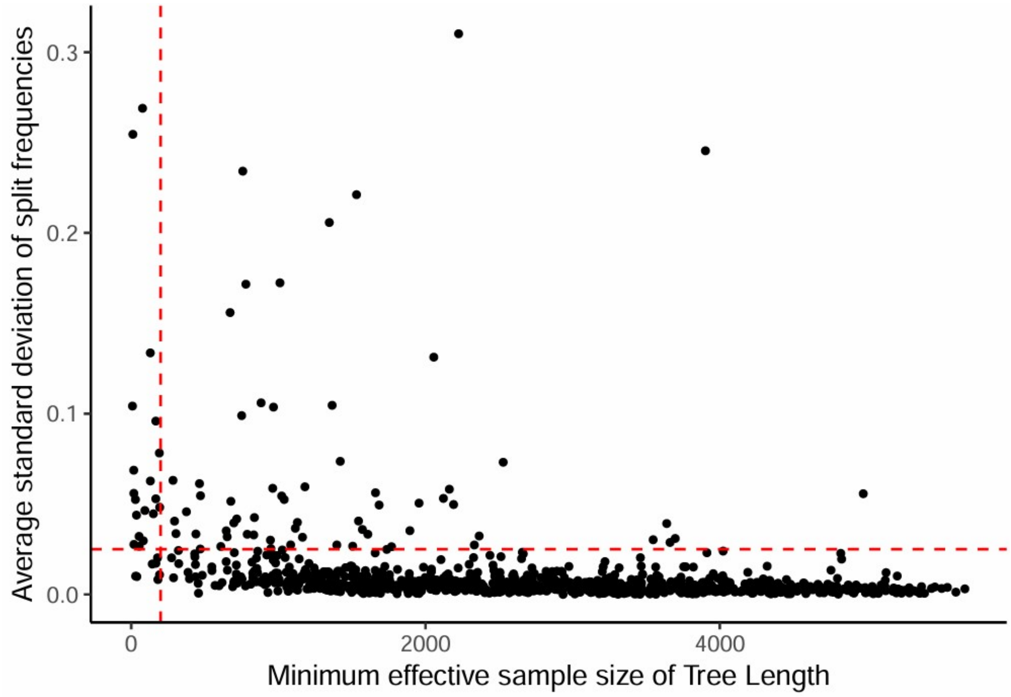

Figure S10. **Minimum effective sample size of Tree Length and the average standard deviation of split frequencies for the 1,000 randomly selected gene families.** The gene families with the minimum effective sample size of tree length > 200 (the red dashed line in vertical) and the average standard deviation of split frequencies < 0.025 (the red dashed line in horizontal) were selected for the WHALE analysis.

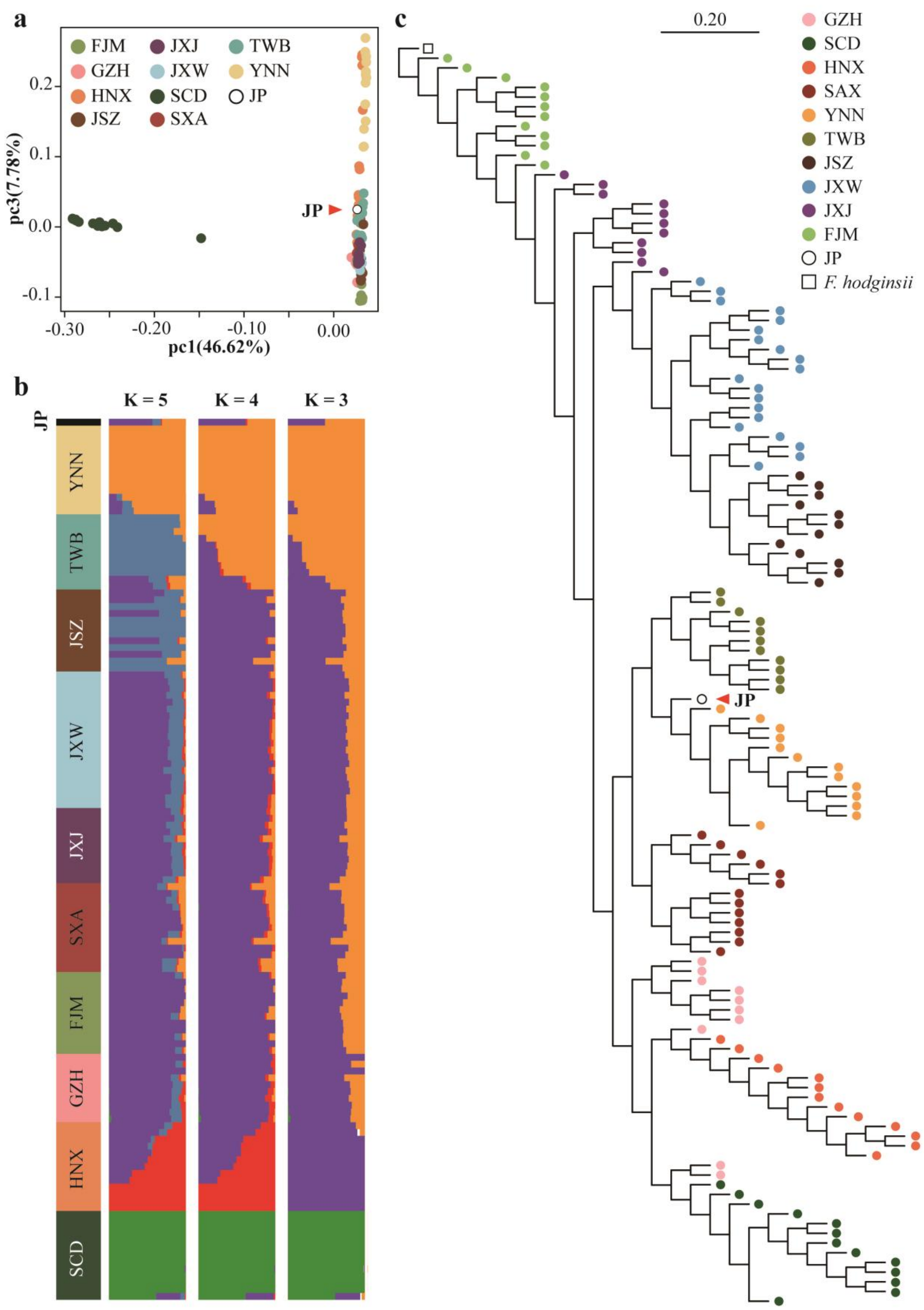

Figure S11. **Population evolutionary analysis of *C. lanceolata* with the inclusion of Japanese-grown *C. lanceolata* genome reported by Shirasawa et al..**

Consistent results from PCA **(a)** STRUCTURE analysis **(b)** and phylogenetic tree reconstruction **(c)** demonstrate that the genetic structure of Japanese-introduced *C. lanceolata* is highly similar to those of the TWB and YNN populations.

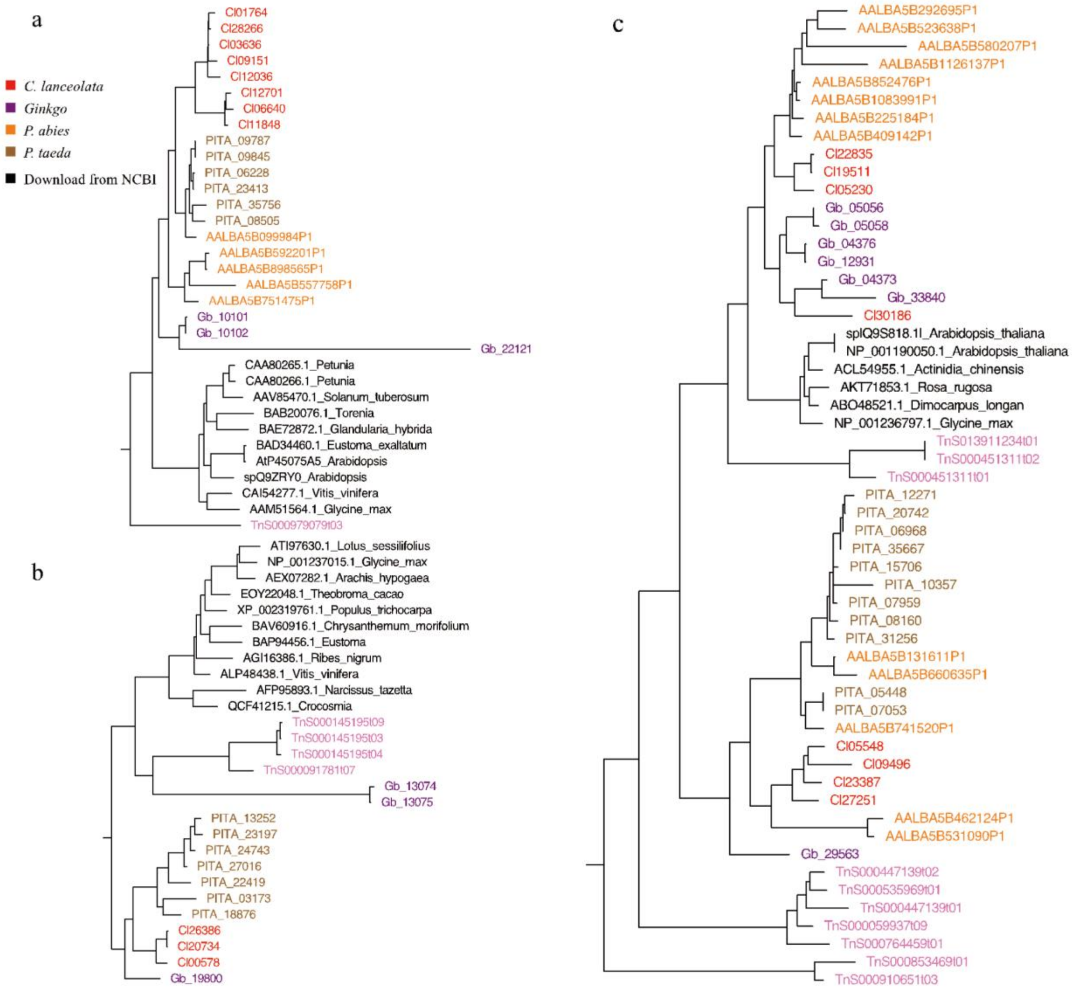

Figure S12. Phylogenetic tree of flavonoid 3',5'-hydroxylase (*F3′5′H*), flavonoid 3'-hydroxylase (*F3′H*), and flavanone-3-hydroxylase (*F3H*) from seed plants.(a) Flavonoid 3',5'-hydroxylase (*F3′5′H*). **(b)** Flavonoid 3'-hydroxylase (*F3′H*). **(c)** Flavanone-3-hydroxylase (*F3H*).

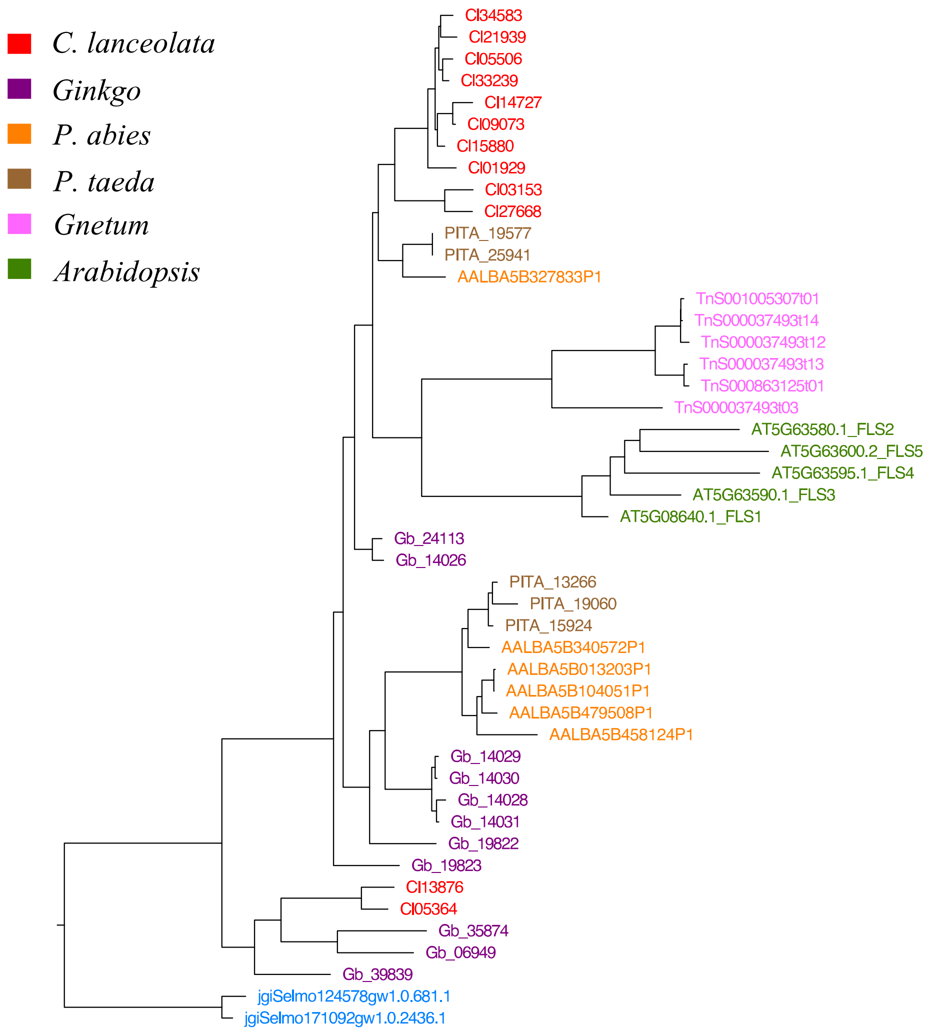

Figure S13. Phylogenetic relationships of flavonol synthase (*FLS*) gene. Two genes, *jgiSelmo124578gw1.0.681* and *jgiSelmo171092w1.0.2436.1*, from *Selaginella tamariscina* as outer group.

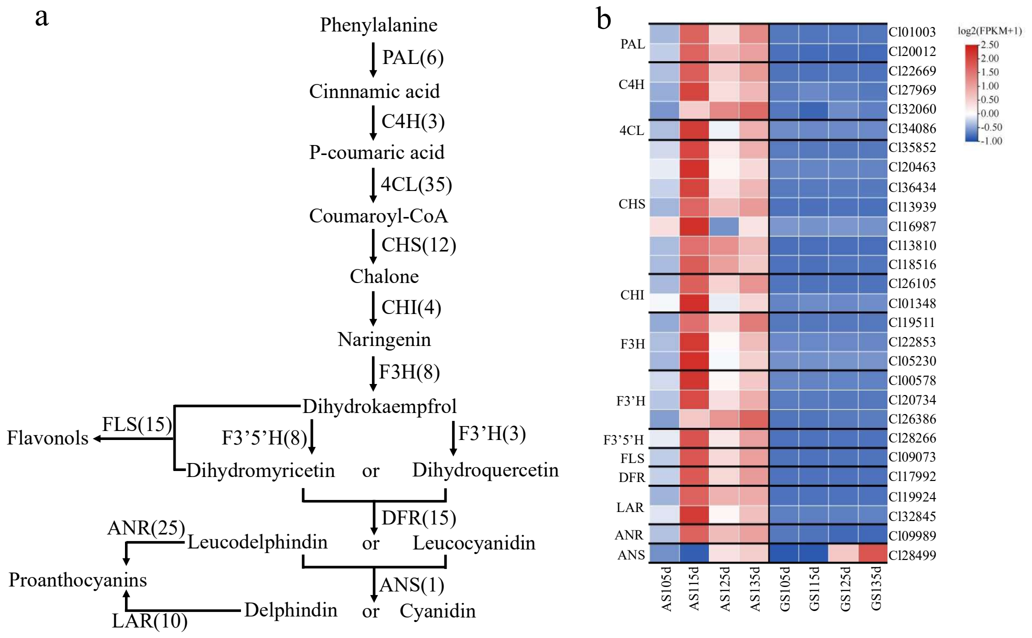

Figure S14. Expression of flavonoid synthesis related genes in astringent seeds (AS) and germinating seeds (GS) during different developmental stage. (a) Synthesis pathways of flavonoids. **(b)** The expression patterns of genes related to flavonoid synthesis in astringent seeds and germinated seeds at different stages. (see the full name of genes in Table S23)

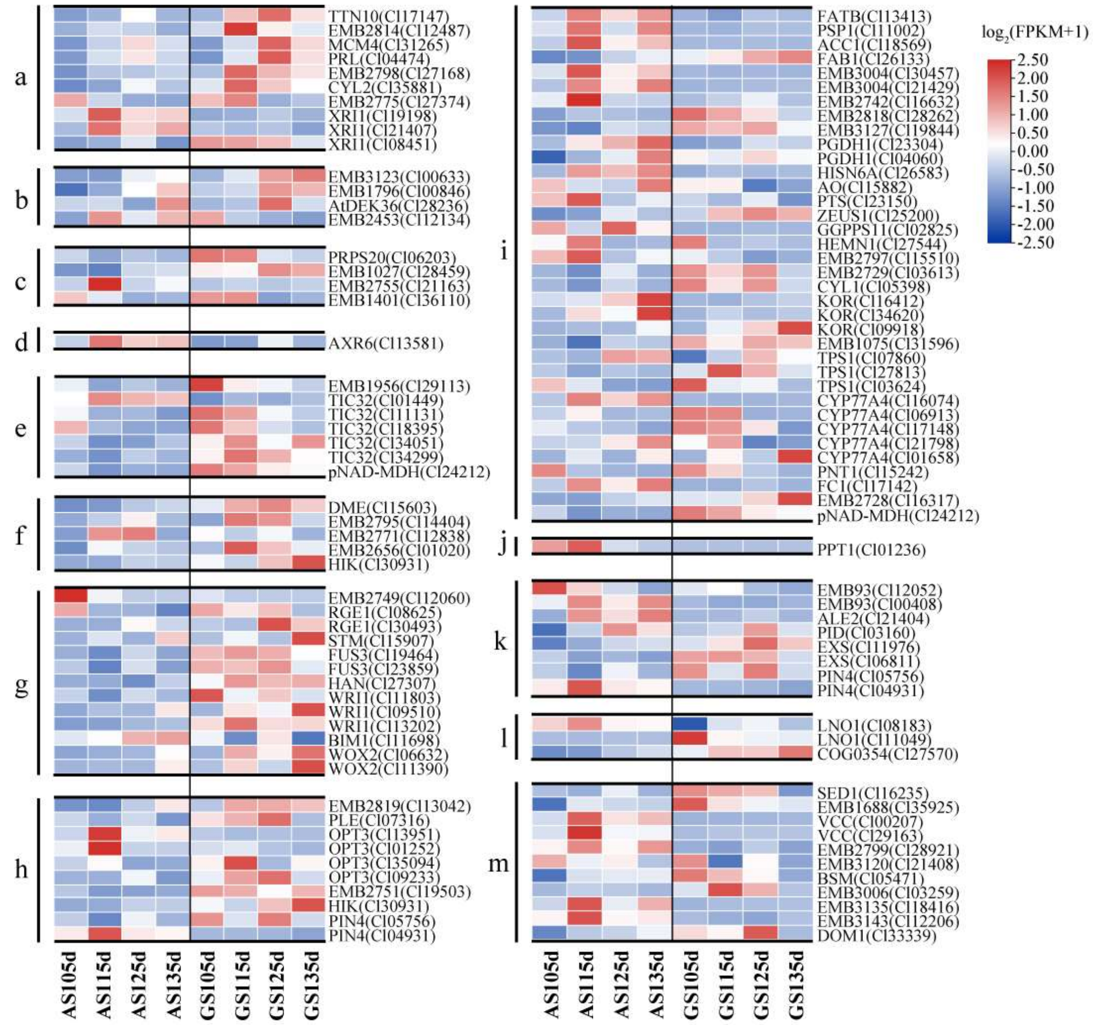

Figure S15. Expression pattern of 109 differential embryo-defective genes (*EMBs*) in astringent seeds (AS) and germinating seeds (GS) during four developmental stages. The letters on the left of the heat map represent the functional classification of the gene, the right side is the gene name, and the *C. lanceolata* gene ID is in parentheses. (**a**) DNA synthesis/repair.(**b**) DNA synthesis/modification. **(c)** Protein synthesis. (**d**) Protein degradation.(**e**) Protein modification/transport. **(f)** Chromosome dynamics. **(g)** Transcriptional regulation.**(h)** Cell structure. **(i)** metabolism. (**j**) Energy electron. (**k**) Signaling and regulatory pathways. (**l**) other (miscellaneous). (**m**) Uncertain/unknown.

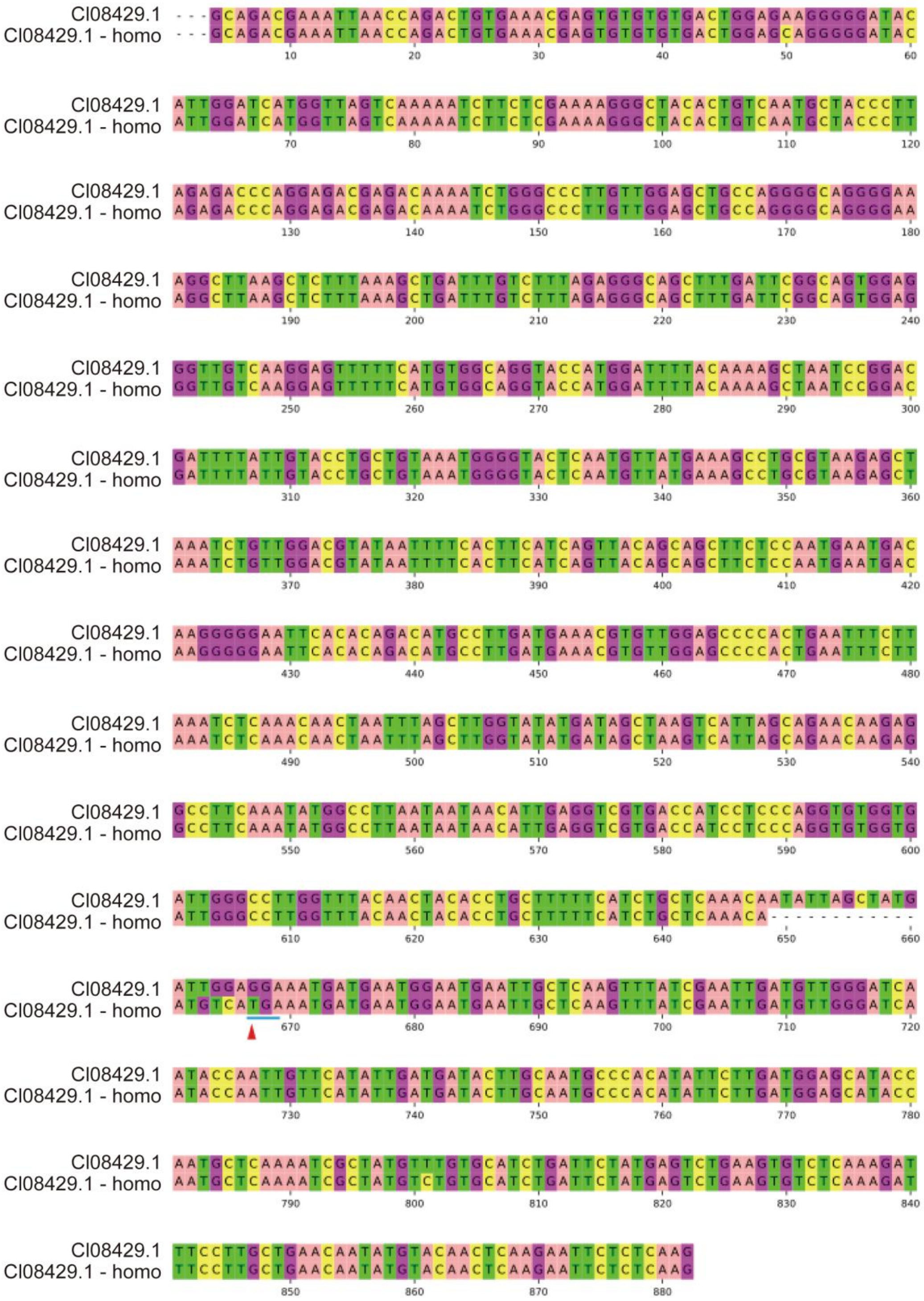

Figure S16. CDS alignment between Cl08429.1 and its homologous gene Cl08429.1-homo in the Shirasawa et al. assembly.

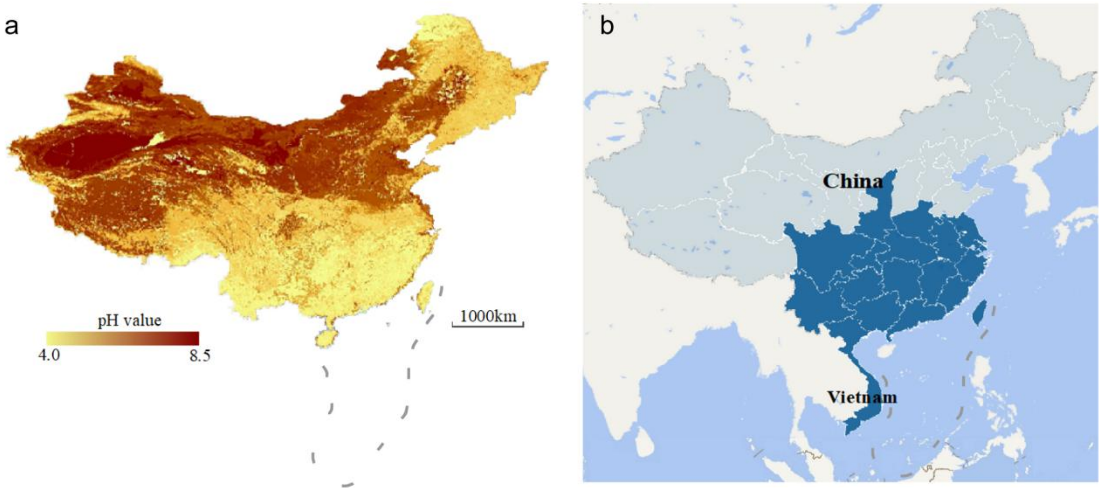

Figure S17. The distribution of *C. lanceolata* coincides with the distribution of acid red soil. (**a**) China's acid soil distribution map. Data sourced from the National Soil Information Service Platform of China (http://www.soilinfo.cn) (**b**) *C. lanceolata* distribution (dark blue) map.

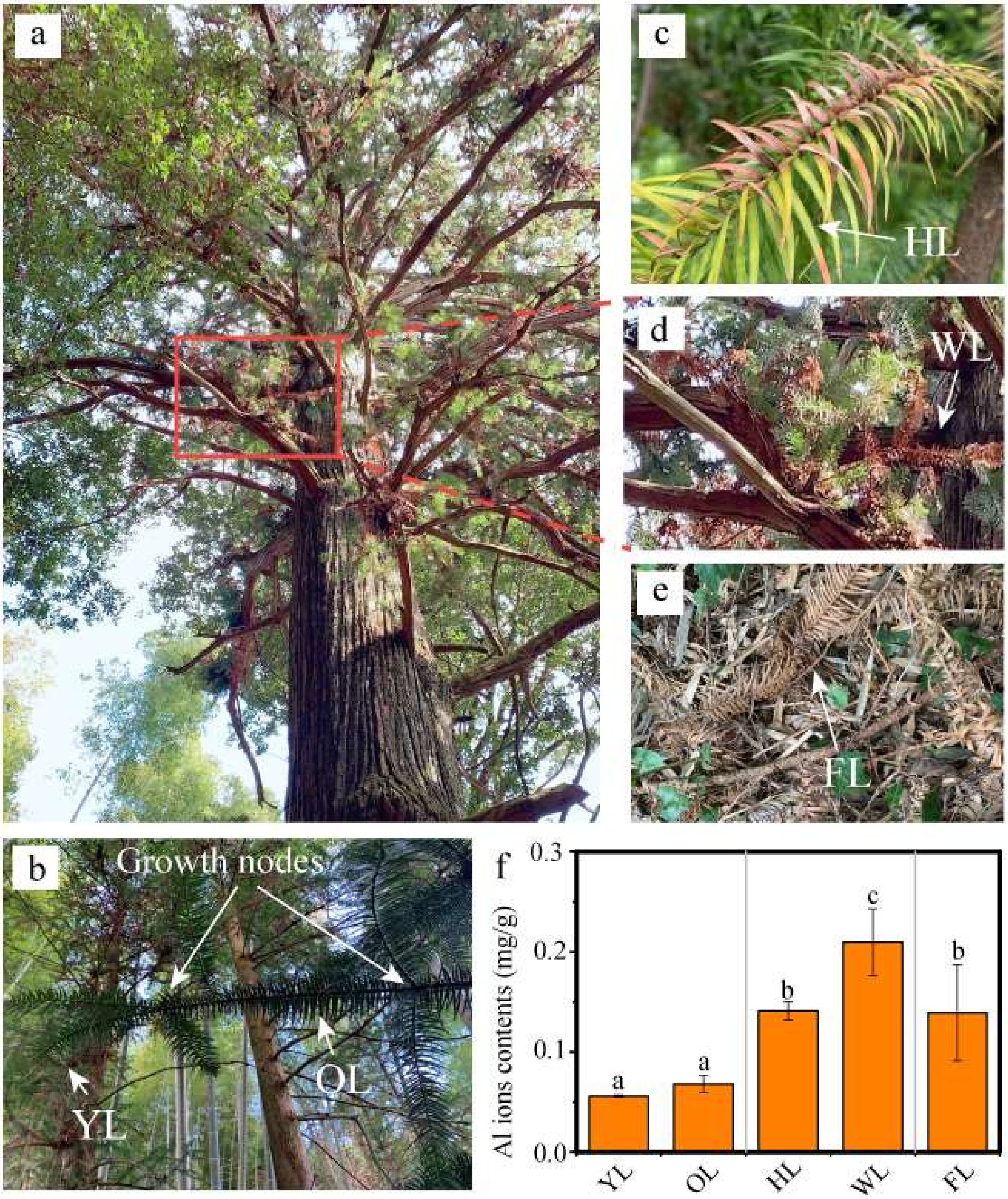

Figure S18. *C. lanceolata* leaves in different growth periods and their Al ions contents. **(a)** A mature individual of *C. lanceolata*. Living and withered leaves could be observed in this individual. The details of the leaves at different stages: **(b)** The young leaves (YL) and the old leaves (OL). **(c)** The half-withered leaves (HL). **(d)** Persistent withered leaves (WL). **(e)**The fallen withered leaves (FL). Withered leaves fall off with the branches only when self-pruning occurs. **(f)** The aluminum ion content in different growth stages of leaves. There is no significant difference at p = 0.05 level between groups labeled with the same letter.

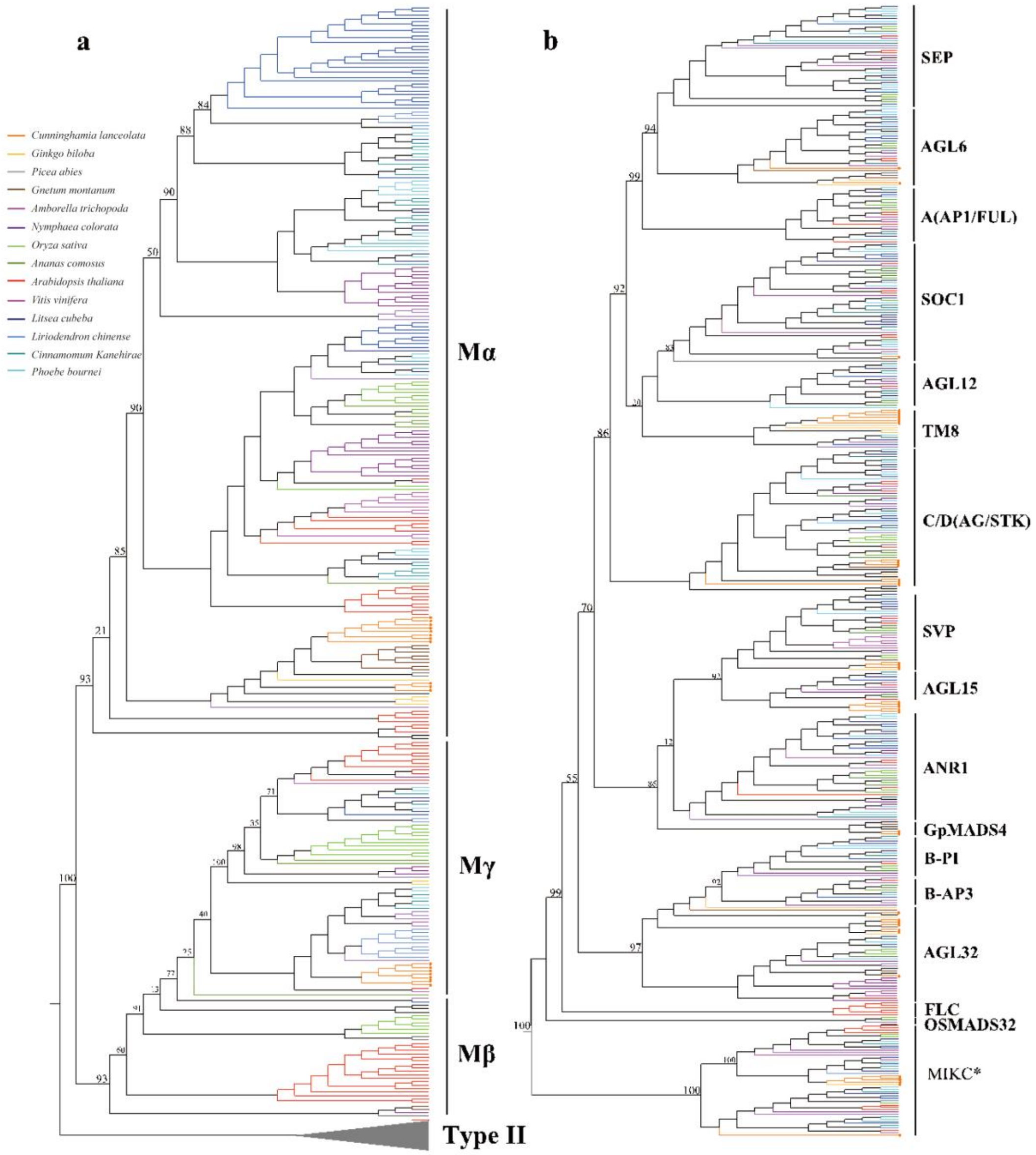

Figure S19. Phylogenetic tree of MADS-box genes from gymnosperms and angiosperms. **(a)** Phylogenetic tree of MADS-box Type I genes. **(b)** Phylogenetic tree of MADS-box Type II genes.

Table S1. The statistics of the quality on the PacBio sequencing data of *C. lanceolata* genome.

Reads type	Reads num	Total bases (bp)	Reads N50 (bp)	Mean length (bp)	Longest read (bp)
Subreads	89,507,038	1,113,159,857,423	20,648	12,437	129,985
ZMWreads	66,589,949	959,970,038,241	23,042	14,416	129,985

Reads Type: Subreads is the data set produced by Subreads, and ZMWreads is the longest subreads data set in the ZMW hole.

Table S2. The statistics of the Illumina sequencing data volume, sequencing depth and quality inspection of each library of *C. lanceolata* genome.

Library	Data (Gb)	Depth (X)	Q20 (%)	Q30 (%)
270_1 bp	67.40	6.47	94.87	85.19
270_2 bp	61.53	5.91	94.23	86.42
270_3 bp	65.17	6.25	94.67	87.25
270_4 bp	58.48	5.61	94.94	87.86
270_5 bp	63.25	6.07	94.34	86.70
270_6 bp	66.94	6.42	94.42	86.88
270_7 bp	70.19	6.74	94.50	87.06
270_8 bp	63.46	6.09	94.31	86.47
Total	516.42	49.56	--	--

Library: the sequencing library of the survey graph; Data (Gb): the amount of sequencing data of the corresponding sequencing library; Depth (X): the depth of sequencing; Q20 (%): the percentage of bases with a sequencing quality value of 20 or more; Q30 (%): The proportion of bases whose sequencing quality value is above 30.

Table S3. Assembly statistics of the *C. lanceolata* genome.

Type	Number/length/percentage
Contig number	28,364
Contig length (bp)	11,242,038,337
Contig N50 (bp)	2,155,103
Contig N90 (bp)	232,277
Contig max (bp)	22,388,200
GC content (%)	36.95

Table S4. The length of chromosome by Hi-C assemble of the *C. lanceolata* genome.

Chromosome	Cluster Num	Cluster Len (bp)	Order Num	Order Len (bp)
Chr01	3,692	1,550,680,665	3,251	1,488,319,881
Chr02	2,701	1,263,832,510	2,267	1,184,309,377
Chr03	2,640	1,234,995,222	2,238	1,182,746,149
Chr04	2,760	1,178,736,379	2,063	1,047,693,580
Chr05	1,913	960,425,685	1,611	927,725,105
Chr06	1,901	931,596,408	1,552	862,932,688
Chr07	1,804	897,221,591	1,527	863,305,216
Chr08	1,365	778,813,723	1,139	747,012,144
Chr09	1,232	749,408,730	988	712,708,477
Chr10	2,070	710,732,703	1,914	694,515,374
Chr11	1,682	637,087,857	1,491	616,297,228
Total<br>(Ratio %)	23,760<br>(83.03)	10,893,531,473<br>(96.9)	20,041<br>(84.35)	10,327,565,219<br>(94.8)

Cluster Len (bp): The length of the sequence located on the chromosome. Order Len (bp): In the sequence located on the chromosome, the length of the sequence can be determined in order and direction.

Table S5. The statistic result of Hi-C assembled of the genome of *C. lanceolata*.

Type	Contig	Scaffold
	**Size (bp)**	**Size (bp)**
N90	227,972	616,446,228
N50	2,097,655	927,886,105
Longest	21,974,839	1,488,644,881
Total length	11,242,038,337	11,244.041,337

Table S6. Illumina sequence alignment statistics of the genome of *C. lanceolata*.

Type	Number/percentage
Total reads	1,882,235,817
Mapped reads	1,864,713,766
Mapped (%)	99.07
Properly mapped reads	1,737,960,488
Properly mapped (%)	92.83

Total reads: reads statistics of filtered data; Mapped Reads: Reads statistics of matched genomes; Properly mapped reads: Reads statistics of mapped genomes and paired.

Table S7. The prediction of gene numbers of the *C. lanceolata* genome.

Method	Software	Species	Gene number
Ab initio	Genscan		68,124
	GlimmerHMM		190,423
	GeneID		179,448
	SNAP		118,942
Homology-based	GeMoMa	*Arabidopsis thaliana*	25,405
		Ginkgo biloba	38,598
		Gnetum montanum	29,888
		Picea abies	32,849
		Populus trichocarpa	30,476
		Pinus taeda	53,107
RNAseq	TransDecoder		115,300
	GeneMarkS-T		58,760
	PASA		55,025
Integration	EVM		37,225

Table S8. The statistics results of function annotation in genome of *C. lanceolata*.

Annotation database	Annotated number	Percentage (%)
GO Annotation	18,857	50.66
KEGG Annotation	12,874	34.58
KOG Annotation	20,771	55.8
Pfam Annotation	29,051	78.04
Swissprot Annotation	26,239	70.49
TrEMBL Annotation	34,202	91.88
Nr Annotation	34,392	92.39
All Annotation	34,559	92.84

Table S9. Statistics on the annotation of non-coding RNA of the *C. lanceolata* genome.

RNA classification	Number	Family
miRNA	50	13
rRNA	2,930	4
tRNA	3,955	24
snRNA	284	8
snoRNA	208	2

Table S10. Statistic result of clustered gene families of 19 species.

Species	Genes	Unclustered genes	Clustered genes	Familys	Unique families	Unique families genes	Common families	Common families genes	Average genes per family
A. alba	50,466	4,996	45,470	11,628	2,556	22,384	876	3,119	3.91
A. angustus	14,629	1,358	13,271	8,461	428	1,953	876	1,529	1.568
A. filiculoides	20,203	2,016	18,187	9,615	473	1,478	876	3,015	1.892
A. thaliana	27,416	2,244	25,172	10,084	983	4,422	876	4,523	2.496
A. trichopoda	26,846	4,326	22,520	10,929	986	4,546	876	3,075	2.061
W. mirabilis	39,019	4,877	34,142	10,458	1,519	10,647	876	3,921	3.265
C. kanehirae	26,531	1,273	25,258	10,012	571	2,551	876	4,660	2.523
C. panzhihuaensis	32,353	4,114	28,239	11,851	1,083	4,940	876	4,182	2.383
G. biloba	41,309	8,606	32,703	12,991	1,646	7,400	876	4,008	2.517
G. montanum	27,491	2,223	25,268	10,999	984	4,245	876	3,464	2.297
P. taeda	36,732	5,668	31,064	6,380	1,995	12,092	876	4,051	4.869
N. tetragona	31,589	3,930	27,659	10,834	1,100	6,484	876	3,500	2.553
O. sativa	27,694	4,856	22,838	9,883	1,188	4,445	876	3,986	2.311
P. abies	26,437	2,273	24,164	9,880	545	1,788	876	4,466	2.446
P. patens	20,328	1,273	19,055	9,155	866	2,455	876	3,097	2.081
S. cucullata	19,779	2,821	16,958	9,634	387	1,205	876	2,641	1.76
S. moellendorffii	22,285	1,770	20,515	9,721	1,550	6,100	876	2,594	2.11
C. lanceolata	37,225	2,605	34,620	13,266	797	2,982	876	5,337	2.61
T. wallichiana	44,035	2,882	41,153	14,364	1,761	7,231	876	4,959	2.865

Table S16. Primary genome assembly statistics of two assembly version.

	Assembly in this paper	Shirasawa's assembly
Sequencing / assembly method	PacBio 20-Kb single-molecule real-time sequencing; Illumina paired-end sequencing; Hi-C sequencing	Long-insert libraries sequencing (PacBio HiFi sequencing); Short-insert libraries sequencing
Assembly level	Chromosome	Contig
No. of Chromosome	11	--
GC content (%)	36.95	36.8
No. of contigs	28,364	2,472
Total contig size (bp)	11,242,038,337	11,548,046,079
Contig N50 (bp)	2,155,103	11,743,989
Assembled genome BUSCO score (%)	93.64	89.10
No. of protein-coding genes	37,225	--
Assembled protein BUSCO score (%)	89.60	--

Table S17. The coverage of the assembly map by Shirasawa *et al.* on chromosomal assembly in this paper.

	Chromosome length (Lin et al.)	Mapped length (Shirasawa et al.)	Coverage (%)
Chr 01	1,488,644,881	1,404,697,105	94.36
Chr 02	1,184,535,977	1,106,996,180	93.45
Chr 03	1,182,969,849	1,120,323,086	94.70
Chr 04	1,047,899,780	962,253,861	91.83
Chr 05	927,886,105	880,128,580	94.85
Chr 06	863,087,788	803,707,019	93.12
Chr 07	863,457,816	815,136,686	94.40
Chr 08	747,125,944	704,957,323	94.36
Chr 09	712,807,177	679,238,847	95.29
Chr 10	694,706,674	656,190,478	94.46
Chr 11	616,446,228	586,853,238	95.20

Table S18. Statistics of *C. lanceolata* gene structure information.

Gene type	Number/length
Gene number	37,225
Gene length (bp)	1,112,864,908
Average gene length (bp)	29,895.63
Exon number	164,956
Exon length (bp)	43,643,130
Average exon length (bp)	264.57
Intron number	164,955
Intron length (bp)	1,069,221,778
Average intron length (bp)	6,482

Table S19. Statistic of different types of repeat sequence in *C. lanceolata* genome.

Type	Number	Length	Rate (%)
ClassI	13,217,000	9,755,331,388	86.78
ClassI/DIRS	954,220	928,392,143	8.26
ClassI/LINE	595,272	365,970,956	3.26
ClassI/LTR	485,367	411,052,389	3.66
ClassI/LTR/Copia	3,107,217	2,684,662,505	23.88
ClassI/LTR/Gypsy	4,233,916	4,764,617,838	42.38
ClassI/LTR|DIRS	140	65,857	0
ClassI/PLE|LARD	3,797,243	2,243,877,805	19.96
ClassI/SINE	3,706	805,063	0.01
ClassI/SINE|TRIM	832	875,820	0.01
ClassI/TRIM	33,556	21,340,727	0.19
ClassI/Unknown	5,531	4,488,270	0.04
ClassII	514,745	382,500,579	3.4
ClassII/Crypton	145	183,686	0
ClassII/Helitron	70,297	34,790,020	0.31
ClassII/MITE	4,567	2,757,596	0.02
ClassII/Maverick	19,158	11,853,833	0.11
ClassII/TIR	368,383	249,279,504	2.22
ClassII/Unknown	52,195	84,861,181	0.75
PotentialHostGene	183,062	118,181,144	1.05
SSR	26,634	30,066,332	0.27
Unknown	1,401,192	700,661,880	6.23
Total	13,941,441	10,377,888,279	92.31

Table S20. Statistic of LAI in each chromosomes.

Chr	LAI
Chr01	19.54
Chr02	20.00
Chr03	20.00
Chr04	20.09
Chr05	17.20
Chr06	16.94
Chr07	17.69
Chr08	18.00
Chr09	17.88
Chr10	17.82
Chr11	19.13

Table S21. Hypothetical WGDs, posterior mean of duplicate retention rate (q), and the Bayes Factor (K) to compare the likelihood of q = 0 (H0) to the likelihood of q > 0 (H1) using the Savage-Dickey density ratio.

Hypotheses	Relaxed branch-specific model		Critical branch-specific model	
	$\bar{q}$	K	$\bar{q}$	K
WGD1	0.11233	0.25962*	0.23817	0.06824**
WGD2	0.37868	0.04426**	0.17672	0.09463**
WGD3	0.08559	0.72805	0.00028	2899.97850
WGD4	0.03037	3.58108	0.00037	3564.65813
WGD5	0.59532	0.02821***	0.00332	246.95914

K < 1/102 or K < 0.01, decisive evidence against H_0****; K < 1/101.5 or K < 0.0316, very strong evidence against H_0***; K < 1/10 or K < 0.1, substantial evidence against H_0**; K < 1/100.5 or K < 0. 3162 substantial evidences against H_0*; K < 1, H_1 supported, not worth more than a bare mention; K > 1, H_0 supported.

Table S22 Details of the samples collection for evolutionary history analysis of *C. lanceolata*.

Group	Location	Amount	Latitude	Longitude	Altitude (m)
**GZH**	Huishui County, Guizhou province, China	10	25°15′~25°35′N	106°45′~106°57′E	900
**HNX**	Xinning County, Shaoyang City, Hunan Province, China	13	26°15′~26°55′N	110°18′~110°28′E	1300
**SXA**	Xinhua village, Ankang City, Shaanxi province, China	13	31°42′~31°59′N	108°07′~110°11′E	850
**FJM**	Meihuashan Nature Reserve, Longyan City, Fujian Province, China	12	25°15′~25°35′N	116°45′~116°57′E	1800
**SCD**	Dechang County, Sichuan Province, China	13	27°06′~27°35′N	101°12′~102°24′E	1050
**JSZ**	Xiashu County, Zhenjiang City, Jiangsu Province, China	12	32°04′~32°12′N	119°04′~119°15′E	260
**JXJ**	Jiulianshan Nature Reserve, Ganzhou City, Jiangxi Province, China	11	24°30′~24°52′N	114°33′~114°33′E	1200
**JXW**	Wengongshan Forest Park, Wuyuan City, Jiangxi Province, China	20	29°10′~29°11′N	117°46′~117°47′E	200
**TWB**	Baxianshan Nature Reserve, Taichung County, Taiwan Province, China	11	24°07′~24°09′N	120°15′~121°27′E	2000
**YNN**	Na Ri, Bac Kan province, Vietnam	13	23°07′~23°11′N	105°40′~105°44′E	1100

Table S25. List of putative flavonoid synthesis related genes in C. lanceolata genome.

Abbreviation	Gene name	Gene ID
PAL	Phenylalanone ammonia-lyase	Cl16939; Cl23281; Cl20012; Cl15629; Cl01003; Cl10224
C4H	Cinnamate 4-hydroxylase	Cl32060; Cl22669; Cl27969
4CL	Coumarate-4-CoA ligase	Cl32500; Cl18950; Cl23923; Cl29221; Cl20157; Cl11300; Cl08738; Cl36046; Cl18336; Cl23029; Cl33619; Cl25933; Cl21436; Cl09422; Cl29190; Cl26264; Cl16358; Cl02585; Cl23628; Cl07412; Cl15006; Cl27201; Cl16061; Cl01710; Cl33345; Cl22156; Cl13819; Cl22391; Cl33941; Cl36440; Cl15332; Cl34086; Cl04819; Cl06575; Cl24383
CHS	Chalcone synthase	Cl20463; Cl16987; Cl13810; Cl18516; Cl13939; Cl35852; Cl36558; Cl25812; Cl36434; Cl23043; Cl12414; Cl02164
CHI	chalcone-flavanone isomerase	Cl23637; Cl26105; Cl35530; Cl01348
F3H	flavanone-3-hydroxylase	Cl05230; Cl22835; Cl19511; Cl30186; Cl27251; Cl05548; Cl23387; Cl09496
F3'H	flavonoid 3'-hydroxylase	Cl26386; Cl20734; Cl00578
F3'5'H	flavonoid 3',5'-hydroxylase	Cl11848; Cl12701; Cl03636; Cl28266; Cl06640; Cl09151; Cl01764; Cl12036
FLS	Flavonol synthase	Cl13876; Cl03153; Cl27668; Cl21034; Cl01929; Cl15880; Cl05506; Cl33239; Cl21939; Cl14727; Cl09073; Cl34583
DFR	dihydroflavonols 4-reductase	Cl01163; Cl14835; Cl32921; Cl34319; Cl17992; Cl09745; Cl19190; Cl10044; Cl25990; Cl21164; Cl20763; Cl34597; Cl16924; Cl30627; Cl10196
LAR	leucoanthocyanidins reductase	Cl19924; Cl17849; Cl02972; Cl22572; Cl04043; Cl32845; Cl08749; Cl03800; Cl01405; Cl26343
ANS	anthocyanidin synthase	Cl28499
ANR	anthocyanidin reductase	Cl02683; Cl08429; Cl27585; Cl01531; Cl00030; Cl14400; Cl29306; Cl29276; Cl27034; Cl27658; Cl25902; Cl03085; Cl09863; Cl21775; Cl06423; Cl10344; Cl18261; Cl10859; Cl23026; Cl30270; Cl14318; Cl20814; Cl15108; Cl26250; Cl09989

Table S27. Blast results of 4 astringent seed related genes in *C. lanceolata* in the Shirasawa's assembly.

Gid in Shirasawa's assembly	Gid in assembly in this paper	Identity	Query coverage	Subject coverage
Clan1275G00001.1	Cl19924.1	99.76	99.92	99.92
Clan685G00029.1	Cl30227.1	99.41	99.85	90.67
Clan997G00018.1	Cl02910.1	99.05	99.88	99.88
-	Cl08429.1	-	-	-

Table S28. List of MADS-box genes identified in *C. lanceolata* genome.

Gene ID	Accession number	Location	Chr	ORF (bp)	Size (aa)	Group
Cl10262	MT103468	519587430-519650702	1	678	225	AG
Cl36126	MT103469	526300958-526365655	1	678	225	AG
Cl26543	MT103470	521807977-521978150	1	756	251	AG
Cl26063	MT103471	70041826-70094329	11	693	230	AG
Cl13850	MT103472	70233297-70328225	11	471	156	AG
Cl35439	MT103473	89314620-89335958	2	666	221	AG
Cl35065	MT103474	94642941-94733811	2	738	245	AGL6
Cl29520	MT103475	366538841-366706949	11	471	156	AGL6
Cl35039	MT103477	1392696700-1393060149	1	927	308	TM8
Cl22970	MT103478	1393706469-1394059060	1	636	211	TM8
Cl12097	MT103479	1392261362-1392431943	1	636	211	TM8
Cl11956	MT103480	201000156-201216026	1	636	211	TM8
Cl30647	MT103481	1395412787-1395891794	1	636	211	TM8
Cl17451	MT103482	426919818-427049392	10	465	154	TM8
Cl36264	MT103483	98633241-98845766	2	696	231	TM3
Cl34784	MT103484	596818838-596974165	6	708	235	SVP
Cl35571	MT103485	745221257-745358270	3	651	216	SVP
Cl01446	MT103486	746814647-747037347	3	720	239	SVP
Cl22549	MT103492	505217517-505359167	11	681	226	GpMADS4
Cl29629	MT103493	502196965-502224894	11	612	203	GpMADS4
Cl27678	MT103494	291146007-291466416	8	813	270	GGM13
Cl28889	MT103495	758894530-758896706	2	846	281	DEF/GLO
Cl22633	MT103496	750408487-750411952	2	588	195	DEF/GLO
Cl08169	MT103497	750587944-750592183	2	621	206	DEF/GLO
Cl27804	MT103498	759848871-759850338	2	525	174	DEF/GLO
Cl17023	MT103499	759890589-759892578	2	726	241	DEF/GLO
Cl14691	MT103500	746553907-746558043	2	642	213	DEF/GLO
Cl31654	MT103501	568065106-568070272	2	1062	353	MIKC*
Cl08187	MT103502	685865937-685938418	9	1152	383	MIKC*
Cl07566	MT103503	684871634-684879797	9	1131	376	MIKC*

Cl22501	MT103504	567007448-567046037	11	1131	376	MIKC*
Cl31662	MT103505	687228890-687234262	9	1146	381	MIKC*
Cl15999	MT103506	621864422-621864784	4	363	120	Mα
Cl24955	MT103507	716567113-716567590	4	318	105	Mα
Cl21596	MT103508	4079400-4079645	6	246	81	Mα
Cl00442	MT103509	143546-144199	/	654	217	Mα
Cl08495	MT103510	718529476-718530006	4	531	176	Mα
Cl21016	MT103511	695835786-695836619	1	834	277	Mα
Cl09032	MT103512	575513819-575514562	3	744	247	Mα
Cl29136	MT103513	574354493-574355266	3	774	257	Mα
Cl07839	MT103514	835739771-835740823	3	1053	350	Mα
Cl07629	MT103515	6402122-6403189	8	1068	355	Mα
Cl30941	MT103516	492704690-492705757	10	1068	355	Mα
Cl01366	MT103517	561794249-561795499	3	1251	416	Mγ
Cl19059	MT103518	979636832-979638091	3	1260	419	Mγ
Cl05223	MT103519	494123266-494124511	5	1182	393	Mγ
Cl18167	MT103520	979988133-979989272	3	1140	379	Mγ
Cl14558	MT103521	531037074-531037772	7	699	232	Mγ
Cl34141	MT103522	770338139-770339431	7	1293	430	Mγ
Cl00625	MT103523	980578572-980579186	3	615	204	Mγ

Table S29. The sequencing quality of the raw data from the nine Hi-C sequencing libraries.

Library	ReadSum	BaseSum	GC (%)	N (%)	Q20 (%)	Q30 (%)
L01	231,458,781	69,320,910,642	38.33	0	97.01	93.09
L02	216,574,783	64,867,446,176	38.26	0.03	94.86	88.84
L03	245,663,762	73,581,674,890	38.2	0.03	95.09	89.24
L04	214,400,226	64,223,373,938	38.23	0.01	95.81	90.6
L05	196,182,529	58,763,528,824	38.33	0.01	95.71	90.43
L06	248,263,233	74,357,707,184	38.34	0	97.05	93.21
L07	274,734,867	82,259,170,272	38.17	0	97	95.29
L08	251,334,974	75,261,718,710	38.17	0	96.16	94.07
L09	254,106,397	76,093,916,328	38.15	0	96.76	94.94
Total	2,132,719,552	638,729,446,964	/	/	/	/

Table S30. The statistics of the mapped efficiency of the pairs on the nine Hi-C sequencing library data.

Library	Type	Number	Ratio (%)
L01	Unique Paired Alignments	74,149,083	100
	Valid Interaction Pairs	68,649,601	92.58
	Dangling End Pairs	3,640,918	4.91
	Re-ligation Pairs	405,084	0.55
	Self-cycle Pairs	99,755	0.13
	Dumped Pairs	1,353,725	1.83
L02	Unique Paired Alignments	67,062,534	100
	Valid Interaction Pairs	60,859,580	90.75
	Dangling End Pairs	4,395,499	6.55
	Re-ligation Pairs	415,483	0.62
	Self-cycle Pairs	110,980	0.17
	Dumped Pairs	1,280,992	1.91
L03	Unique Paired Alignments	75,942,872	100
	Valid Interaction Pairs	68,950,679	90.79
	Dangling End Pairs	4,966,975	6.54
	Re-ligation Pairs	468,638	0.62
	Self-cycle Pairs	124,476	0.16
	Dumped Pairs	1,432,104	1.89
L04	Unique Paired Alignments	68,132,268	100
	Valid Interaction Pairs	62,320,281	91.47
	Dangling End Pairs	3,960,157	5.81
	Re-ligation Pairs	403	0.59
	Self-cycle Pairs	116,034	0.17
	Dumped Pairs	1,333,095	1.96
L05	Unique Paired Alignments	60,413,157	100
	Valid Interaction Pairs	54,939,692	90.94
	Dangling End Pairs	3,799,713	6.29
	Re-ligation Pairs	371,197	0.61
	Self-cycle Pairs	92,730	0.15
	Dumped Pairs	1,209,825	2.00
L06	Unique Paired Alignments	80,471,301	100
	Valid Interaction Pairs	74,585,391	92.69
	Dangling End Pairs	3,831,391	4.76
	Re-ligation Pairs	480,089	0.6
	Self-cycle Pairs	87,930	0.11
	Dumped Pairs	1,486,500	1.85
L07	Unique Paired Alignments	89,802,324	100
	Valid Interaction Pairs	82,996,299	92.42
	Dangling End Pairs	4,306,629	4.8

	Re-ligation Pairs	475,349	0.53
	Self-cycle Pairs	146,436	0.16
	Dumped Pairs	1,877,611	2.09
L08	Unique Paired Alignments	82,128,266	100
	Valid Interaction Pairs	76,225,099	92.81
	Dangling End Pairs	3,705,017	4.51
	Re-ligation Pairs	481,714	0.59
	Self-cycle Pairs	86,016	0.1
	Dumped Pairs	1,630,420	1.99
L09	Unique Paired Alignments	84,783,347	100
	Valid Interaction Pairs	79,165,448	93.37
	Dangling End Pairs	3,374,676	3.98
	Re-ligation Pairs	474,558	0.56
	Self-cycle Pairs	76,547	0.09
	Dumped Pairs	1,692,118	2.0

Unique Paired Alignments: The only read pairs aligned to the assembled genome; Valid Interaction Pairs: Valid Read Pairs; Dangling End Pairs: Data of end suspension type in invalid read pairs; Re-ligation Pairs: Dara of adjacent connection type in invalid read pairs; Self-circle Ligation Pairs: The invalid data belongs to read pairs of self-connecting type; Dumped Pairs: The invalid data belongs to other undefined read pairs.

Table S31. Primer designs with *Spe I* restriction sites for the candidate genes infusion cloning.

Primer Name	Primer sequence
ANR3301-F	5’TGACCTCGAGACTAGTATGAGTTGCACTAAGAAGGT3’
ANR3301-R	5’TGTAGTCCATACTAGTGTTACCGACATCATTAGAGC3’
LAR3301-F	5’TGACCTCGAGACTAGTATGGCCTGTGCTCCCAAAGT3’
LAR3301-R	5’TGTAGTCCATACTAGTAAGGTACTGGTTGAAAAAAT3’
DET23301-F	5’TGACCTCGAGACTAGTATGGCGCATGTTCTGCAACA3’
DET23301-R	5’TGTAGTCCATACTAGTGAATATGAAGGGGAAAAGAG3’
BZR13301-F	5’TGACCTCGAGACTAGTATGTCCACCCGCTCCGTAAT3’
BZR13301-R	5’TGTAGTCCATACTAGTGGGACAAAAATGTTGAGACA3’

Method S1. Observation and determination of the structure of astringent and germinating seeds in different development stages.

Samples were collected from the Youxi National Forest Farm, Fujian Province, China. The geographical coordinates of the orchard are 25°50 ' – 26°26' N, 117°48 ' – 118°39' E. In 2017, ten healthy individuals with similar height, diameter, and annual astringent seed incidence rates were randomly selected from the same orchard area. The date of artificial supplementary pollination (1st March) was recorded as day 0. After 60 days, three cones from each individual were collected every 5 days until the seeds were harvested. We observed the structures of the astringent and germinating seeds at 95, 105, 115, 125, 135, 145, 155, 165, 175, and 185 d using an optical microscope. During observation, the seed coats were quickly and carefully removed and the embryos were examined under 20× magnification using an optical microscope. Based on their colour, lustre, and hardness, embryos at different growth stages were judged to be astringent or germinating seeds.

Method S2. Metabolome detection at different stages of astringent seeds and germinating seeds.

2.1 Sample collection, processing, and storage

The samples used for transcriptomic analysis were also used for the metabolomic assays. The sampling collection, processing, and storage are described in the 'Transcriptomic data and analysis' in the 'Online methods' section. Please refer to this section for details regarding sampling methods.

2.2 Sample preparation and extraction

All freeze-dried samples were crushed using a mixer mill (MM 400, Retsch) with zirconia beads for 1.5 min at 30 Hz. One hundred milligrams of powder of each sample was weighed and extracted overnight at 4°C with 1.0 mL 70% aqueous methanol. Following centrifugation at 10000 × *g* for 10 min, the extracts were absorbed (CNWBOND Carbon-GCB SPE Cartridge, 250 mg, 3 mL; ANPEL, Shanghai, China, www.anpel.com.cn/cnw) and filtered (SCAA-104, 0.22 μm pore size; ANPEL, Shanghai, China, http://www.anpel.com.cn/) before LC-MS analysis.

2.3 HPLC and ESI-Q TRAP-MS/MS conditions

The sample extracts were analysed using an LC-ESI-MS/MS system (Shim-pack UFLC SHIMADZU CBM30A system, www.shimadzu.com.cn/; MS, Applied Biosystems 4500 Q TRAP, www.appliedbiosystems.com.cn/). The analytical conditions were as follows, HPLC: column, Waters ACQUITY UPLC HSS T3 C18 (1.8 μm, 2.1 mm × 100 mm); solvent system, water (0.04% acetic acid): acetonitrile (0.04% acetic acid); gradient program, 95:5 V/V at 0 min, 5:95 V/V at 11.0 min, 5:95 V/V at 12.0 min, 95:5 V/V at 12.1 min, 95:5 V/V at 15.0 min; flow rate, 0.40 mL/min; temperature, 40°C; injection volume: 5 μL. The effluent was alternately connected to an ESI-triple quadrupole linear ion trap-MS (QTRAP)-MS.

LIT and triple quadrupole (QQQ) scans were acquired on a triple quadrupole-linear ion trap mass spectrometer (Q TRAP), API 4500 Q TRAP LC/MS/MS System, equipped with an ESI Turbo Ion-Spray interface, operating in positive ion mode, and controlled by the Analyst 1.6 software (AB Sciex). The ESI source operation parameters were as follows: an ion source, turbo spray; source temperature, 550°C; ion-spray voltage (IS), 5500 V; ion source gas I (GSI), gas II(GSII), and curtain gas (CUR) were set at 55, 60, and 25.0 psi, respectively; the collision gas (CAD) was high. Instrument tuning and mass calibration were performed with 10 and 100 μmol/L polypropylene glycol solutions in QQQ and LIT modes, respectively. QQQ scans were acquired as multiple reaction monitoring (MRM)

experiments with the collision gas (nitrogen) set at 5 psi. The DP and CE for individual MRM transitions were further DP and CE optimization.

2.4 Qualitative and quantitative analyses of metabolites

Based on the self-built database and public databases (HMDB, http://www.hmdb.ca/; METLIN, https://metlin.scripps.edu/; and KEGG, http://www.kegg.jp/kegg/compound/), qualitative analysis was carried out according to the second spectral information. The isotope signals, repeated signals containing K^+, Na^+, and $NH4^+$ ions, and repeated signals of fragment ions with other larger-molecular-weight substances were removed.

Quantification was performed using the MRM model with a triple four-stage rod mass spectrometer. After obtaining the metabolite spectrum analysis data of different samples, the peak integral area was calculated for all mass spectrum peaks and integral correction was performed for the spectrum peaks of the same metabolite in different samples.

2.5 Identification and verification of different expressive metabolites

Analyst v1.6.1 software was used to process raw mass spectrometry data. The MultiaQuant software was used to integrate and correct the chromatographic peaks. The area of each chromatographic peak represented the relative content of the corresponding compounds. Finally, chromatographic peak integral area data were derived. Principal component analysis, orthogonal partial least squares discriminant analysis (OPLS-DA), and Pearson correlation coefficient calculations were performed using RStudio software (www.r-project.org/).

Metabolites from different groups were preliminarily screened based on variable importance in the project (VIP) value obtained from the OPLS-DA model. The fold-change values were combined to screen differentially expressed metabolites (DEMs). The metabolites with FC of ≥ 2.0 or FC of ≤ 0.5 were considered to differ more than twice or less than 0.5 between the Astringent Seed (AS) and Germinate Seed (GS) groups, while a VIP value of ≥ 1.0 was indicative of a significant effect on the classification of samples in each group in the OPLS-DA model. Metabolites that fit both conditions were selected as DEMs.

To verify the accuracy of the relative quantification results generated from LC-MS signals, 16 metabolites were randomly selected for further HPLC quantification. The HPLC conditions were the same as those described in Section 2.3. Standards for the selected metabolites were obtained from the Shinemro Chemical Platform (http://www.shinemro.com).

Method S3. Transgenic verification in *A. thaliana*.

We employed the Agrobacterium-mediated transformation method to introduce candidate genes associated with astringent seed formation regulation into *Arabidopsis thaliana*. These included *ClLAR* and *ClANR* from the flavonoid biosynthesis pathway, and *ClDET2* and *ClBZR1* from the brassinosteroid signaling pathway. Phenotypic changes following overexpression of these genes were then observed. The procedure is outlined below:

Primers flanking the *Spe I* restriction sites in the CDS region of the candidate genes were designed based on genomic data (**Table S31**). After PCR amplification, the products were cloned using the Infusion cloning method and sequenced for alignment. Amplification was then carried out using Tks Gflex DNA Polymerase. The desired PCR bands were purified with the MiniBEST Agarose Gel DNA Extraction Kit V.4.0. Plasmid pCambia3301 was digested with Spe I, and the vector DNA was purified. In-Fusion reactions were conducted following the In-Fusion HD Cloning Kit protocol, with the following components: 2.0 μl of vector DNA (50 ng/μl), 1.0 μl of PCR product (50 ng/μl), 2.0 μl of 5×

In-Fusion HD Enzyme Premix, and 5.0 μl of dH2O. The reaction was incubated at 50°C for 15 minutes. A 2.5 μL aliquot of the reaction mixture was heat-transformed into E. coli Competent Cells JM109 and incubated overnight at 37°C. After positive colonies appeared, the bacteria were cultured, and the recombinant plasmid was extracted using the MiniBEST Plasmid Purification Kit.

Agrobacterium transformation was performed using the freeze-thaw method. The GV3101 strain was cultured in autoclaved YEB medium until the OD600 reached 0.8-1.0, indicating activation. Recombinant plasmid (800 ng) was mixed with 100 μl of activated competent cells, vortexed briefly, and incubated on ice for 30 minutes. The mixture was then frozen in liquid nitrogen for 1 minute, followed by a 5-minute heat shock at 37°C and a 3-minute incubation on ice. This procedure was repeated once. Next, 400 μl of antibiotic-free YEB medium was added, and the mixture was incubated in the dark at 28°C with shaking at 200 rpm for 4 hours. After a 1-minute centrifugation at 10,000 rpm, 100 μl of the supernatant was used to resuspend the Agrobacterium, which was then plated on YEB agar. Once positive colonies appeared, they were cultured in YEB medium until the OD600 reached 0.8-1.0, at which point the culture was ready for infection.

The inflorescence infection method was used to transform *A. thaliana* during its flowering stage. The plants were submerged for 1 minute in an infection solution mixed with L/LSilwet L-77 transformation liquid and then incubated in the dark for 24 hours. After incubation, the plants were cultured until seed maturation, at which point the seeds were harvested, sown, and grown to obtain T1 generation seedlings. Once cotyledons emerged, the seedlings underwent resistance screening with a 1,000-fold dilution of Basta solution. Resistant seedlings were transplanted, and successful gene integration was confirmed through PCR using the TransDirect Plant Tissue PCR Kit. The PCR reaction mixture contained 4.0 μl of lysis buffer, 0.4 μl of each primer, 10 μl of 2 × TransDirect PCR SuperMix, and 5.2 μl of dH_2O.

Then we allowed the T1 generation transgenic *A. thaliana* seedlings to mature and produce seeds. The seeds were then analyzed phenotypically, including evaluations of seed appearance and germination rate, to explore the potential role of the candidate genes in reproductive growth.

Method S4. Determination of aluminum in *C. lanceolata*.

We analysed the aluminum content of Chinese fir leaves at different developmental stages. All materials from ten individuals were randomly selected from a third-generation seed orchard at the Youxi National Forest Farm, Fujian Province, China. Five groups of materials were collected, with 10 replicates per group. The five materials were as follows: young (YL) and old leaves from living branches, leaves that survived for one year (WL1) and five years (WL2) in withered branches, and two months of fallen leaves (WL3) from withered branches.

After 1 h of deactivation of enzymes at 105°C, all the samples were dried at 70°C until a constant weight was obtained. The dry samples were ground, crushed, and then filtered through a 0.5 mm sieve. Samples from each group were accurately weighted (0.2 g respectively, and digested with nitric acid and hydrogen peroxide solution using a microwave digestion system (Multiwave ECO). The digested solution was diluted with pure water to a final volume of 50 mL and cooled to room temperature. Subsequently, the concentration of Al ions in each sample group was determined using an inductively coupled plasma method with a PE Optima 8000 system (PerkinElmer, USA).
