# Supplementary material for: Cunninghamia lanceolata genome illuminates the evolutionary dynamics of gymnosperms
Source: Cell Rep. 2026 Jun 18;45(7):117566. doi: 10.1016/j.celrep.2026.117566 (PMC13415678; doi:10.1016/j.celrep.2026.117566)
Supplement: Document S2. Article plus supplemental information [file mmc10.pdf]

Cell Reports

Article

Cunninghamia lanceolata genome illuminates the evolutionary dynamics of gymnosperms

Graphical abstract

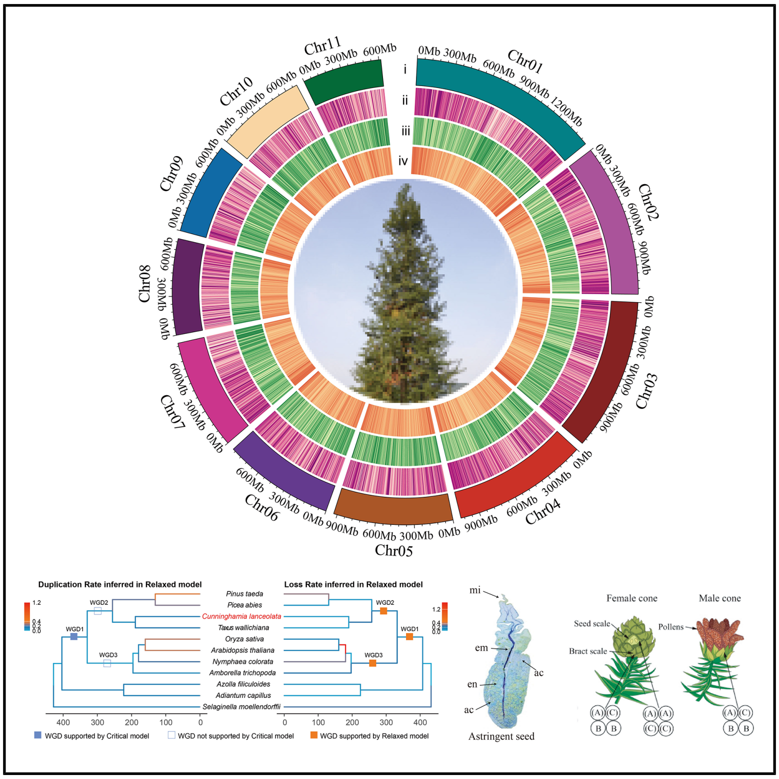

Highlights

- A chromosome-level genome of *Cunninghamia lanceolata* is presented
- Cupressaceae forms a sister group to Gntales-Welwitschia and pine clade among conifer
- No ancient polyploidization exists in *C. lanceolata*, seed-plant-shared WGD is confirmed
- Astringent seeds formation and reproductive organs' evolution are explored

Authors

Si-Zu Lin, Yu Chen, Chao Wu, ..., Ray Ming, Yves Van de Peer, Zhong-Jian Liu

Correspondence

zhenghk@biomarker.com.cn (H.-K.Z.),
rayming@illinois.edu (R.M.),
yves.vandepeer@psb.vib-ugent.be (Y.V.d.P.),
zjliu@fafu.edu.cn (Z.-J.L.)

In brief

Lin et al. present a 11.24 Gb chromosome-level genome of *Cunninghamia lanceolata*, a key Cupressaceae species in China's timber production. The study also explores gymnosperm evolutionary dynamics, seed-plant-shared WGD events, population evolutionary history, astringent seeds formation, and reproductive organs' evolution.

Lin et al., 2026, Cell Reports *45*, 117566
July 28, 2026 © 2026 The Author(s). Published by Elsevier Inc.
https://doi.org/10.1016/j.celrep.2026.117566

Cell Reports

CellPress
OPEN ACCESS

Article

Cunninghamia lanceolata genome illuminates the evolutionary dynamics of gymnosperms

Si-Zu Lin,[1,12] Yu Chen,[1,12] Chao Wu,[1,12] Wei-Hong Sun,[2,12] Zhen Li,[3,4,12] Heng-Chi Chen,[3,4] Jie-Yu Wang,[1] Chang-Mian Ji,[5] Shu-Bin Li,[1,6] Zhi-Wen Wang,[7] Wen-Chieh Tsai,[1] Xiang-Qing Ma,[1] Si-Ren Lan,[1] Fei-Ping Zhang,[1] Ya-Cong Xie,[1] Lei Yao,[1] Yan Zhang,[1] Meng-Meng Lü,[1] Jia-Jun Zhang,[1] Di-Yang Zhang,[1] Yi-Quan Ye,[1,6] Xia Yu,[1] Shan-Shan Xu,[1,6] Zhi-Hui Ma,[1,6] Guo-Chang Ding,[1,6] Guang-Qiu Cao,[1,6] Zong-Ming He,[1,6] Peng-Fei Wu,[1] Kai-Min Lin,[1] Ai-Qin Liu,[1] Yan-Qing Lin,[1] Shao-Ning Ruan,[1] Bao Liu,[1] Shi-Jiang Cao,[1] Li-Li Zhou,[1] Ming Li,[1] Peng Shuai,[1] Xiao-Long Hou,[1] Yi-Han Wu,[1] Nuo Li,[1] Sheng Xiong,[1] Yang Hao,[1] Zhuang Zhou,[1] Xue-Die Liu,[1] Dan-Dan Zuo,[1] Jia Li,[1] Pei Wang,[1] Jian Zhang,[1] Ding-Kun Liu,[1] Gui-Zhen Chen,[1] Jie Huang,[1] Ming-Zhong Huang,[1] Yuan-Yuan Li,[1] Qin-Yao Zheng,[1] Xue-Wei Zhao,[1] Xiang Zhao,[7] Wen-Ying Zhong,[7] Xue-Wen Zhang,[5] Zheng-Bao Xia,[5] Ying Yu,[5] Zhi-Wei Liu,[5] Hong-Kun Zheng,[5,*] Ray Ming,[8,*] Yves Van de Peer,[3,4,9,10,*] and Zhong-Jian Liu[1,11,13,*]

[1]Chinese Fir Engineering Technology Research Center of the State Forestry and Grassland Administration at College of Forestry, and Key Laboratory of National Forestry and Grassland Administration for Orchid Conservation and Utilization at College of Landscape Architecture and Art, Fujian Agriculture and Forestry University, Fuzhou 350002, China
[2]College of Biology and Agriculture, Zunyi Normal University, Zunyi 563006, China
[3]Department of Plant Biotechnology and Bioinformatics, Ghent University, 9052 Ghent, Belgium
[4]VIB Center for Plant Systems Biology, VIB, 9052 Ghent, Belgium
[5]Technical Department, Biomarker Technologies Corporation, Beijing 101300, China
[6]Key Laboratory for Forest Adversity Physiological Ecology and Molecular Biology, The Education Department of Fujian Province, Fuzhou 350002, China
[7]PubBio-Tech, Wuhan 430070, China
[8]Fujian Agriculture and Forestry University and University of Illinois at Urbana-Champaign–School of Integrative Biology Joint Center for Genomics and Biotechnology, Fujian Agriculture and Forestry University, Fuzhou, China
[9]Centre for Microbial Ecology and Genomics, Department of Biochemistry, Genetics and Microbiology, University of Pretoria, Pretoria 0028, South Africa
[10]College of Horticulture, Academy for Advanced Interdisciplinary Studies, Nanjing Agricultural University, Nanjing, China
[11]Guangzhou Institute of Forestry and Landscape Architecture, Guangzhou 510405, China
[12]These authors contributed equally
[13]Lead contact
*Correspondence: zhenghk@biomarker.com.cn (H.-K.Z.), rayming@illinois.edu (R.M.), yves.vandepeer@psb.vib-ugent.be (Y.V.d.P.), zjliu@fafu.edu.cn (Z.-J.L.)
https://doi.org/10.1016/j.celrep.2026.117566

SUMMARY

Cupressaceae , a gymnosperm family, draws attention due to its controversial phylogenetic position. Here, we present a comprehensive genome analysis of Chinese fir (*Cunninghamia lanceolata*), a Cupressaceae species, to enhance our understanding of gymnosperm evolution. The 11.24 Gb assembled genome, shaped by inefficient long terminal repeat removal, offers insights into its phylogenetic position. Phylogenetic analysis refines gymnosperm relationships between Cycads-Ginkgo and their relation to Gnetales-Pinaceae. Whole-genome duplication (WGD) analysis reveals no evidence for an ancient polyploidization event in the lineage of *C. lanceolata*, and confirms a seed-plant-shared WGD event. We also explore genomic evidence to explain the population history and adaptability of *C. lanceolata*, including potential glacial refugia, dispersal centers, and unique sterility. Furthermore, the refined (A)B(C) model for reproductive organ development in *C. lanceolata* has broader applications across gymnosperms. This study provides a valuable genome sequence and contributes to the understanding of gymnosperm evolution.

INTRODUCTION

Gymnosperms, encompassing extant taxa, such as cycads, Ginkgo, conifers, and gnetophytes[1–4] first emerged during the Late Devonian period, approximately 360 million years ago (Mya).[5] Despite their ancient origins, gymnosperms today comprise slightly over 1000 species, significantly fewer than the 354 000 angiosperm species.[6] However, fossil records reveal that gymnosperms were once far more diverse, highlighting their historical radiation and extinction, and also indicating that the living gymnosperms represent only a fraction of their past diversity.[7,8] This realization presents challenges in accurately reconstructing

© 2026 The Author(s). Published by Elsevier Inc.
This is an open access article under the CC BY license (http://creativecommons.org/licenses/by/4.0/).

the phylogenetic relationships of gymnosperms, which is crucial for unraveling the fascinating scientific inquiry into the origin of seed plants.

In the traditional plant systematics, gymnosperm classifications are based on morphological characters, such as leaf shape, bract and scale arrangement. Within this framework, the genus *Cunninghamia* has been classified into the family Taxodiaceae, primarily based on oligo-specific or mono-specific evidence.[9,10] The family Taxodiaceae further belongs to the Pinopsida group, alongside Pinaceae and Cupressaceae. Recent studies, however, have classified the genus *Cunninghamia* into the subfamily Cunninghaminae, which belongs to a well-recognized monophyletic group, i.e., the expanded Cupressaceae, alongside the other six subfamilies, Taiwanioideae, Athrotaxidoideae, Sequoioideae, Taxodiaceae, Callitroideae, and Cupressoideae.[1] This consolidation is supported by evidence from various sources, including fossil records, morphological anatomy, chemical structure, and molecular evidence, such as gene fragments or organelle genomes.[2,9–13]

That said, although taxonomists have classified gymnosperms as Cyadopsida, Ginkgoopsida, and Pinopsida, with Pinopsida including Cupressidae, Pinidae and Gnetidae, the phylogenetic relationships among extant gymnosperms remain elusive.[1] Indeed, various hypotheses have been proposed regarding the phylogenetic relationships within gymnosperms, particularly concerning the placements of gnetophytes among the so-called Pinopsida group. For instance, the "Gnepine" hypothesis suggests that gnetophytes are sister to Pinaceae,[14,15] whereas the "Gnecup" hypothesis groups gnetophytes with non-Pinaceae conifers (Cupressophytes)[16] and the "Gnetifer" hypothesis places gnetophytes as sister to all conifers in the Pinopsida group.[17] Additionally, gnetophytes have also been placed as a sister group to all other gymnosperms.[18,19]

To more accurately infer the phylogenetic relationships among extant gymnosperms, obtaining more complete genomic information is a reliable approach. Such comprehensive genetic data can provide a holistic perspective, enabling scientifically grounded inferences.[20–22] Until recently, the genomes of several gymnosperm lineages, including conifers, such as Pinaceae: *Picea abies*,[23] *Picea glauca*,[24] *Pinus taeda*,[25,26] *Pinus tabuliformis*,[27] *Pinus densiflora*,[28] *Larix kaempferi*,[29] Taxaceae: *Taxus chinensis* (a synonym of *Taxus wallichiana*),[30] Ginkgo: *Ginkgo biloba*,[31,32] cycads: *Cycas panzhihuaensis*,[2] gnetophytes:*Gnetum montanum*[33] and *Welwitschia mirabilis*,[34] and Cupressaceae: *Metasequoia glyptostroboides*,[35] *Fokienia hodginsii*[36] have been successfully sequenced. Among the extant Cupressaceae species, the genus *Cunninghamia*, comprising only two species *C. lanceolata* and *C. konishii*, is the sister group to all the other genera in Cupressaceae. This makes *Cunninghamia* an especially important representative for genomic sequencing, both to improve our understanding of Cupressaceae evolution and to provide a foundational reference for comparative studies across gymnosperms.

The family Cupressaceae stands out as the sole gymnosperm lineage with widespread distribution across both the northern and southern hemispheres. *C. lanceolata*, commonly referred to as "Sha Mu" in China, predominantly thrives in southern China, with additional occurrences noted in northern Vietnam.[3] Historical records trace the artificial cultivation of *C. lanceolata* back over two thousand years, as documented in ancient Chinese texts, such as the "Erya." Today, owing to its rapid growth, high-quality wood, and resistance to decay, *C. lanceolata* is the most widely planted timber species in China. Its plantation area exceeds 9.90 million hm^2, accounting for approximately one-quarter of the total artificial forest area in China, with a forest stock of 755 million m^3, i.e., one-fourth of all man-made forest stock. Given its economic and ecological importance, improving the genetic understanding of *C. lanceolata* is a key objective for breeders and foresters to enhance timber production and carbon sequestration capabilities.

Recently, Shirasawa et al. published a genome assembly of *C. lanceolata* of Japan that only includes contig-level assembly without annotation. The data were generated employing high-fidelity (HiFi) long-read sequencing technology, which yielded 2,472 assembled contigs spanning a genome size of 12.04 Gb with an N50 value of 11.7 Mb.[37] In this study, we present a high-quality chromosomal-level genome assembly of *C. lanceolata*, which was scaffolded using high-throughput chromosome conformation capture (Hi-C) data. To facilitate genomic comparison, we annotated the *C. lanceolata* genome assembly of Shirasawa et al. SNP calling was performed using the *C. lanceolata* genome assembly of Shirasawa et al. and our population resequencing data of *C. lanceolata* to infer the evolutionary relationships among its populations. Our results provide a valuable resource for exploring the evolutionary history of Cupressaceae and offer a genomic foundation for significant advancements in forestry, breeding, and environmental applications such as carbon sequestration.

RESULTS

Genome sequencing, assembly, and annotation

To construct a chromosome-level genome assembly of *C. lanceolata*, genomic DNA was extracted from an adult individual. The *K*-mer analysis indicated that the genome size of *C. lanceolata* was approximately 10.42 Gb, with a high level of heterozygosity (0.69%) (Figure S1). A *de novo* assembly of the *C. lanceolata* genome was performed using PacBio reads (1,113.16 Gb in total) along with Illumina whole-genome sequencing reads (516.42 Gb in total) (Tables S1 and S2), resulting in an assembled genome of 11.24 Gb with a contig N50 value of 2.16 Mb (Table 1; Table S3). *C. lanceolata* is a diploid plant containing 11 chromosomes ($2n = 2\times = 22$).[38] To further refine the assembly, we employed the high-throughput/resolution chromosome conformation capture (Hi-C) technology, assigning 10.89 Gb scaffolds to the 11 *C. lanceolata* chromosomes (Table 1; Figure S2; Table S4). The lengths of these 11 chromosomes ranged from 0.64 to 1.55 Gb, with a scaffold N50 value of 927.89 Mb (Figure 1A, Table 1; Tables S4 and S5). To verify the completeness of our genome assembly, we mapped all the Illumina reads to the chromosome-scale assembly and obtained a mapping rate of 92.83% (Table S6). A total of 37,225 protein-coding genes were predicted through integrating *ab initio* predicted protein-coding genes (Table 1), homologous protein alignments, and transcriptome RNA-Seq data integration. Among these, 34,559 (92.84%) protein-coding genes could be

Table 1. Assembly and annotation statistics of the genome of *C. lanceolata*

Species	*C. lanceolata*
Assessment of genome size (Gb)	10.42
Contig N50 (bp)	2,155,103
Total length of Contig (bp)	11,242,038,337
Scaffold N50 (bp)	927,886,105
Total length of Scaffold (bp)	11,244.041,337
Total length of sequences anchored to chromosomes (bp)	10,893,531,473
Assembled genome BUSCO (%)	93.64
Number of protein-coding genes	37,225
Annotated protein BUSCO (%)	89.60
Total size of TEs (bp)	10,377,888,279
TE in genome (%)	92.31

functionally annotated (Tables S7 and S8). Additionally, we identified 50 microRNAs (miRNAs), 3,955 transfer RNAs (tRNAs), and 2,930 ribosomal RNAs (rRNAs) (Table S9). The Benchmarking Universal Single-Copy Orthologs (BUSCO, v5.8.2) assessment based on gymnosperm_0db 10 lineage dataset[39] revealed that the completeness of genes was 93.64%, and 89.60% in the genome and protein model, respectively (Table 1). These complete BUSCO values were comparable to those reported recently for *F. hodginsii*,[36] indicating a high quality of the *C. lanceolata* genome.

Upon comparing the genomes of 19 plant species, we observed 1,860 expanded gene families within the *C. lanceolata* genome, with 120 of these families showing significant expansion ($p < 0.01$) (Figure 1B; Table S10; Figure S3). The Kyoto Encyclopedia of Genes and Genomes (KEGG) and Gene Ontology (GO) enrichment analyses revealed that the significantly expanded gene families were especially enriched in KEGG pathways related to the biosynthesis of secondary metabolites, such as monoterpenoid (map00902) and betalain (map00965) (Table S11), as well as in GO terms associated with lignin biosynthetic/metabolic process (GO:0009809/GO:0009808) and terpene synthase activity (GO:0010333) (Table S12). Lignin biosynthesis involves the hydrolysis of its precursor glucoside by beta-glucosidase, followed by catalysis by the laccase and peroxidase systems. Interestingly, beta-glucosidase (OG0000016) and laccase (OG0000039) exhibited significant expansion, with 58% (28/48) and 51% (20/39) of their copies being tandem repeats, respectively (Tables S13, S14, and S15).

Compared with the genome assembly reported by Shirasawa et al. (12.04 Gb with a contig N50 of 11.70 Mb),[37] our assembly is of comparable size (11.24 Gb with a contig N50 of 2.16 Mb). However, our genome assembly achieves a higher BUSCO completeness score of 93.64%, versus 89.10% for Shirasawa et al. (Table S16), and has been further anchored to the chromosome level with comprehensive functional annotation. Using NUCmer (v4.0.0rc1),[40,41] we aligned the Shirasawa et al. assembly to our chromosomes, revealing per-chromosome coverage ranging from 91.83% to 95.20% (Table S17). Visualization of the alignments confirmed that our chromosomal assemblies were fully covered by the sequences of Shirasawa et al., demonstrating strong concordance between the two assemblies (Figure S4).

We performed genome annotation for the *C. lanceolata* genome of Shirasawa et al. using a combined strategy incorporating *de novo* prediction, homology-based searches, and transcriptome alignment. A total of 37,944 protein-coding genes were predicted in this annotated genome (Tables S16, S17, and S18), which is highly consistent with the 37,225 protein-coding genes identified in our genome assembly. Subsequently, we conducted a comparative analysis between the two genomes. Specifically, 33,705 genes from our assembly were detected in the genome of the *C. lanceolata* assembly of Shirasawa et al., and conversely, 33,729 genes from their assembly were identified in our genome. These overlapping genes were defined as the shared gene set between the two accessions (Figure S5). A total of 3,623 genes were uniquely present in our assembly but absent from the genome of the *C. lanceolata* assembly of Shirasawa et al.; these were classified into three categories: true gene loss (11 genes), highly divergent sequences (433 genes), and sequences with altered start or stop codons (3,179 genes). Functional enrichment analysis revealed that these unique genes were mainly enriched in GO terms related to DNA integration and KEGG pathways associated with base excision repair. In contrast, 4,215 genes specific to the *C. lanceolata* reported by Shirasawa et al. assembly were missing from our genome, exhibiting a similar classification pattern: 245 true gene losses, 354 highly divergent genes, and 3,616 genes with start/stop codon mutations. Functional annotation showed that these specific genes were enriched in distinct functional pathways, such as the GO term for inorganic molecular transmembrane transporter activity and KEGG pathways related to DNA polymerase function (Figure S6).

Genome expansion is associated with long introns and retrotransposons

An important feature of the predicted gene structure of *C. lanceolata* was the abundance of long introns with an average length of 6,482 bp (Table S18). Additionally, repetitive sequences occupy a substantial portion of the *C. lanceolata* genome, totaling 10.38 Gb and accounting for 92.31% of the assembled genome (Table 1). The proportion is notably higher than that observed in *Gnetum* (85.93%),[34] *Taxus* (76.09%),[30] and *Ginkgo* (76.58%).[35]

Among these repetitive sequences, LTRs are most prevalent, accounting for 69.92% of the genome (Ty3/Gypsy 42.38%; Ty1/Copia 23.88%), followed by dictyostelium intermediate repeat sequences (DIRSs, 8.26%) and long interspersed nuclear elements (LINEs, 3.26%) (Table S19). The LTR assembly index (LAI) values, ranging from 16.94 to 20.09 across chromosomes (Table S20) are higher than those in *F. hodginsii*,[36] further indicating the reference-level continuity of our assembled genome and reflecting the substantial LTR contribution to genome structure.

We also found that the ratio of solo-LTR to intact-LTR of *C. lanceolata* is 0.97 (100,395: 104,515), higher than that in Pinaceae species such as *P. tabuliformis* (0.16), *P. abies* (0.16), and

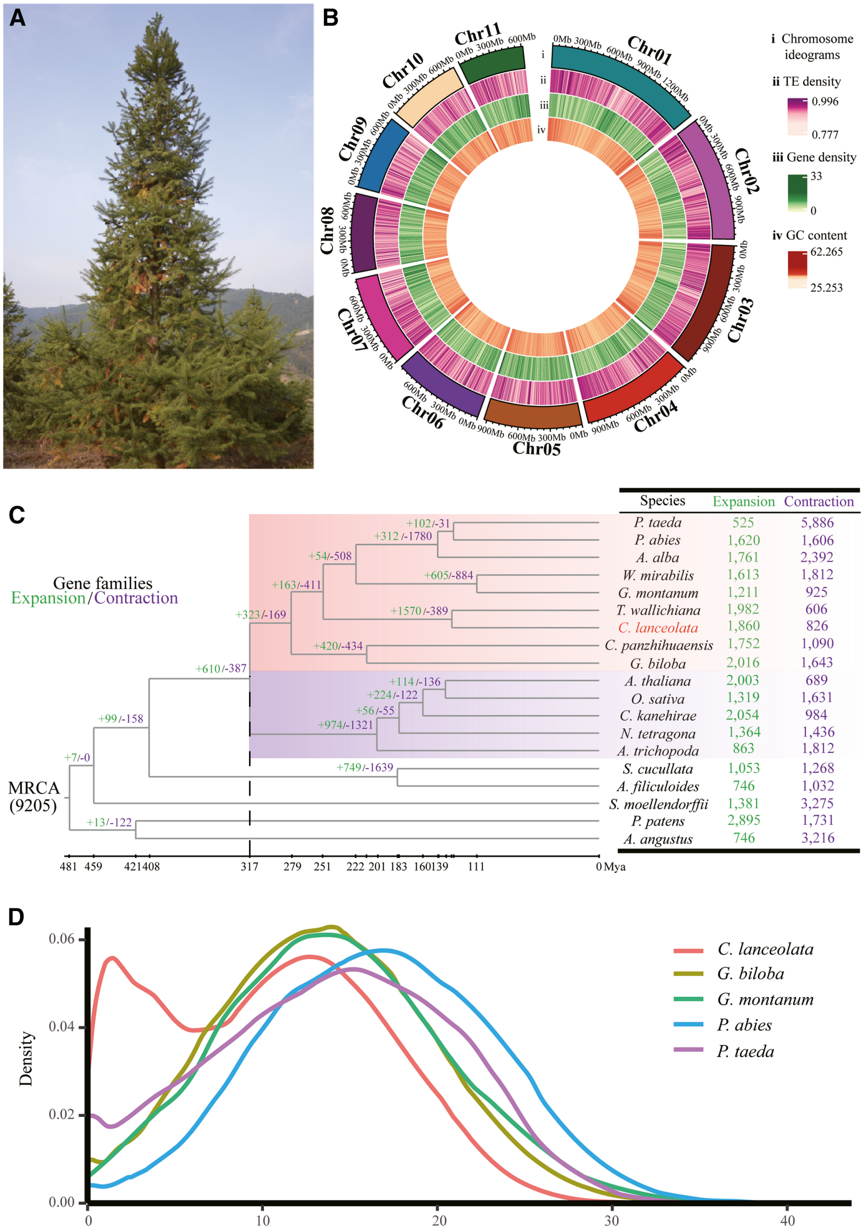

Figure 1. Genomic evolutionary history of *Cunninghamia lanceolata*

(A) An overview picture of the *C. lanceolata*.

(B). Genomic landscape of the 11 assembled pseudochromosomes.

Track i represents the length of the pseudochromosomes (Mb); tracks ii-iv represent repeat element density, gene density distribution, and GC content, respectively. These metrics were calculated within 300 Mb windows.

(C). Phylogenetic tree, gene family expansion and contraction, and divergence time for 19 plant species.

The phylogenetic tree was constructed using single-copy genes. The most recent common ancestor (MRCA) was estimated to possess 9205 gene families.

Green numbers indicate expanded gene families, while purple numbers denote contracted gene families.

Gymnosperms are shaded in rose red, while angiosperms are shaded in purple. The dotted line marks the divergence between angiosperms and gymnosperms.

(D). LTR insertion time and frequency of *C. lanceolata*, *Ginkgo biloba*, *Gnetum montanum*, *Picea abies*, and *Pinus taeda*. LTR insertion frequencies in *C. lanceolata* exhibit two distinct peaks, one at approximately 3–7 Mya and another at approximately 14–18 Mya.

Only one peak is evident in the other gymnosperms.

P. taeda (0.72), but significantly lower than that in most other published gymnosperm genomes, such as *T. wallichiana* (5.51), *Ginkgo* (4.26), *W. mirabilis* (3.87), and *Gnetum* (2.07).[42] Solo-LTRs are thought to arise through excision-based DNA recombination, including between adjacent LTRs of the same element, leading to their removal and genome downsizing.[43] Compared with *Ginkgo*, *Gnetum*, *P. abies,* and *P. taeda*, *C. lanceolata* has an extra burst of LTR insertion at approximately 3 Mya (Figure 1D), which may induce the extra expansion of the genome, and relatively low LTR clearance efficiency in *C. lanceolata* leads to the retention of more insertion sequences. Similarly, lower clearance efficiency in *P. abies* and *P. taeda* allows them to have larger genomes even if they do not experience additional LTR mass insertion events. However, whether this low LTR clearance mechanism is common in Cupressaceae remains to be analyzed.

Longer introns, often found in species with larger genomes,[44] such as *Ginkgo* and various conifers, and other sequenced Cupressaceae species, are thought to arise from stable LTR insertions and subsequent amplification.[23,36,45] By analyzing the LTR insertion time, we found that the LTR insertion history in *C. lanceolata* has two major waves: an initial burst peaking between 18 and 14 Mya, shared with species such as *P. abies*, *P. taeda*, *G. montanum*, and *Ginkgo*. After this burst, *C. lanceolata* has a unique secondary wave peaking at 3 Mya (Figures 1C and 1D). This second wave suggests a recent accumulation of LTRs in *C. lanceolata*, not observed in other gymnosperms. For large gymnosperm genomes that commonly lack effective mechanisms to eliminate LTRs,[18,23] the persistent accumulation of LTRs in the *C. lanceolata* genome likely underlies its large genome size. Together, the proliferation of LTR-RTs and elongation of introns contribute to the genome expansion of *C. lanceolata*.

Phylogenetic position within extant lineages of gymnosperms

The present-day gymnosperms represent mere relics of their former diversity, presenting a major challenge in reconstructing evolutionary relationships among extant lineages.[46] Utilizing single-copy gene families derived from comparisons across 19 species, including nine gymnosperms, five angiosperms, two ferns, one lycophyte, and two outgroup species (Figures S7A and S7B), we inferred the species phylogeny using concatenated alignments and ASTRAL trees with both nucleotide and amino acid sequences. Among phylogenetic trees (Figures S7A and S7B), the concatenated tree based on amino acid sequences strongly supported *G. biloba* and *Cycas panzhihuaensis* clustering together and being sister to all other extant gymnosperms, while *T. wallichiana* and *C. lanceolata* formed a sister group of Gnetales (*W. mirabilis* and *G. montanum*)–Pinaceae (*A. alba*, *P. abies*, and *P. taeda*) (Figure S7A). Conversely, the phylogenetic trees constructed by the other three methods supported the hypothesis that Gnetales is a sister group of all other extant gymnosperms, with *G. biloba*-*C. panzhihuaensis* forming a clade sister to conifers (*C. lanceolata*, *T. wallichiana*, and Pinaceae) (Figure S7B). The differences observed between the concatenated trees based on amino acid and the other three phylogenetic trees arise from variations in character state information when using concatenated data, specifically model specification and differences in parallelism/inversion in protein sequences compared to nucleotide coding sequences, which may be affected by substitution saturation.

Similar to the angiosperm branches in the inferred phylogeny, the branch lengths of *W. mirabilis* and *G. montanum,* constructed based on nucleotide sequences, were significantly longer compared to other gymnosperms (Figure S7B). Consequently, the phylogenetic location of Gnetales as the first diverged gymnosperm branch might be influenced by the long-branch attraction (LBA) phenomenon. Strategies to mitigate LBA artifacts include excluding long-branch taxa and fast-evolving third codon positions, as well as employing inference methods less sensitive to LBA.[47] To this end, the concatenated and ASTRAL trees were constructed by selecting the first two codon positions, while the Bayesian phylogenetic tree was constructed using PhyloBayes with the “cat model,” an effective amino acid substitution model for alleviating LBA artifacts. In support of the LBA hypothesis, the results from the Bayesian and concatenated tree approaches favored *G. biloba*-*C. panzhihuaensis* as sisters to the remaining extant gymnosperms, whereas the ASTRAL tree clustered *G. biloba*-*C. panzhihuaensis* with conifers, including *C. lanceolata* (Figures S7A and S7B).

We further expanded the taxonomy, sampling the phylogenetic analysis to 29 gymnosperms by using 25 transcriptomes and four genomes (Figures S7C and S7D). The inferred phylogenetic trees based on the concatenated alignments of either nucleotide or amino acid sequences supported the first view mentioned above (Figure S7A). In summary, based on the outcomes of various phylogenetic analyses and recent researches,[1] we tend to place *G. biloba* and *C. panzhihuaensis* as the sister group of conifers. Among conifers, Gnetales-*Welwitschia* and pine clade are sister groups, forming a sister group to the Cupressaceae plants such as *C. lanceolata*, *C. harringtonia*, *S. verticillata,* and *A. cunninghamii*.

Spermatophyte-wide whole-genome duplication

Distribution of synonymous substitutions per synonymous site (K_S) for paralogous *C. lanceolata* genes revealed no clear peaks indicative of an ancient whole-genome duplication (WGD) during its evolutionary history (Figure S8). Similarly, the paralogous K_S distributions for *G. montanum* and *T. wallichiana* showed no WGD signatures, whereas both *C. panzhihuaensis* and *G. biloba* exhibited K_S peaks corresponding to a shared WGD event, as previously reported.[32] In *W. mirabilis*, a distinct WGD peak was observed, consistent with a lineage-specific WGD event that occurred after its divergence from *G. montanum*.[34]

The absence of a WGD in *C. lanceolata* is further supported by intragenomic comparisons (Figure S9). When comparing the genome of *C. lanceolata* with five other chromosome-level gymnosperm genomes (*G. biloba*, *C. panzhihuaensis*, *W. mirabilis*, *G. montanum*, and *T. wallichiana*), intergenomic collinearity analyses between *C. lanceolata* and *G. biloba*, *C. panzhihuaensis*, and *T. wallichiana* typically revealed only one orthologous collinear segment in *C. lanceolata*, consistent with the absence of a lineage-specific WGD in this species. For *G. biloba* and *C. panzhihuaensis,* which share a WGD, two orthologous collinear segments would be expected in these species

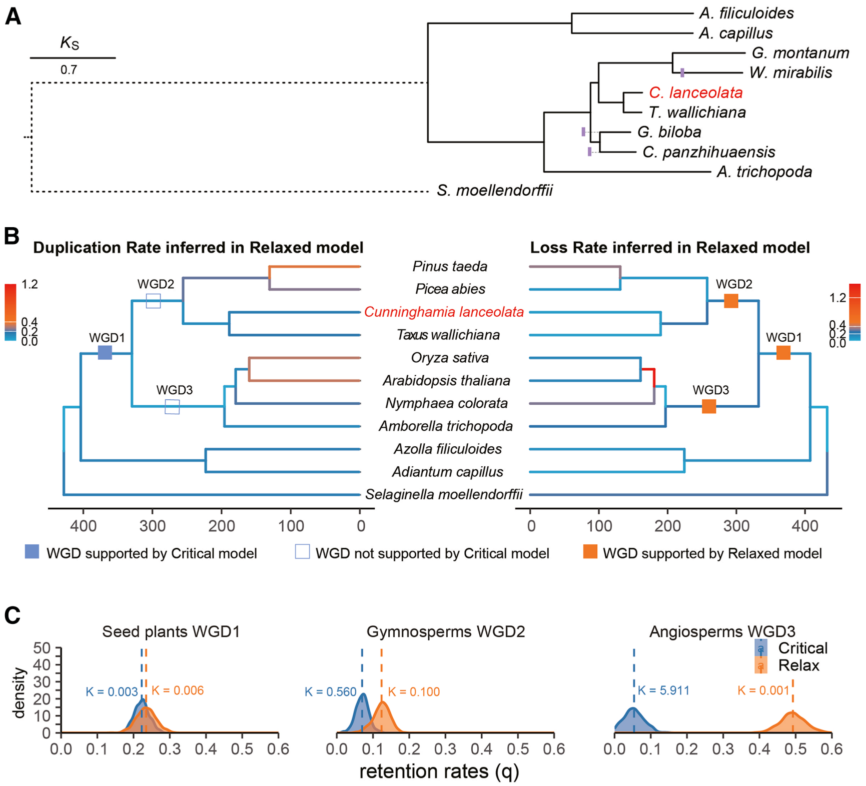

Figure 2. Whole-genome duplication (WGD) events before the early divergence of seed plants

(A). The phylogenetic tree with branch length in K_S units inferred in the phylo-K_S analysis.[46] The three purple squares refer to the three recent WGD events that could be identified in the paralogous K_S distributions for *W. mirabilis*, *G. biloba*, and *C. panzhihuaensis*.

The positions of squares represent the timing s of the WGD events determined by the half of the K_S-peak values inferred in the K_S distributions of all paralogous gene pairs and are mapped on each of the three branches starting from the corresponding tips.

(B). The species tree used in the statistical gene tree – species tree reconciliation with branch lengths representing absolute age obtained from the time tree.[49] Small-scale gene duplication (λ) and loss (μ), and WGD retention (q) were estimated using two models: (1) the critical DL + WGD model ($\lambda = \mu$, rates vary across branches); and (2) the relaxed DL + WGD model ($\lambda \neq \mu$, rates vary across branches).

The inferred relative positions of WGDs are marked on their corresponding branches, with solid blue squares referring to a WGD supported by the critical model ($q > 0$), hollow blue squares referring to WGDs not supported by the critical model ($q \approx 0$), and solid orange squares referring to WGDs supported by the relaxed model ($q > 0$). Duplication and loss rates inferred from the relaxed model were mapped on each branch with a continuous color scale on the left and right tree, respectively.

(C). The posterior distribution of the WGD retention rates(q) drawn from the MCMC sampling with the critical model (in blue) and the relaxed model (in orange) in the WHALE analyses.

The mean of the retention rates is marked by dotted lines, and the Bayes factors (K) are indicated on each distribution.

corresponding to one in *C. lanceolata*; however, due to extensive genomic rearrangements following WGD, collinearity is often reduced.[2,32]

Although *W. mirabilis* clearly experienced a lineage-specific WGD after diverging from *G. montanum*,[34] the WGD shared by *G. biloba* and *C. panzhihuaensis* has been hypothesized to represent a gymnosperm-wide WGD.[32] To test this hypothesis, we performed phylo-K_S analysis[48] (Figure 2A), inferring species phylogenies in K_S units to compare WGD peaks with divergence times (**see Methods**). This analysis confirmed a lineage-specific WGD in *W. mirabilis*, whereas the WGD peaks in *G. biloba* and *C. panzhihuaensis* mapped slightly before the divergence of extant gymnosperms. Although this may suggest a gymnosperm-wide WGD predating their diversification, the accuracy of K_S peak identification[48] and the uncertain topology of major gymnosperm lineages limit confident interpretation.

To further evaluate the existence of a gymnosperm-wide WGD and other potential WGDs before and after gymnosperm divergence, we used WHALE for statistical gene tree-species tree reconciliation.[50] To reduce phylogenetic uncertainties, we excluded *G. biloba*, *C. panzhihuaensis*, *W. mirabilis*, and *G. montanum* (Figures 2B, Figure S10), thereby focusing on the branch leading to the four remaining gymnosperms for testing WGD events after the split from angiosperms, as this branch already includes the stem branch to gymnosperms. In the WHALE analyses, hypothetical WGDs were assigned to the following branches: "WGD 1" on the branch leading to seed plants (spermatophyte-wide); "WGD 2" on the branch leading to the sampled angiosperms; and "WGD 3" on the branch leading to the sampled gymnosperms. Subsequently, we estimated small-scale gene duplication (λ) and loss (μ), and WGD retention (q) using two models: (1) the critical branch-specific DL + WGD

model ($\lambda = \mu$, rates vary across branches); and (2) the relaxed branch-specific DL + WGD model ($\lambda \neq \mu$, rates vary across branches)[50,51] (Table S21).

Guided by Bayes factor analysis, although support for angiosperm- (WGD 2) and gymnosperm-wide (WGD 3) WGDs differed between the two models, both models strongly supported a spermatophyte-wide WGD (WGD 1). Our results further suggest that the WGD shared by *G. biloba* and *C. panzhihuaensis* is more likely a lineage-specific event that occurred before their divergence, rather than a genuine gymnosperm-wide event. The discrepancies between model results for angiosperm- and gymnosperm-wide WGDs may stem from sparse taxon sampling or model misspecification. Although the relaxed DL + WGD model is generally considered more biologically realistic,[48] limited sampling may obscure the differences between gene duplication and loss rates, leaving the critical model ($\lambda = \mu$) as a potentially better fit for sparsely sampled data, as observed in other birth-death models with equal birth and death rates for gene family evolution in distantly related species.[52] Future studies with denser genome sampling and improved computational approaches will help resolve these outstanding questions.

Population history of *C. lanceolata*

Existing fossil records of *C. lanceolata* are sparse. However, significant findings have contributed to our understanding of its early history through closely related species. *C. asiatica*, which shows strong morphological similarities to modern *C. lanceolata* including leaf epidermal structures, branches, leaves, and cones, was found in Northeast China, and confirms that the *Cunninghamia* genus could date back to the Early Cretaceous.[53] Another closely related species, *C. protokonishii*, has been identified in Late Cretaceous to Miocene strata across eastern Siberia (Russia), Japan, and East China. *Cunninghamia* appeared later in North America and Europe, primarily in the Early Tertiary.[54] This evidence suggests that *Cunninghamia* originated in East Asia's circum-Pacific region during the Late Jurassic or Early Cretaceous. It then might spread to North America via the Bering Land Bridge[55] and subsequently to Europe through the North Atlantic Land Bridge,[56] shaping a broad distribution across the Northern Hemisphere.[57]

The Quaternary glaciation had a profound impact on plants' distribution.[58,59] As global temperatures dropped, the range of *C. lanceolata* and other relict species, such as ginkgo,[32] contracted significantly southward, with populations persisting only in certain refugia, such as the Sichuan Basin. As the glaciation waned and temperatures gradually rose, *C. lanceolata* began to disperse from these refugia.[60] Fossil evidence from Holocene strata in the Yangtze River, Pearl River, and Min River basins (south of the Qinling Mountains and Huaihe River)[57] indicates that the post-glaciation expansion of *C. lanceolata* did not fully restore its former range, and instead, its current distribution is primarily centered in South China and northern Vietnam.

To further explore the population history of *C. lanceolata*, we collected 128 samples, of which 115 were from natural populations of China and 13 were from Vietnam (see **Methods** and Table S22). Phylogenetic and principal component analyses, based on 3,419,532 high-quality single-nucleotide polymorphisms (SNPs) (Table S23) generated by the specific locus-amplified fragment (SLAF) method, categorized these 128 samples into three major clades: SCD clade, Middle clade, and Eastern clade (Figures 3A and 3B).

The SCD clade, located within the Sichuan Basin, identified by the ADMIXTURE and paleo-distribution reconstruction analysis (Figures 3C and 3D), exhibited a unique genetic structure, indicating an ancient and isolated lineage that was effectively protected from glacial impacts by this refugium.[61] However, post-glacial migration from this population appears to have been limited, implying that other populations were more responsible for shaping the current distribution of *C. lanceolata.* While the SCD clade may not have directly contributed to the species' modern distribution, gene flow between SCD and neighboring populations remains possible. The overlap of the SCD and SXA populations in the paleo-distribution analysis models (Figure 3D), with the SXA population distributed in the northeastern mountains of the Sichuan Basin (Figure 3E), further supports the potential for gene flow. However, more evidence is needed to confirm inter-population exchange and genetic variation within the refugium. Additionally, other refugia with more favorable conditions might also facilitate their spread.[62]

The Middle clade included the HNX and GZH populations. Interestingly, despite geographic proximity, the two populations show relatively large genetic differences (Figures 3C and E). This divergence could be attributed to the glacial-interglacial cycles that caused repeated range expansion and contraction.[63] The Eastern clade was genetically closer to the GZH population (Figure 3E), probably because the Xuefeng and Nanling Mountains hindered the spread of the HNX population. In contrast, the GZH may have migrated across the plains near the Pearl River to eastern China (Figure 3D), as supported by the discovery of Holocene fossils and buried wood fossils in the Pearl River basin.[62] Unfortunately, we were unable to collect suitable samples from this region, which limits a more precise assessment, and future research is needed to address this gap.

In the Eastern clade, the FJM population appears notably ancient, suggesting a potential role as a post-glacial dispersal hub. The neighbor-joining (NJ) tree illustrates that the YNN, TWB, and SXA populations cluster with FJM, whereas JXJ, JXW, and JSZ populations form another subclade (Figure 3A). This pattern implies that the FJM population initially spread along the southeastern coastline, establishing a coastal subclade, followed by northward dispersal to form an inland subclade, consistent with south-to-north post-glacial warming. However, the clustering of SXA population clusters within this clade with TWB and YNN, and its geographic location, suggest that it may be more appropriately placed within the Middle clade. Additional sampling would help clarify the evolutionary relationships among these populations. Furthermore, we investigated the phylogenetic evolutionary status of the *C. lanceolata* assembly of Shirasawa et al., and revealed that it exhibits a close genetic relationship with the TWB and YNN populations (Figure S11). This finding suggests that *C. lanceolata* in Japan comes from China with a dispersal route via the island of Taiwan.

Formation of astringent seeds and adaptability to aluminum-rich soils

Unlike angiosperms, gymnosperms lack double fertilization. Consequently, the tissue surrounding the gymnospermous

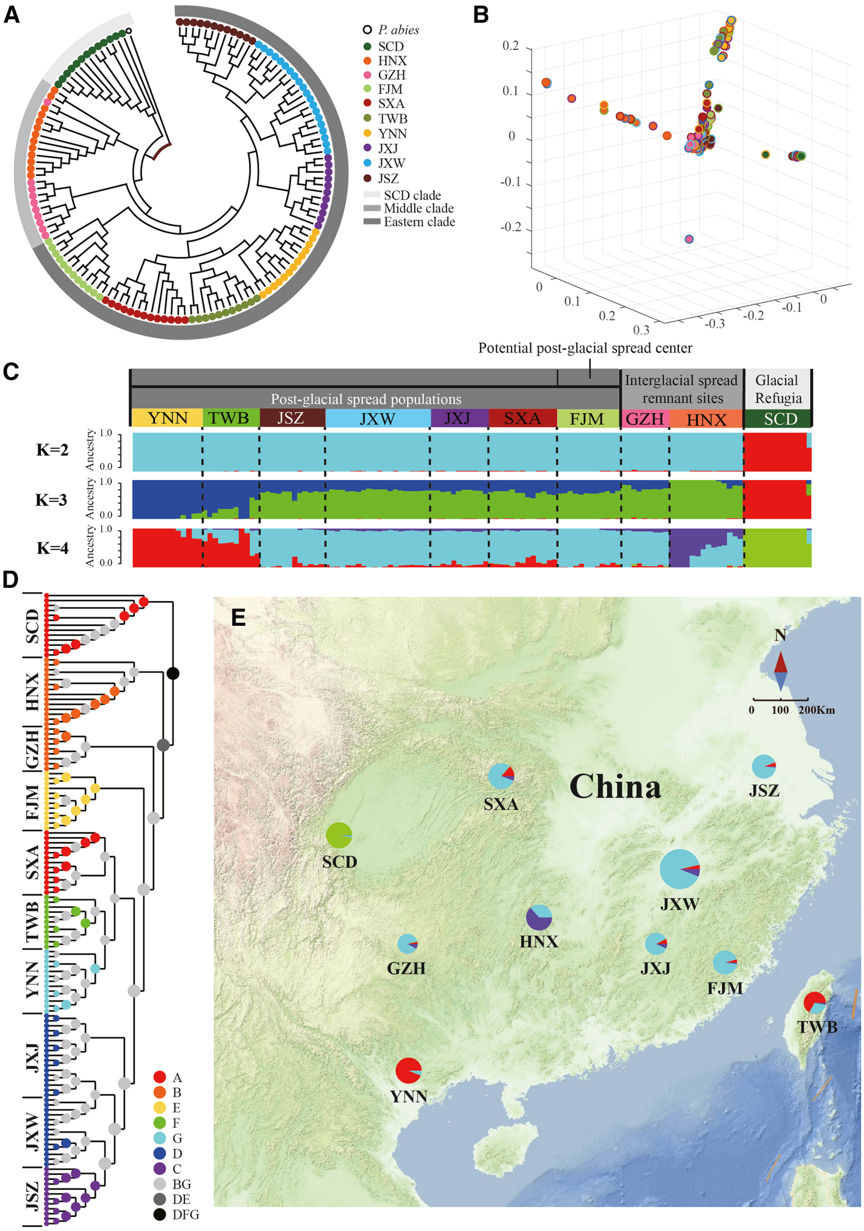

Figure 3. Phylogeography and population history of *Cunninghamia lanceolata*

(A) Maximum likelihood phylogenetic tree of *C. lanceolata* populations constructed based on single-nucleotide polymorphism (SNP) loci.

(B). Principal component analysis (PCA) of all samples.

(C). Population structure of *C. lanceolata*.

(D). Paleo-distribution reconstruction of all samples.

(E). Map illustrating the locations of sampled *C. lanceolata* populations.

embryo is not the true endosperm but a relatively simple structure called the megagametophyte.[64] However, in conifer species, the megagametophytes can serve as nutrient storage organ, although not as extensively as the endosperm.[65] A unique abortive phenomenon occurs in *C. lanceolata*, in which the nutrients in the megagametophyte are replaced by astringent-tasting secondary metabolites, resulting in abortive seeds referred to as "astringent seeds."[66,67] This presents a challenge in distinguishing abortive seeds from viable seeds through simple methods such as visual observation or buoyancy testing.

For further investigation, we dissected and compared the morphological differences between the astringent and normal viable seeds (Method S1). Our findings revealed that embryo abortion typically initiates approximately 95–105 days after pollination (Figure 4A). During this stage, the nucellus undergoes partial decomposition, leading to the formation of a cavity between

the embryo and the nucellus. However, these conditions are transient and do not persist until seed maturity. Instead, metabolites accumulate in the original location of the nucellus cells, gradually filling the cavity and giving the impression of seed growth (Figure 4B). By the time seeds reach maturity, it becomes impossible to distinguish between the two types based solely on appearance. Unfortunately, in *C. lanceolata* seed plantations, the proportion of astringent seeds often exceeds 30%,[68] significantly impeding the selection of high-quality seeds.

Subsequently, we delved into the primary components of secondary metabolites in astringent seeds and their molecular mechanisms of synthesis. Metabolome analysis revealed that the megagametophytes primarily contained flavonoids and their derivatives, with a significant increase in the content of flavonoid metabolites observed from 105 days after pollination (Method S2 and Table S24). Among the 142 genes associated with flavonoid biosynthesis identified in the *C. lanceolata* genome, a considerable proportion were found duplicated (Table S25). Notable examples include flavanone-3-hydroxylase (*F3H*), flavonoid 3′-hydroxylase (*F3′H*), flavonoid 3′,5′-hydroxylase (*F3′5′H*), and flavonol synthase (*FLS*) (Figures S12 and S13). Additionally, 29 genes showed upregulation during the mid to late stages (Figure S14), corresponding with the observed sequence of morphological changes. We further identified 771 embryo-defective genes (*EMBs*)[69] (Table S26), 109 of which exhibited significant differential expression between astringent and germinated seeds (Figure S15). Abnormal levels of endogenous hormones associated with embryonic development and changes in related gene expression were also observed. These phenotypic, metabolic, and transcriptomic findings suggest that the formation of astringent seeds may result from two interconnected processes. First, the altered accumulation and expression of endogenous hormones, such as brassinosteroids (BR) and abscisic acid (ABA), alongside genes involved in embryonic development, disrupt normal embryo formation. Second, embryo degeneration triggers cell disintegration, causing signaling molecules, such as α-linoleic acid, to activate the jasmonic acid (JA) signaling pathway. Additionally, the potential accumulation of reactive oxygen species (ROS)[70] within the tissue further stimulates flavonoid biosynthesis, which, through antioxidant responses,[71–73] leads to the gradual accumulation of astringent compounds (Figure 4C). We then randomly selected four relevant genes for transgenic verification in *A. thaliana* (method S3). The results showed that, compared to the wild type (WT), the T1 generation seeds exhibited a darker color following the introduction of *ClLAR* and *ClANR*, two genes involved in flavonoid biosynthesis. In contrast, the introduction of *ClDET2* and *ClBZR1*, genes associated with BR synthesis and signaling, led to an increase in the number of seeds with abnormal sizes (Figure 4D). Furthermore, the transgenic lines exhibited a significant reduction in seed germination rates compared to WT (Figure 4E). We identified that all four genes regulating the astringent seed trait are present in the genome reported by Shirasawa et al. Notably, the homologous gene of Cl08429.1 (Cl08429.1-homo) harbors a G-to-T substitution at the 667th nucleotide of the CDS region, which introduces a premature stop codon (Table S27; Figure S16). It suggests that this gene in the genome assembly by Shirasawa et al. has been pseudogenized.

We find it perplexing that a plant such as *C. lanceolata*, which has undergone millions of years of evolution, would develop the trait of producing astringent seeds. On the surface, it appears to invest energy into "growing" seeds without providing any immediate benefits. Therefore, we scrutinized its habitat and proposed a potential underlying hypothesis. *C. lanceolata* is primarily distributed in southern China, where the predominant soil type is acidic red soil (Figure S17). Soil acidification leads to the release of significant amounts of active aluminum, resulting in a higher aluminum content in the soil, which adversely affects plant root growth.[74–76]

Mu et al.[77] observed that aluminum stress significantly increased active Al^{3+} ion content, malondialdehyde (MDA) levels, and superoxide dismutase (SOD) activity in *C. lanceolata* seedlings, while inhibiting root elongation. However, the introduction of a low concentration of astringent seed water extract alleviated these effects, suggesting that astringent seeds may protect germinating seeds by releasing secondary metabolites that mitigate aluminum toxicity. This indicates that astringent seeds represent an evolutionary adaptation to high-aluminum environments. Certain plants have the ability to sequester activated aluminum ions within the vacuoles of mesophyll cells as aluminum ion-organic acid complexes, thus reducing aluminum toxicity to roots.[78,79] Unlike most plants, whose leaves promptly shed upon wilting, the leaves of *C. lanceolata* remain on branches for extended periods. These leaves serve as reservoirs for absorbed reactive aluminum, impeding the flux and circulation of reactive aluminum between plants and soil.[80] Consequently, this further reduces the detrimental effects of reactive aluminum in the soil (Method S4 and Figure S18). In summary, the development of astringent seeds and the persistence of dead branches in *C. lanceolata* represent specific adaptations to its environment, enabling the plant to thrive in high-aluminum environments.

Evolution of reproductive organs

Gymnosperms possess both male and female reproductive organs, typically in the form of cones, whereas angiosperms usually feature reproductive organs enclosed within flowers, including sepals, petals, stamens (male reproductive organs), and carpels (female reproductive organs).[81] Based on studies of homozygous mutants, the ABC model has been proposed to elucidate how distinct floral organs acquire their specific "identities" during development.[82] Subsequently, the ABC model was expanded into the "ABCDE model," wherein class A + E genes specify sepals, A + B + E genes specify petals, B + C + E genes specify stamens, C + E genes specify carpels, and C + D + E genes specify ovules.[83–87] Among the five classes of floral homeotic genes, classes A and E originated from the duplication of a common ancestor shared by gymnosperms and angiosperms (A), whereas the C and D functions of angiosperms were derived from a combined C/D function provided by *AG-like* genes (C) in extant gymnosperms and stem-group seed plants.[88–92] Considering these insights, the overarching ABCDE model was simplified to the (A)B(C) model applicable to all seed plants.[88,93]

MADS-box transcription factors regulate floral organ morphogenesis and flowering time, especially within the *AP1/FUL* (class

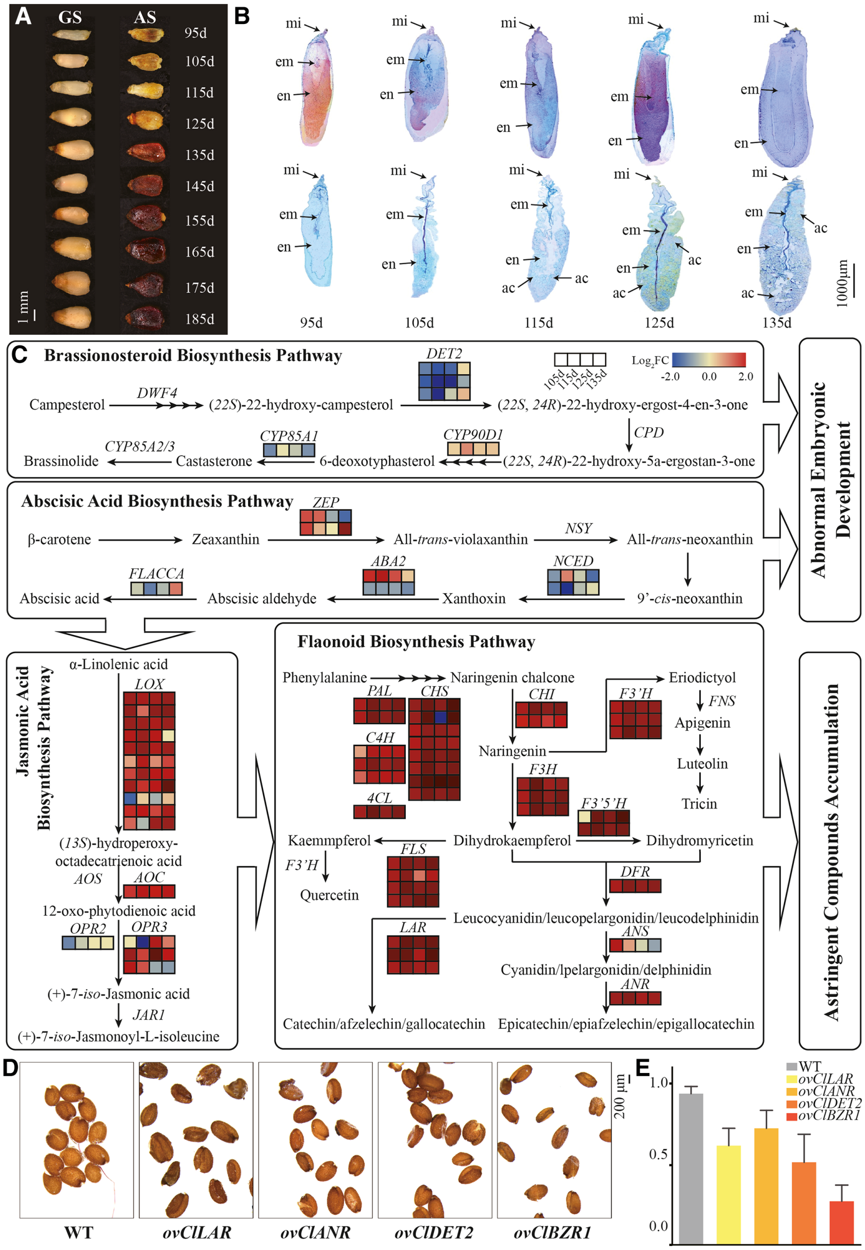

Figure 4. Astringent seed (AS) formation

(A) Phenotypic characteristics depict the development of germinating seeds (GS) and AS.

The marked times represent the corresponding days after pollination. Length of the scale bar represents 1 mm.

(B) Microscopic observation of AS and GS at different development stages.

Key: mi, micropyle; em, embryo; ho, homozygous female gametophyte; ac, astringent compound. Microscopic scanning revealed that GS displays a complete embryo structure during development, whereas exhibits aborted embryos, broken homozygous female gametophytes, and accumulated secondary metabolites. The marked times represent the corresponding days after pollination. Length of the scale bar represents 1000 μm.

(C) Proposed putative mechanism of astringent seed formation. The formation of astringent seeds is closely linked to the disruption of key hormonal signaling pathways involved in embryonic development. Abnormal expression of genes in the brassinosteroid (BR) and abscisic acid (ABA) biosynthetic pathways is observed during astringent seed formation.

As the embryo degenerates, tissue cells begin to disintegrate, triggering stress response mechanisms. This includes the activation of the jasmonic acid (JA) pathway and the flavonoid biosynthesis pathway, which collectively result in the accumulation of astringent compounds.

(D) Phenotypic differences in T1 generation transgenic *A. thaliana* seeds.

(legend continued on next page)

CellPress
OPEN ACCESS

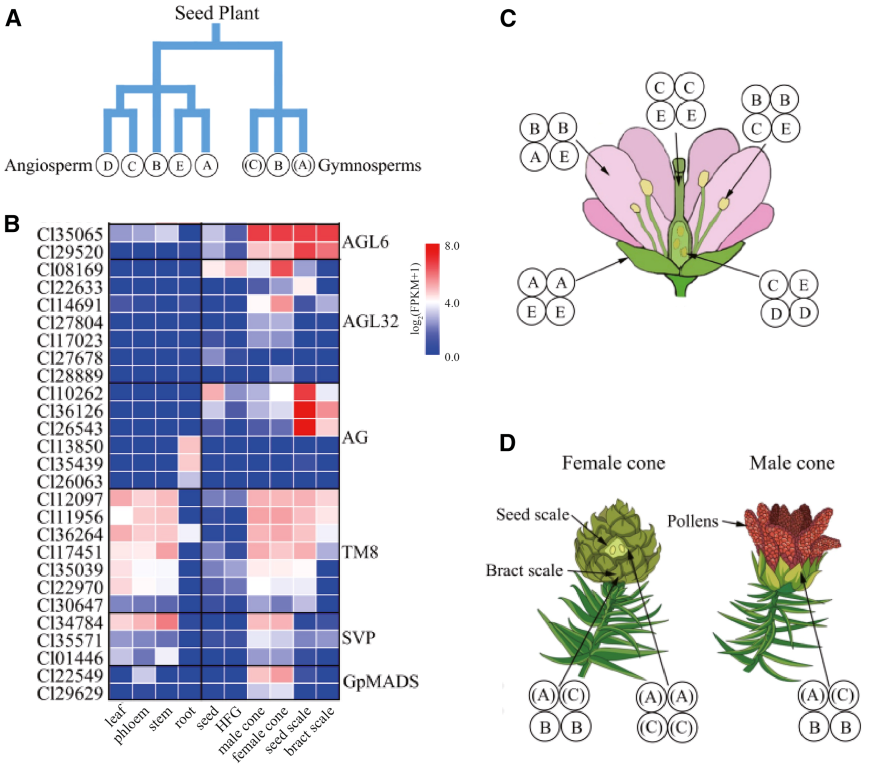

Figure 5. Analysis of reproductive organ genes of *Cunninghamia lanceolata*

(A). Homologous genes regulating reproductive organ development in angiosperms and gymnosperms.

The (A) class genes in the gymnosperm reproductive organ development model (A)B(C) are homologous to the A and E class genes in the angiosperm flower development ABCE model.

The (C) class genes are homologous to the C and D class genes (Figure S8).

(B). Expression profile of MIKCc-type genes. HFG, homozygous female gametophyte.

(C) Angiosperm flower model.

(D) Regulation model of female (left) and male (right) organs in *C. lanceolata*.

A), *AP3/PI/AGL32* (class B), *AG/STK* (class *C/D*), and *SEP* (class *E*) subfamilies.[94,95] To elucidate the phylogenetic relationships among floral homeotic genes, we identified MADS-box genes in the *C. lanceolata* genome and constructed a phylogenetic tree based on the MADS-box genes from four gymnosperm and 10 angiosperm species (Figure S19). A total of 50 MADS-box genes were identified, including 32 type II genes (27 *MIKCc-type* genes and five *MIKC*-type* genes) and 18 type I genes (11 *Mα* genes and seven *Mγ* genes) (Table S28). The *MIKCc-type* genes were classified as *AGL6* (two members), *AGL32* (seven members), *AG* (six members), *GMADS* (two members), *TM8* (six members), *SOC1* (one member), and *SVP* (three members) (Table S28). Phylogenetic reconstruction of the *MIKc-type* MADS-box gene family revealed distinct clades corresponding to classes A(*AP1/FUL*), B(*AP3/PI/AGL32*), C + D (*AG/STK*), and E (*SEP*) of floral homeotic genes (Figure S19). Notably, the A(*AP1/FUL*), E(*SEP*), and *AGL6* subfamilies shared a common ancestor, while the *AP1-like* and *SEP-like* genes were absent in gymnosperms. Furthermore, the *AGL6* subfamily in gymnosperms exhibited division into two clades, one unique to gymnosperms and the other clustering with angiosperms. This suggests the presence of orthologues of floral homeotic class A and E genes in extant gymnosperms and angiosperms, with *AGL6*-like genes potentially playing crucial roles in gymnosperm reproductive organs. Experimental evidence across different species has confirmed that *AGL6-like* genes exert an E function.[96–98] Additionally, the *AG-like* (class C) and *STK-like* (class D) genes clustered together in an evolutionary branch, with gymnosperm *AG-like* genes positioned at the base (Figure S19), indicating a potential role in specifying reproductive organ identity in gymnosperms. Taken together, most floral homeotic genes in extant gymnosperms exhibit close relationships or are putative

Compared to the wild type (WT), seeds introduced and over-expressed with the *ClLAR* and *ClANR* genes displayed a darker color, indicating an increased accumulation of flavonoids.

Seeds introduced with the *ClDET2* and *ClBZR1* genes exhibited a higher proportion of seeds with abnormal sizes, suggesting possible disruptions in embryo development. Length of the scale bar represents 200 μm.

(E) Seed germination rates in each transgenic line. The error bars represent the standard deviation (SD).

orthologues, raising questions about the roles of genes, such as class B (*AGL32-like* genes), class C + D (*AG-like* genes), and class A + E (*AGL6-like* genes), in the development of gymnosperm reproductive organs (Figure 5A).

C. lanceolata features conical male cones clustered at the apex of its branches, while its female cones typically serve as the female reproductive unit, with multiple spirally arranged fertile seed scales that are axillary to non-fertile bract scales. Subsequently, we analyzed the expression patterns of floral homeotic genes in the reproductive organs of *C. lanceolata* to elucidate their role in determining reproductive organ characteristics. Previous studies have indicated that *AG-like* genes play a crucial role in distinguishing reproductive organs from non-reproductive organs, while the differential expression of *AGL32-like* genes is associated with the differentiation between male and female reproductive organs.[82,99,100] Our expression analyses (Figure 5B) revealed two distinct expression patterns for *AG-like* (C class) genes in *C. lanceolata*. Specifically, the *AG-like* genes *Cl10262*, *Cl36126*, and *Cl26543* exhibited expression in reproductive organs, with their highest levels observed in the seed scales, whereas *Cl13850*, *Cl35439*, and *Cl26063* were solely expressed in the roots. Regarding *AGL32-like* genes, all except Cl27678 exhibited expression in reproductive organs, with peak expression in male cones and notable *Cl14691* expression in non-fertile bract scales. The expression patterns of class B and C gene orthologues in *C. lanceolata* closely resemble those observed in angiosperm class B and C genes (Figures 5C and 5D). Specifically, class C genes (*AG-like* genes *Cl10262*, *Cl36126*, and *Cl26543*) are expressed in both male and female reproductive organs, especially in the seed scales, whereas class B genes (*AGL32-like* gene *Cl14691*) are mainly expressed in male reproductive organs and bract scales. In summary, classes B+(C) specify male cones and bract scales, while class C specifies seed scales.

In line with previous findings,[96,101] we identified two distinct expression patterns for *AGL6-like* genes. Specifically, *Cl29520* was exclusively expressed in reproductive organs (male cones, female cones, seed scales, and bract scales), whereas *Cl35065* was widely expressed in both reproductive and vegetative organs (Figure 5B). The expression of the *AGL6-like* gene *Cl29520* overlapped with that of the *AGL32-like* (*Cl14691*) and *AG-like* (*Cl10262*, *Cl36126*, and *Cl26543*) genes, suggesting its involvement not only in the differentiation of reproductive meristematic tissues but also in specifying reproductive organ identity alongside *AGL32-like* and *AG-like* genes. The proposed gymnosperm (A)B(C) model suggests that the B-(A)-(C) genes determine male cones, whereas the (A)-(C) genes specify female cone organs.[88] Combining the results of the phylogenetic and expression analyses, we further refined the gymnosperm (A)B(C) model: B-(A)-(C) specifies bract scales and male cones, while (A)–(C) specifies seed scales (Figure 5C and 5D).

DISCUSSION

Gymnosperms, which appeared approximately 360 Mya. However, the present-day gymnosperms represent only a fraction of their diversity of the past, posing a significant challenge in reconstructing the evolutionary relationship among extant lineages (such as Gnetales, Cycads, Ginkgo, and conifers), especially because of limited genomic data on the Cupressaceae family of conifers. This study explores the chromosome-level genomic profile of the Cupressaceae species *C. lanceolata*, prevalent in acidic, aluminum-rich soils of southern China. The final assembled genome of *C. lanceolata* stood at 11.24 Gb, with repetitive sequences accounting for 10.38 Gb, predominantly comprised of LTR elements. The larger genome size of *C. lanceolata* can be attributed to longer introns, specific LTR accumulation, and the lack of an efficient LTR elimination mechanism. Phylogenomic analyses confirmed the clustering of *Ginkgo* and *Cycads* as a sister group to all other extant gymnosperms. The Taxaceae (*T. wallichiana*)–Cupressaceae (*C. lanceolata*) clade forms a sister group to Gnetales (*W. mirabilis* and *G. montanum*)–Pinaceae (*A. alba*, *P. abies*, and *P. taeda*). The WGD analysis showed that seed plant ancestors diverged into angiosperms and gymnosperms following a seed plant-specific WGD event. Moreover, the ancestor of the extant gymnosperms underwent a gymnosperm-specific WGD event. No evidence supports the occurrence of an ancient polyploidization Event in the lineage of *C. lanceolata*. Our study, encompassing 128 samples of *C. lanceolata* from 10 populations in China and Vietnam, identified a potential refugium in the Sichuan Basin (SCD population). The FJM population likely served as the origin of contemporary *C. lanceolata* populations. Furthermore, we found that certain embryos of *C. lanceolata* begin to abort 95–105 days after pollination, being replaced by flavonoids and forming astringent seeds (non-fertile) that are visually indistinguishable from healthy seeds. By constructing a phylogenetic tree from the MADS-box genes of 14 seed plants and analyzing the expression profiles of the MADS-box genes in *C. lanceolata* nutrient and reproductive organs, we employed the (A)B(C) model to elucidate the development of reproductive organs in *C. lanceolata*. This study provides a valuable genome sequence and contributes to the understanding of gymnosperm evolution.

Limitations of this study

In the presented article, we report the genome of *C. lanceolata*, a key species in the Cupressaceae family, and expand on the evolution of gymnosperms. While we believe this work is significant, it has several limitations. The techniques used, including sequencing, assembly, and annotation, are somewhat outdated, as the project began earlier. With current technological advancements, we believe the quality of the assembly could have been greatly improved. Secondly, we did not explore certain genomic aspects of *C. lanceolata*, such as methylation sites, which are essential for understanding traits such as wood formation, growth habits, reproductive development, and stress resistance. Additionally, our sampling has limitations. For instance, our population structure analysis did not include samples from regions such as Guangdong and Guangxi. Although we surveyed these areas, the strong human influence on the cultivation and distribution of *C. lanceolata* seeds has complicated our understanding of its natural distribution. Therefore, we only collected samples from primary forests, aiming for those older than 50 years. Had we been more persistent in acquiring samples from these regions, our data might have been more comprehensive. Of course, there are

other limitations beyond those mentioned here, and we hope future research will address these gaps.

RESOURCE AVAILABILITY

Lead contact

Further information and requests for resources should be directed to and will be fulfilled by the Lead Contact, Zhong-Jian Liu (zjliu@fafu.edu.cn).

Materials availability

This study did not generate new unique reagents.

Data and code availability

- The data supporting this work are available within the paper and supplementary files.
- PacBio whole-genome sequencing data, Illumina data, and genome assembly sequences have been deposited to the NCBI Sequence Read Archive (SRA) as Bioproject PRJNA668674.
- This paper does not report original code.
- Any additional information required to reanalyze the data reported in this paper is available from the lead contact upon request.

ACKNOWLEDGMENTS

We acknowledge the support received through the Innovative Research and Constructive Platform Funds of Fujian Agriculture and Forestry University (118-612014032 and KLE18010A), awarded to S-Z. L.; the National Key Research and Development Program of China (2021YFD2201302), and National Natural Science Foundation of China (no. 32572020), awarded to Y. C; and Forestry Peak Discioline Construction Project of Fujian Agriculture and Forestry University (72202200205), awarded to Z.-J. L.; Y.V.d.P. acknowledges funding from the European Research Council (ERC) under the European Union's Horizon 2020 research and innovation program (no. 833522) and from Ghent University (Methusalem funding, BOF.MET.2021.0005.01) ; Z.L. acknowledges funding from the Junior Research Project of FWO (G0ADO25N) and the Special Research Grant from Ghent University (BOF.BAF.2024.0889.01).

AUTHOR CONTRIBUTIONS

S.-Z.L. and Z.-J.L. managed the project; , Y.V.d.P., R.M., H.-K.Z., S.-Z.L., Y.C., W.-H.S., C.W., and Z.L. planned and coordinated the project; S.-Z.L., Z.-J.L., Y.C., W.-H.S., C.W., and Z.L. wrote the manuscript; W.-H.S., Y.C., C.W., Y.-C.X., L.Y., Y.Z., M.-M.L., W.-C.T. and Y.-C.X. collected and sequenced the plant material; H.-K.Z., Q.-G. Z., W.-H.S., C.W., L.X., D.-K.L., D.-Q.C., and L.N. assembled and annotated the genome; S.-Z.L., Z.L., H.-C.C., J.-Y.W., Z.-W.W., and Z.-W.L., performed gene family clustering and comparative phylogenomics; Y.C., D.-Y.Z., X.Y., D.-K.L., G.-Z.C., J.H., M.-Z.H., X.Z., W.-Y.Z., F.-L.W., Y. L., Q. Z., and X. Z., executed transcriptome sequencing and analysis; W.-H.S., W.-C.T., Y.-C.X., S.-S.X., A.-Q.L., Y.-Q.L., S.-N.R., and B.L. conducted the evolution of reproductive organs analysis; C.W., X.-Q., M., F.-P., Z., Y., L.Y., Y.Z., M.-M.L., J.-J.Z., Z.-M.H., P.-F.W., and K.-M.L., conducted the formation of astringent seeds analysis. S.-Z.L., Z.-J.L., X.-Y.C., Y.C, C.-M.J., S.-R.L., S.-B.L., Y.-Q.Y., Z.-H.M., G.-C.D., G.-Q.C., S.X., and J.Z., conducted the evolution of reproductive organs analysis. All authors read and approved the manuscript.

DECLARATION OF INTERESTS

The authors declare no competing interests.

STAR★METHODS

Detailed methods are provided in the online version of this paper and include the following:

- KEY RESOURCES TABLE
- EXPERIMENTAL MODEL AND STUDY PARTICIPANT DETAILS
  - Plant materials and growing conditions
- METHOD DETAILS
  - DNA preparation and sequencing
  - Genome size and heterozygosity estimation
  - Genome assembly
  - Hi-C library construction and chromosome assembly
  - Gene prediction and annotation
  - Identification of non-coding RNA, pseudogene, and repetitive sequences
  - Building gene families with OrthoMCL
  - Phylogenetic reconstruction
  - Estimation of divergence time
  - Gene family expansion and contraction
  - Analysis of whole-genome duplications (WGDs) and intra- and intergenomic comparisons
  - Transcriptomic data and analysis
  - Demographic history analysis
  - MADS-box gene family analysis
- QUANTIFICATION AND STATISTICAL ANALYSIS

SUPPLEMENTAL INFORMATION

Supplementary data related to this article can be found online at https://doi.org/10.1016/j.celrep.2026.117566.

Received: September 24, 2024
Revised: April 27, 2026
Accepted: May 28, 2026
Published: June 18, 2026

REFERENCES

1. Yang, Y., Ferguson, D.K., Liu, B., Mao, K.S., Gao, L.M., Zhang, S.Z., Wan, T., Rushforth, K., and Zhang, Z.X. (2022). Recent advances on phylogenomics of gymnosperms and a new classification. Plant Divers. *44*, 340–350. https://doi.org/10.1016/j.pld.2022.05.003.
2. Liu, Y., Wang, S., Li, L., Yang, T., Dong, S., Wei, T., Wu, S., Liu, Y., Gong, Y., Feng, X., et al. (2022). The *Cycas* genome and the early evolution of seed plants. Nat. Plants *8*, 389–401. https://doi.org/10.1038/s41477-022-01129-7.
3. Farjon, A. (2010). A Handbook of the World's Conifers, *1* (Leiden, Boston RBG: BRILL), pp. 1–526.
4. Forest, F., Moat, J., Baloch, E., Brummitt, N.A., Bachman, S.P., Ickert-Bond, S., Hollingsworth, P.M., Liston, A., Little, D.P., Mathews, S., et al. (2018). Gymnosperms on the EDGE. Sci. Rep. *8*, 6053. https://doi.org/10.1038/s41598-018-24365-4.
5. Rothwell, G.W., and Scheckler, S.E. (1988). In Origin and Evolution of Gymnosperms, C.B. Beck, ed. (New York: Columbia University Press), pp. 85–134.
6. Govaerts, R., Nic Lughadha, E., Black, N., Turner, R., and Paton, A. (2021). The World Checklist of Vascular Plants, a continuously updated resource for exploring global plant diversity. Sci. Data *8*, 215. https://doi.org/10.1038/s41597-021-00997-6.
7. Savard, L., Li, P., Strauss, S.H., Chase, M.W., Michaud, M., and Bousquet, J. (1994). Chloroplast and nuclear gene sequences indicate late Pennsylvanian time for the last common ancestor of extant seed plants. Proc. Natl. Acad. Sci. USA *91*, 5163–5167. https://doi.org/10.1073/pnas.91.11.5163.
8. Lu, Y., Ran, J.H., Guo, D.M., Yang, Z.Y., and Wang, X.Q. (2014). Phylogeny and divergence times of gymnosperms inferred from single-copy nuclear genes. PLoS One *9*, e107679. https://doi.org/10.1371/journal.pone.0107679.
9. Kusumi, J., Tsumura, Y., Yoshimaru, H., and Tachida, H. (2000). Phylogenetic relationships in Taxodiaceae and Cupressaceae sensu stricto

based on matK gene, chlL gene, trnL-trnF IGS region, and trnL intron sequences. Am. J. Bot. *87*, 1480–1488. https://doi.org/10.2307/2656874.

10. Miura, H., and Kawano, N. (1968). On the distribution of bisflavones in the leaves of Taxodiaceae and Cupressaceae plants. Yakugaku Zasshi *88*, 1459–1462. https://doi.org/10.1248/yakushi1947.88.11_1459.
11. Miller, C.N. (1977). Mesozoic conifers. Bot. Rev. *43*, 217–280. https://doi.org/10.1007/BF02860718.
12. Ariano, R., Panzani, R.C., and Augeri, G. (1997). Double-blind placebo controlled specific immunotherapy with mixed Cupressaceae taxodiaceae pollens in respiratory allergy to Cupressus sempervirens. Allergol. Immunopathol. *25*, 23–29.
13. Mao, K., Milne, R.I., Zhang, L., Peng, Y., Liu, J., Thomas, P., Mill, R.R., and Renner, S.S. (2012). Distribution of living Cupressaceae reflects the breakup of Pangea. Proc. Natl. Acad. Sci. USA *109*, 7793–7798. https://doi.org/10.1073/pnas.1114319109.
14. Wickett, N.J., Mirarab, S., Nguyen, N., Warnow, T., Carpenter, E., Matasci, N., Ayyampalayam, S., Barker, M.S., Burleigh, J.G., Gitzendanner, M.A., et al. (2014). Phylotranscriptomic analysis of the origin and early diversification of land plants. Proc. Natl. Acad. Sci. USA *111*, e4859–e4868. https://doi.org/10.1073/pnas.1323926111.
15. Ruhfel, B.R., Gitzendanner, M.A., Soltis, P.S., Soltis, D.E., and Burleigh, J. (2014). From algae to angiosperms-inferring the phylogeny of green plants (Viridiplantae) from 360 plastid genomes. BMC Evol. Biol. *14*, 23. https://doi.org/10.1186/1471-2148-14-23.
16. Bowe, L.M., Coat, G., and dePamphilis, C.W. (2000). Phylogeny of seed plants based on all three genomic compartments: Extant gymnosperms are monophyletic and Gnetales' closest relatives are conifers. Proc. Natl. Acad. Sci. USA *97*, 4092–4097. https://doi.org/10.1073/pnas.97.8.4092.
17. Hajibabaei, M., Xia, J., and Drouin, G. (2006). Seed plant phylogeny: gnetophytes are derived conifers and a sister group to Pinaceae. Mol. Phylogenet. Evol. *40*, 208–217. https://doi.org/10.1016/j.ympev.2006.03.006.
18. Wang, Y., Tang, H., Debarry, J.D., Tan, X., Li, J., Wang, X., Lee, T.h., Jin, H., Marler, B., Guo, H., et al. (2012). MCScanX: a toolkit for detection and evolutionary analysis of gene synteny and collinearity. Nucleic Acids Res. *40*, e49. https://doi.org/10.1093/nar/gkr1293.
19. Li, Z., De La Torre, A.R., Sterck, L., Cánovas, F.M., Avila, C., Merino, I., Cabezas, J.A., Cervera, M.T., Ingvarsson, P.K., and Van de Peer, Y. (2017). Single-copy genes as molecular markers for phylogenomic studies in seed plants. Genome Biol. Evol. *9*, 1130–1147. https://doi.org/10.1093/gbe/evx070.
20. Morse, A.M., Peterson, D.G., Islam-Faridi, M.N., Smith, K.E., Magbanua, Z., Garcia, S.A., Kubisiak, T.L., Amerson, H.V., Carlson, J.E., Nelson, C.D., and Davis, J.M. (2009). Evolution of genome size and complexity in *Pinus*. PLoS One *4*, e4332. https://doi.org/10.1371/journal.pone.0004332.
21. Mackay, J., Dean, J.F.D., Plomion, C., Peterson, D.G., Cánovas, F.M., Pavy, N., Ingvarsson, P.K., Savolainen, O., Guevara, M.Á., Fluch, S., et al. (2012). Towards decoding the conifer giga-genome. Plant Mol. Biol. *80*, 555–569. https://doi.org/10.1007/s11103-012-9961-7.
22. De La Torre, A.R., Li, Z., Van de Peer, Y., and Ingvarsson, P.K. (2017). Contrasting rates of molecular evolution and patterns of selection among gymnosperms and flowering plants. Mol. Biol. Evol. *34*, 1363–1377. https://doi.org/10.1093/molbev/msx069.
23. Nystedt, B., Street, N.R., Wetterbom, A., Zuccolo, A., Lin, Y.C., Scofield, D.G., Vezzi, F., Delhomme, N., Giacomello, S., Alexeyenko, A., et al. (2013). The Norway spruce genome sequence and conifer genome evolution. Nature *497*, 579–584. https://doi.org/10.1038/nature12211.
24. Birol, I., Raymond, A., Jackman, S.D., Pleasance, S., Coope, R., Taylor, G.A., Yuen, M.M.S., Keeling, C.I., Brand, D., Vandervalk, B.P., et al. (2013). Assembling the 20 Gb white spruce (*Picea glauca*) genome from whole-genome shotgun sequencing data. Bioinformatics *29*, 1492–1497. https://doi.org/10.1093/bioinformatics/btt178.
25. Wegrzyn, J.L., Liechty, J.D., Stevens, K.A., Wu, L.S., Loopstra, C.A., Vasquez-Gross, H.A., Dougherty, W.M., Lin, B.Y., Zieve, J.J., Martínez-García, P.J., et al. (2014). Unique features of the loblolly pine (*Pinus taeda L.*) megagenome revealed through sequence annotation. Genetics *196*, 891–909. https://doi.org/10.1534/genetics.113.159996.
26. Zimin, A., Stevens, K.A., Crepeau, M.W., Holtz-Morris, A., Koriabine, M., Marçais, G., Puiu, D., Roberts, M., Wegrzyn, J.L., De Jong, P.J., et al. (2014). Sequencing and assembly of the 22-gb loblolly pine genome. Genetics *196*, 875–890. https://doi.org/10.1534/genetics.113.159715.
27. Niu, S., Li, J., Bo, W., Yang, W., Zuccolo, A., Giacomello, S., Chen, X., Han, F., Yang, J., Song, Y., et al. (2022). The Chinese pine genome and methylome unveil key features of conifer evolution. Cell *185*, 204–217.e14. https://doi.org/10.1016/j.cell.2021.12.006.
28. Jang, M.J., Cho, H.J., Park, Y.S., Lee, H.,Y., Bae, E.K., Jung, S., Jin, H., Woo, J., Park, E., Kim, S.J., et al. (2024). Haplotype-resolved genome assembly and resequencing analysis provide insights into genome evolution and allelic imbalance in *Pinus densiflora*. Nat. Genet. *56*, 2551–2561. https://doi.org/10.1038/s41588-024-01944-y.
29. Sun, C., Xie, Y.H., Li, Z., Liu, Y., Sun, X., Li, J., Quan, W., Zeng, Q., Van de Peer, Y., and Zhang, S. (2022). The *Larix kaempferi* genome reveals new insights into wood properties. J. Integr. Plant Biol. *64*, 1364–1373. https://doi.org/10.1111/jipb.13265.
30. Xiong, X., Gou, J., Liao, Q., Li, Y., Zhou, Q., Bi, G., Li, C., Du, R., Wang, X., Sun, T., et al. (2021). The Taxus genome provides insights into paclitaxel biosynthesis. Nat. Plants *7*, 1026–1036. https://doi.org/10.1038/s41477-021-00963-5.
31. Zhao, Y.P., Fan, G., Yin, P.P., Sun, S., Li, N., Hong, X., Hu, G., Zhang, H., Zhang, F.M., Han, J.D., et al. (2019). Resequencing 545 ginkgo genomes across the world reveals the evolutionary history of the living fossil. Nat. Commun. *10*, 4201. https://doi.org/10.1038/s41467-019-12133-5.
32. Liu, H., Wang, X., Wang, G., Cui, P., Wu, S., Ai, C., Hu, N., Li, A., He, B., Shao, X., et al. (2021). The nearly complete genome of *Ginkgo biloba* illuminates gymnosperm evolution. Nat. Plants *7*, 748–756. https://doi.org/10.1038/s41477-021-00933-x.
33. Wan, T., Liu, Z.M., Li, L.F., Leitch, A.R., Leitch, I.J., Lohaus, R., Liu, Z.J., Xin, H.P., Gong, Y.B., Liu, Y., et al. (2018). A genome for gnetophytes and early evolution of seed plants. Nat. Plants *4*, 82–89. https://doi.org/10.1038/s41477-017-0097-2.
34. Wan, T., Liu, Z., Leitch, I.J., Xin, H., Maggs-Kölling, G., Gong, Y., Li, Z., Marais, E., Liao, Y., Dai, C., et al. (2021). The *Welwitschia* genome reveals a unique biology underpinning extreme longevity in deserts. Nat. Commun. *12*, 4247. https://doi.org/10.1038/s41467-021-24528-4.
35. Fu, F., Song, C., Wen, C., Yang, L., Guo, Y., Yang, X., Shu, Z., Li, X., Feng, Y., Liu, B., et al. (2023). The *Metasequoia* genome and evolutionary relationship among redwoods. Plant Commun. *4*, 100643. https://doi.org/10.1016/j.xplc.2023.100643.
36. Rong, J., Zheng, Y., Zhang, Z., Zhang, J., Gu, Y., Hua, T., Zhao, M., Fan, L., Deng, Z., Pan, Y., et al. (2024). De nove whole-genome assembly of the 10-gigabase *Fokienia hodginsii* genome to reveal differential epigenetic events between callus and xylem. Adv. Sci. *11*, 2402644. https://doi.org/10.1002/advs.202402644.
37. Shirasawa, K., Mishima, K., Hirakawa, H., Hirao, T., Tsubomura, M., Nagano, S., Iki, T., Isobe, S., and Takahashi, M. (2024). Haplotype-resolved de novo genome assemblies of four coniferous tree species. J. For. Res. *29*, 151–157. https://doi.org/10.1080/13416979.2023.2267304.
38. Xu, J., and Shi, J.S. (2007). C banding and fluorescent banding pattern of the chromosome of *Cuninghamia lanceolate*. Mol. Plant Breed. *5*, 515–520.
39. Wu, J.J., Han, Y.W., Lin, C.F., Cai, J., and Zhao, Y.P. (2023). Benchmarking gene set of gymnosperms for assessing genome and annotation completeness in BUSCO. Hortic. Res. *10*, uhad165. https://doi.org/10.1093/hr/uhad165.

CellPress
OPEN ACCESS

40. Delcher, A.L., Phillippy, A., Carlton, J., and Salzberg, S.L. (2002). Fast algorithms for larger-scale genome alignment and comparison. Nucleic Acids Res. *30*, 2478–2483. https://doi.org/10.1093/nar/30.11.2478.

41. Marçais, G., Delcher, A.L., Phillippy, A.M., Coston, R., Salzberg, S.L., and Zimin, A. (2018). MUMmer4: A fast and versatile genome alignment system. PLoS Comput. Biol. *14*, e1005944. https://doi.org/10.1371/journal.pcbi.1005944.

42. Wan, T., Gong, Y., Liu, Z., Zhou, Y., Dai, C., and Wang, Q. (2022). Evolution of complex genome architecture in gymnosperms. GigaScience *11*, giac078. https://doi.org/10.1093/gigascience/giac078.

43. Cossu, R.M., Casola, C., Giacomello, S., Vidalis, A., Scofield, D.G., and Zuccolo, A. (2017). LTR retrotransposons show low levels of unequal recombination and high rates of intraelement gene conversion in large plant genomes. Genome Biol. Evol. *9*, 3449–3462. https://doi.org/10.1093/gbe/evx260.

44. Vinogradov, A.E. (1999). Intron-genome size relationship on a large evolutionary scale. J. Mol. Evol. *49*, 376–384. https://doi.org/10.1007/PL00006561.

45. Neale, D.B., Wegrzyn, J.L., Stevens, K.A., Zimin, A.V., Puiu, D., Crepeau, M.W., Cardeno, C., Koriabine, M., Holtz-Morris, A.E., Liechty, J.D., et al. (2014). Decoding the massive genome of loblolly pine using haploid DNA and novel assembly strategies. Genome Biol. *15*, R59. https://doi.org/10.1186/gb-2014-15-3-r59.

46. Stull, G.W., Qu, X.J., Parins-Fukuchi, C., Yang, Y.Y., Yang, J.B., Yang, Z.Y., Hu, Y., Ma, H., Soltis, P.S., Soltis, D.E., et al. (2021). Gene duplications and phylogenomic conflict underlie major pulses of phenotypic evolution in gymnosperms. Nat. Plants *7*, 1015–1025. https://doi.org/10.1038/s41477-021-00964-4.

47. Bergsten, J. (2005). A review of long-branch attraction. Cladistics *21*, 163–193. https://doi.org/10.1111/j.1096-0031.2005.00059.x.

48. Chen, H., Fang, Y., Zwaenepoel, A., Huang, S., Van de Peer, Y., and Li, Z. (2023). Revisiting ancient polyploidy in leptosporangiate ferns. New Phytol. *237*, 1405–1417. https://doi.org/10.1111/nph.18607.

49. Ronquist, F., Teslenko, M., Van der Mark, P., Ayres, D.L., Darling, A., Höhna, S., Larget, B., Liu, L., Suchard, M.A., and Huelsenbeck, J.P. (2012). MrBayes 3.2: efficient Bayesian phylogenetic inference and model choice across a large model space. Syst. Biol. *61*, 539–542. https://doi.org/10.1093/sysbio/sys029.

50. Zwaenepoel, A., and Van de Peer, Y. (2019). Inference of ancient Whole-Genome Duplications and the evolution of gene duplication and loss rates. Mol. Biol. Evol. *36*, 1384–1404. https://doi.org/10.1093/molbev/msz088.

51. Jin, W.T., Gernandt, D.S., Wehenkel, C., Xia, X.M., Wei, X.X., and Wang, X.Q. (2021). Phylogenomic and ecological analyses reveal the spatiotemporal evolution of global pines. Proc. Natl. Acad. Sci. USA *118*, e2022302118. https://doi.org/10.1073/pnas.2022302118.

52. De Bie, T., Cristianini, N., Demuth, J.P., and Hahn, M.W. (2006). CAFE: a computational tool for study of gene family evolution. Bioinformatics *22*, 1269–1271. https://doi.org/10.1093/bioinformatics/btl097.

53. Meng, X.Y., Chen, F., and Deng, S.W. (1988). Fossil plant *Cunninghamia asiatica* (Krassilov) Comb. Nov. Acta. Botanica Sinica *30*, 649–654.

54. Leslie, A.B., Beaulieu, J., Holman, G., Campbell, C.S., Mei, W., Raubeson, L.R., and Mathews, S. (2018). An overview of extant conifer evolution from the perspective of the fossil record. Am. J. Bot. *105*, 1531–1544. https://doi.org/10.1002/ajb2.1143.

55. Elias, S.A., Short, S.K., Nelson, C.H., and Birks, H.H. (1996). Life and times of the Bering Land Bridge. Nature *382*, 60–63. https://doi.org/10.1038/382060a0.

56. Denk, T., Grímsson, F., and Zetter, R. (2010). Episodic migration of oaks to Iceland: evidence for a North Atlantic "land bridge" in the Latest Miocene. Am. J. Bot. *97*, 276–287. https://doi.org/10.3732/ajb.0900195.

57. Hou, B. (1996). The origin and development history of Chinese Fir in China. Agricultural Archaeology *1*, 161–171.

58. Li, H.T., Yi, T.S., Gao, L.M., Ma, P.F., Zhang, T., Yang, J.B., Gitzendanner, M.A., Fritsch, P.W., Cai, J., Luo, Y., et al. (2019). Origin of angiosperms and the puzzle of the Jurassic gap. Nat. Plants *5*, 461–470. https://doi.org/10.1038/s41477-019-0421-0.

59. Hewitt, G. (2000). The genetic legacy of the Quaternary ice ages. Nature *405*, 907–913. https://doi.org/10.1038/35016000.

60. Tang, C.Q., Matsui, T., Ohashi, H., Dong, Y.F., Momohara, A., Herrando-Moraira, S., Qian, S., Yang, Y., Ohsawa, M., Luu, H.T., et al. (2018). Identifying long-trem stable refujia for relict plant species in East Asia. Nat. Commun. *9*, 4488. https://doi.org/10.1038/s41467-018-06837-3.

61. Keppel, G., Van Niel, K.P., Wardell-Johnson, G.W., Yates, C.J., Byrne, M., Mucina, L., Schut, A.G.T., Hopper, S.D., and Franklin, S.E. (2012). Regugia: identifying and understanding safe havens for biodiversity under climate change. Global Ecol. Biogeogr. *21*, 393–404. https://doi.org/10.1111/j.1466-8238.2011.00686.x.

62. Bao, L., Kudureti, A., Bai, W., Chen, R., Wang, T., Wang, H., and Ge, J. (2015). Contributions of multiple refugia during the last glacial period to current mainland populations if Korean Pine (*Pinus koraiensis*). Sci. Rep. *5*, 18608. https://doi.org/10.1038/srep18608.

63. Hilbert, D.W., Graham, A., and Hopkins, M.S. (2007). Glacial and interglacial refugia within a long-term rainforest refugium: The Wet Tropics Bioregion if NE Queensland, Australia. Palaeogeogr. Palaeoclimatol. Palaeoecol. *251*, 104–118. https://doi.org/10.1016/j.palaeo.2007.02.020.

64. Linkies, A., Graeber, K., Knight, C., and Leubner-Metzger, G. (2010). The evolution of seeds. New Phytol. *186*, 817–831. https://doi.org/10.1111/j.1469-8137.2010.03249.

65. Leslie, A.B., Beaulieu, J.M., and Mathews, S. (2017). Variation in seed size is structured by dispersal syndrome and cone morphology in conifers and other nonflowering seed plants. New Phytol. *216*, 429–437. https://doi.org/10.1111/nph.14456DigitalObjectIdentifier.

66. Zheng, P., Shen, M., Liu, R., Cai, X., Lin, J., Wang, L., Chen, Y., Chen, G., Cao, S., and Qin, Y. (2023). Revealing Further Insights into Astringent Seeds of Chinese Fir by Integrated Metabolomic and Lipidomic Analyses. Int. J. Mol. Sci. *24*, 15103. https://doi.org/10.3390/ijms242015103.

67. Chen, Y., Wu, Y., Wu, C., and Lin, S. (2020). Comparative analysis reveals the metabolic characteristics of astringent Seeds of Chinese Fir (*Cunninghamia lanceolata* (Lamb) Hook) during astringent compounds accumulation stages. Forests *11*, 1206. https://doi.org/10.3390/f11111206.

68. Yu, X., and Li, P. (1989). Studies of the abortive seed and its constituents in *Cunninghamia lanceolata* (Lamb.) Hook. Acta Bot. Boreali Occident. Sin. *9*, 252–256.

69. Meinke, D.W. (2020). Genome-wide identification of EMBRYO-DEFECTIVE (EMB) genes required for growth and development in *Arabidopsis*. New Phytol. *226*, 306–325. https://doi.org/10.1111/nph.16071.

70. Dutta, A., Chan, S.H.P., Pauli, N.T., and Raina, R. (2015). Hypersensitive response-like lesions 1 codes for *AtPPT1* and regulates accumulation of ROS and defense against bacterial pathogen Pseudomonas syringae in *Arabidopsis thaliana*. Antioxidants Redox Signal. *22*, 785–796. https://doi.org/10.1089/ars.2014.5963.

71. Zhang, M.J., Zhang, X.S., and Gao, X.Q. (2020). ROS in the Male-Female Interactions During Pollination: Function and Regulation. Front. Plant Sci. *11*, 177. https://doi.org/10.3389/fpls.2020.00177.

72. Wu, C., Gu, Z., Li, T., Yu, J., Liu, C., Fan, W., Wang, B., Jiang, F., Zhang, Q., and Li, W. (2021). The apple MdPTI1L kinase is phosphorylated by *MdOXI1* during S-RNase-induced reactive oxygen species signaling in pollen tubes. Plant Sci. *305*, 110824. https://doi.org/10.1016/j.plantsci.2021.110824.

73. Breygina, M., and Klimenko, E. (2020). ROS and Ions in Cell Signaling during Sexual Plant Reproduction. Int. J. Mol. Sci. *21*, 9476. https://doi.org/10.3390/ijms21249476.

74. Blanco, J.A., Wei, X., Jiang, H., Jie, C.Y., and Xin, Z.H. (2012). Impacts of enhanced nitrogen deposition and soil acidification on biomass

production and nitrogen leaching in Chinese fir plantations. Can. J. For. Res. *42*, 437–450. https://doi.org/10.1139/x2012-004.

75. Ye, Y., Hong, K., Zhang, J., Cao, G., Lin, S., Yang, Q., and Xu, S. (2020). Effects of aluminum stress on growth, photosynthetic characteristics and chloroplast ultrastructure in leaves of *Cunninghamia lanceolata* seedlings. Journal of Northeast Forsetry University *48*, 16.

76. Bandowe, B.A.M., Bigalke, M., Kobza, J., and Wilcke, W. (2018). Sources and fate of polycyclic aromatic compounds (PAHs, oxygenated PAHs and azaarenes) in forest soil profiles opposite of an aluminium plant. Sci. Total Environ. *630*, 83–95. https://doi.org/10.1016/j.scitotenv.2018.02.109.

77. Mu, S.Y., Yang, Y.T., Qu, X.Y., Wang, F.F., Ma, F.F., Ding, Z.N., Ye, L.P., Zhang, Y.L., Zhang, J.J., Lyu, M.M., et al. (2024). A potential role of a special type of abortive seeds in *Cunninghamia lanceolata*: promoting the growth of healthy seedlings in active alumium ions-rich soil. Front. Plant Sci. *15*, 1482355. https://doi.org/10.3389/fpls.2024.1482355.

78. Bradová, M., Tejnecký, V., Borůvka, L., Němeček, K., Ash, C., Šebek, O., Svoboda, M., Zenáhlíková, J., and Drábek, O. (2015). The variations of aluminium species in mountainous forest soils and its implications to soil acidification. Environ. Sci. Pollut. Res. Int. *22*, 16676–16687. https://doi.org/10.1007/s11356-015-4855-2.

79. Hubová, P., Tejnecký, V., Češková, M., Borůvka, L., Němeček, K., and Drábek, O. (2018). Behaviour of aluminium in forest soils with different lithology and herb vegetation cover. J. Inorg. Biochem. *181*, 139–144. https://doi.org/10.1016/j.jinorgbio.2017.09.017.

80. Inostroza-Blancheteau, C., Rengel, Z., Alberdi, M., de la Luz Mora, M., Aquea, F., Arce-Johnson, P., and Reyes-Díaz, M. (2012). Molecular and physiological strategies to increase aluminum resistance in plants. Mol. Biol. Rep. *39*, 2069–2079. https://doi.org/10.1007/s11033-011-0954-4.

81. Crane, P.R., Friis, E.M., and Pedersen, K.R. (1995). The origin and early diversification of angiosperms. Nature *374*, 27–33. https://doi.org/10.1038/374027a0.

82. Melzer, R., Wang, Y.Q., and Theißen, G. (2010). The naked and the dead: the ABCs of gymnosperm reproduction and the origin of the angiosperm flower. Semin. Cell Dev. Biol. *21*, 118–128. https://doi.org/10.1016/j.semcdb.2009.11.015.

83. Bowman, J.L., Smyth, D.R., and Meyerowitz, E.M. (1991). Genetic interactions among floral homeotic genes. Development *112*, 1–20. https://doi.org/10.1242/dev.112.1.1.

84. Coen, E.S., and Meyerowitz, E.M. (1991). The war of the whorls: genetic interactions controlling flower development. Nature *353*, 31–37. https://doi.org/10.1038/353031a0.

85. Theißen, G., and Saedler, H. (2001). Floral quartets. Nature *409*, 469–471. https://doi.org/10.1038/35054172.

86. Ditta, G., Pinyopich, A., Robles, P., Pelaz, S., and Yanofsky, M.F. (2004). The *SEP4* gene of *Arabidopsis thaliana* functions in floral organ and meristem identity. Curr. Biol. *14*, 1935–1940. https://doi.org/10.1016/j.cub.2004.10.028.

87. Bowman, J.L., Smyth, D.R., and Meyerowitz, E.M. (2012). The ABC model of flower development: then and now. Development *139*, 4095–4098. https://doi.org/10.1242/dev.083972.

88. Theißen, G., Melzer, R., and Rümpler, F. (2016). MADS-domain transcription factors and the floral quartet model of flower development: linking plant development and evolution. Development *143*, 3259–3271. https://doi.org/10.1242/dev.134080.

89. Gramzow, L., and Theißen, G. (2010). A hitchhiker's guide to the MADS world of plants. Genome Biol. *11*, 214. https://doi.org/10.1186/gb-2010-11-6-214.

90. Gramzow, L., and Theißen, G. (2013). Phylogenomics of MADS-box genes in plants — two opposing life styles in one gene family. Biology *2*, 1150–1164. https://doi.org/10.3390/biology2031150.

91. Gramzow, L., and Theißen, G. (2015). Phylogenomics reveals surprising sets of essential and dispensable clades of MIKCc-group MADS-box genes in flowering plants. J. Exp. Zool. B Mol. Dev. Evol. *324*, 353–362. https://doi.org/10.1002/jez.b.22598.

92. Ruelens, P., de Maagd, R.A., Proost, S., Theißen, G., Geuten, K., and Kaufmann, K. (2013). FLOWERING LOCUS C in monocots and the tandem origin of angiosperm-specific MADS-box genes. Nat. Commun. *4*, 2280. https://doi.org/10.1038/ncomms3280.

93. Causier, B., Schwarz-Sommer, Z., and Davies, B. (2010). Floral organ identity: 20 years of ABCs. Semin. Cell Dev. Biol. *21*, 73–79. https://doi.org/10.1016/j.semcdb.2009.10.005.

94. Chen, F., Zhang, X., Liu, X., and Zhang, L. (2017). Evolutionary analysis of MIKC(c)-Type MADS-Box genes in gymnosperms and angiosperms. Front. Plant Sci. *8*, 895. https://doi.org/10.3389/fpls.2017.00895.

95. Zhang, L., Chen, F., Zhang, X., Li, Z., Zhao, Y., Lohaus, R., Chang, X., Dong, W., Ho, S.Y.W., Liu, X., et al. (2020). The water lily genome and the early evolution of flowering plants. Nature *577*, 79–84. https://doi.org/10.1038/s41586-019-1852-5.

96. Dreni, L., and Zhang, D. (2016). Flower development: the evolutionary history and functions of the *AGL6* subfamily MADS-box genes. J. Exp. Bot. *67*, 1625–1638. https://doi.org/10.1093/jxb/erw046.

97. Thompson, B.E., Bartling, L., Whipple, C., Hall, D.H., Sakai, H., Schmidt, R., and Hake, S. (2009). bearded-ear encodes a MADS box transcription factor critical for maize floral development. Plant Cell *21*, 2578–2590. https://doi.org/10.1105/tpc.109.067751.

98. Hsu, W.H., Yeh, T.J., Huang, K.Y., Li, J.Y., Chen, H.Y., and Yang, C.H. (2014). *AGAMOUS-LIKE13*, a putative ancestor for the E functional genes, specifies male and female gametophyte morphogenesis. Plant J. *77*, 1–15. https://doi.org/10.1111/tpj.12363.

99. Theißen, G., and Becker, A. (2004). Gymnosperm orthologues of class B floral homeotic genes and their impact on understanding flower origin. Crit. Rev. Plant Sci. *23*, 129–148. https://doi.org/10.1080/07352680490433240.

100. Theißen, G., Becker, A., Di Rosa, A., Kanno, A., Kim, J.T., Münster, T., Winter, K.U., and Saedler, H. (2000). A short history of MADS-box genes in plants. Plant Mol. Biol. *42*, 115–149. https://doi.org/10.1007/978-94-011-4221-2_6.

101. Mouradov, A., Glassick, T.V., Hamdorf, B.A., Murphy, L.C., Marla, S.S., Yang, Y., Teasdale, R.D., and Yang, Y.M. (1998). Family of MADS-Box genes expressed early in male and female reproductive structures of monterey pine. Plant Physiol. *117*, 55–62. https://doi.org/10.1104/pp.117.1.55.

102. Ranallo-Benavidez, T.R., Jaron, K.S., and Schatz, M.C. (2020). GenomeScope 2.0 and Smudgeplot for reference-free profiling of polyploid genomes. Nat. Commun. *11*, 1432. https://doi.org/10.1038/s41467-020-14998-3.

103. Li, H., and Durbin, R. (2009). Fast and accurate short read alignment with Burrows-Wheeler transform. Bioinformatics *25*, 1754–1760. https://doi.org/10.1093/bioinformatics/btp324.

104. Koren, S., Walenz, B.P., Berlin, K., Miller, J.R., Bergman, N.H., and Phillippy, A.M. (2017). Canu: scalable and accurate long-read assembly via adaptive k-mer weighting and repeat separation. Genome Res. *27*, 722–736. https://doi.org/10.1101/gr.215087.116.

105. Ruan, J., and Li, H. (2020). Fast and accurate long-read assembly with Wtdbg2. Nat. Methods *17*, 155–158. https://doi.org/10.1038/s41592-019-0669-3.

106. Walker, B.J., Abeel, T., Shea, T., Priest, M., Abouelliel, A., Sakthikumar, S., Cuomo, C.A., Zeng, Q., Wortman, J., Young, S.K., and Earl, A.M. (2014). Pilon: an integrated tool for comprehensive microbial variant detection and genome assembly improvement. PLoS One *9*, e112963. https://doi.org/10.1371/journal.pone.0112963.

107. Manni, M., Berkeley, M.R., Seppey, M., Simão, F.A., and Zdobnov, E.M. (2021). BUSCO Update: Novel and Streamlined Workflows along with

CellPress
OPEN ACCESS

Broader and Deeper Phylogenetic Coverage for Scoring of Eukaryotic, Prokaryotic, and Viral Genomes. Mol. Biol. Evol. *38*, 4647–4654. https://doi.org/10.1093/molbev/msab199.

108. Servant, N., Varoquaux, N., Lajoie, B.R., Viara, E., Chen, C.J., Vert, J.P., Heard, E., Dekker, J., and Barillot, E. (2015). HiC-Pro: an optimized and flexible pipeline for Hi-C data processing. Genome Biol. *16*, 259. https://doi.org/10.1186/s13059-015-0831-x.

109. Burton, J.N., Adey, A., Patwardhan, R.P., Qiu, R., Kitzman, J.O., and Shendure, J. (2013). Chromosome-scale scaffolding of de novo genome assemblies based on chromatin interactions. Nat. Biotechnol. *31*, 1119–1125. https://doi.org/10.1038/nbt.2727.

110. Burge, C., and Karlin, S. (1997). Prediction of complete gene structures in human genomic DNA. J. Mol. Biol. *268*, 78–94. https://doi.org/10.1006/jmbi.1997.0951.

111. Stanke, M., and Waack, S. (2003). Gene prediction with a hidden Markov model and a new intron submodel. Bioinformatics *19*, ii215–ii225. https://doi.org/10.1093/bioinformatics/btg1080.

112. Majoros, W.H., Pertea, M., and Salzberg, S.L. (2004). TigrScan and GlimmerHMM: two open source ab initio eukaryotic gene-finders. Bioinformatics *20*, 2878–2879. https://doi.org/10.1093/bioinformatics/bth315.

113. Alioto, T., Blanco, E., Parra, G., and Guigó, R. (2018). Using geneid to identify genes. Curr. Protoc. Bioinform. *64*, e56. https://doi.org/10.1002/cpbi.56.

114. Johnson, A.D., Handsaker, R.E., Pulit, S.L., Nizzari, M.M., O'Donnell, C.J., and de Bakker, P.I.W. (2008). SNAP: a web-based tool for identification and annotation of proxy SNPs using HapMap. Bioinformatics *24*, 2938–2939. https://doi.org/10.1093/bioinformatics/btn564.

115. Keilwagen, J., Wenk, M., Erickson, J.L., Schattat, M.H., Grau, J., and Hartung, F. (2016). Using intron position conservation for homology-based gene prediction. Nucleic Acids Res. *44*, e89. https://doi.org/10.1093/nar/gkw092.

116. Kim, D., Langmead, B., and Salzberg, S.L. (2015). HISAT: a fast spliced aligner with low memory requirements. Nat. Methods *12*, 357–360. https://doi.org/10.1038/nmeth.3317.

117. Pertea, M., Kim, D., Pertea, G.M., Leek, J.,T., and Salzberg, S.L. (2016). Transcript-level expression analysis of RNA-seq experiments with HISAT, StringTie and Ball- gown. Nat. Protoc. *11*, 1650–1667. https://doi.org/10.1038/nprot.2016.095.

118. Tang, S., Lomsadze, A., and Borodovsky, M. (2015). Identification of protein coding regions in RNA transcripts. Nucleic Acids Res. *43*, e78. https://doi.org/10.1093/nar/gkv227.

119. Haas, B.J., Delcher, A.L., Mount, S.M., Wortman, J.R., Smith, R.K., Jr., Hannick, L.I., Maiti, R., Ronning, C.M., Rusch, D.B., Town, C.D., et al. (2003). Improving the *Arabidopsis* genome annotation using maximal transcript alignment assemblies. Nucleic Acids Res. *31*, 5654–5666. https://doi.org/10.1093/nar/gkg770.

120. Haas, B.J., Salzberg, S.L., Zhu, W., Pertea, M., Allen, J.E., Wortman, J.R., Orvis, J., White, O., and Buell, C.R. (2008). Automated eukaryotic gene structure annotation using EVidenceModeler and the program to assemble spliced alignments. Genome Biol. *9*, R7. https://doi.org/10.1186/gb-2008-9-1-r7.

121. Altschul, S.F., Gish, W., Miller, W., Myers, E.W., and Lipman, D.J. (1990). Basic local alignment search tool. J. Mol. Biol. *215*, 403–410. https://doi.org/10.1016/S0022-2836(05)80360-2.

122. Nawrocki, E.P., Kolbe, D.L., and Eddy, S.R. (2009). Infernal 1.0: inference of RNA alignments. Bioinformatics *25*, 1335–1337. https://doi.org/10.1093/bioinformatics/btp157.

123. Lowe, T.M., and Eddy, S.R. (1997). tRNAscan-SE: a program for improved detection of transfer RNA genes in genomic sequence. Nucleic Acids Res. *25*, 955–964. https://doi.org/10.1093/nar/25.5.955.

124. She, R., Chu, J.S.C., Wang, K., Pei, J., and Chen, N. (2009). GenBlastA: enabling BLAST to identify homologous gene sequences. Genome Res. *19*, 143–149. https://doi.org/10.1101/gr.082081.108.

125. Birney, E., Clamp, M., and Durbin, R. (2004). GeneWise and Genomewise. Genome Res. *14*, 988–995. https://doi.org/10.1101/gr.1865504.

126. Zhao, X., and Hao, W. (2007). LTR_FINDER: an efficient tool for the prediction of full-length LTR retrotransposons. Nucleic Acids Res. *35*, W265–W268. https://doi.org/10.1093/nar/gkm286.

127. Edgar, R.C., and Myers, E.W. (2005). PILER: identification and classification of genomic repeats. Bioinformatics *21*, i152–i158. https://doi.org/10.1093/bioinformatics/bti1003.

128. Hoede, C., Arnoux, S., Moisset, M., Chaumier, T., Inizan, O., Jamilloux, V., and Quesneville, H. (2014). PASTEC: an automatic transposable element classification tool. PLoS One *9*, e91929. https://doi.org/10.1371/journal.pone.0091929.

129. Tarailo-Graovac, M., and Chen, N. (2009). Using RepeatMasker to identify repetitive elements in genomic sequences. Curr. Protoc. Bioinform. *25*. https://doi.org/10.1002/0471250953.bi0410s25.

130. Ou, S., and Jiang, N. (2018). LTR_retriever: a highly accurate and sensitive program for identification of long terminal repeat retrotransposons. Plant Physiol. *176*, 1410–1422. https://doi.org/10.1104/pp.17.01310.

131. Li, L., Stoeckert, C.J., Jr, and Roos, D.S. (2003). OrthoMCL: identification of ortholog groups for eukaryotic genomes. Genome Res. *13*, 2178–2189. https://doi.org/10.1101/gr.1224503.

132. Edgar, R.C. (2004). MUSCLE: multiple sequence alignment with high accuracy and high throughput. Nucleic Acids Res. *32*, 1792–1797. https://doi.org/10.1093/nar/gkh340.

133. Capella-Gutiérrez, S., Silla-Martínez, J.M., and Gabaldón, T. (2009). Trimal: a tool for automated alignment trimming in large-scale phylogenetic analyses. Bioinformatics *25*, 1972–1973. https://doi.org/10.1093/bioinformatics/btp348.

134. Stamatakis, A. (2015). Using RAxML to infer phylogenies. Curr. Protoc. Bioinform. *51*, 6.14.1–6.14.14. https://doi.org/10.1002/0471250953.bi0614s51.

135. Yang, Z. (2007). PAML 4: phylogenetic analysis by maximum likelihood. Mol. Biol. Evol. *24*, 1586–1591. https://doi.org/10.1093/molbev/msm088.

136. Chen, H., and Zwaenepoel, A. (2023). Inference of Ancient Polyploidy from Genomic Data. In Polyploidy: Methods and Protocols, Y. Van de Peer, ed. (New York, NY: Springer US), pp. 3–18.

137. Buchfink, B., Reuter, K., and Drost, H.G. (2021). Sensitive protein alignments at tree-of-life scale using DIAMOND. Nat. Methods *18*, 366–368. https://doi.org/10.1038/s41592-021-01101-x.

138. van Dongen, S. (2008). Graph clustering via a discrete uncoupling process. SIAM J. Matrix Anal. Appl. *30*, 121–141. https://doi.org/10.1137/040608635.

139. Proost, S., Fostier, J., De Witte, D., Dhoedt, B., Demeester, P., Van de Peer, Y., and Vandepoele, K. (2012). i-ADHoRe 3.0—fast and sensitive detection of genomic homology in extremely large data sets. Nucleic Acids Res. *40*, e11. https://doi.org/10.1093/nar/gkr955.

140. Tang, H., Krishnakumar, V., Zeng, X., Xu, Z., Taranto, A., Lomas, J.S., Zhang, Y., Huang, Y., Wang, Y., Yim, W.C., et al. (2024). JCVI: A versatile toolkit for comparative genomics analysis. iMeta *3*, e211. https://doi.org/10.1002/imt2.211.

141. Emms, D.M., and Kelly, S. (2019). OrthoFinder: phylogenetic orthology inference for comparative genomics. Genome Biol. *20*, 238. https://doi.org/10.1186/s13059-019-1832-y.

142. Löytynoja, A., and Goldman, N. (2005). An algorithm for progressive multiple alignment of sequences with insertions. Proc. Natl. Acad. Sci. USA *102*, 10557–10562. https://doi.org/10.1073/pnas.0409137102.

143. Capella-Gutiérrez, S., Silla-Martínez, J.M., and Gabaldón, T. (2009). TrimAl: a tool for automated alignment trimming in large-scale phylogenetic analyses. Bioinformatics *25*, 1972–1973. https://doi.org/10.1093/bioinformatics/btp348.

144. Szöllősi, G.J., Rosikiewicz, W., Boussau, B., Tannier, E., and Daubin, V. (2013). Efficient exploration of the space of reconciled gene trees. Syst. Biol. *62*, 901–912. https://doi.org/10.1093/sysbio/syt054.

145. Kumar, S., Suleski, M., Craig, J.M., Kasprowicz, A.E., Sanderford, M., Li, M., Stecher, G., and Hedges, S.B. (2022). TimeTree 5: an expanded resource for species divergence times. Mol. Biol. Evol. *39*, msac174. https://doi.org/10.1093/molbev/msac174.

146. Kumar, S., Stecher, G., Li, M., Knyaz, C., and Tamura, K. (2018). MEGA X: Molecular Evolutionary Genetics Analysis across Computing Platforms. Mol. Biol. Evol. *35*, 1547–1549. https://doi.org/10.1093/molbev/msy096.

147. Alexander, D.H., Novembre, J., and Lange, K. (2009). Fast Model-based estimation of ancestry in unrelated individuals. Genome Res. *9*, 19. https://doi.org/10.1101/gr.094052.109.

148. Letunic, I., Goodstadt, L., and Dickens, N.J. (2002). Recent improvements to the SMART domain-based sequence annotation resource. Nucleic Acids Res. *30*, 242–244. https://doi.org/10.1093/nar/30.1.242.

149. Katoh, K., and Standley, D.M. (2013). MAFFT multiple sequence alignment software version 7: improvements in performance and usability. Mol. Biol. Evol. *30*, 772–780. https://doi.org/10.1093/molbev/mst010.

150. Price, M.N., Dehal, P.S., and Arkin, A.P. (2010). FastTree 2–approximately maximum-likelihood trees for large alignments. PLoS One *5*, e9490. https://doi.org/10.1371/journal.pone.0009490.

151. Rao, S.S.P., Huntley, M.H., Durand, N.C., Stamenova, E.K., Bochkov, I.D., Robinson, J.T., Sanborn, A.L., Machol, I., Omer, A.D., Lander, E.S., and Aiden, E.L. (2014). A 3D map of the human genome at kilobase resolution reveals principles of chromatin looping. Cell *159*, 1665–1680. https://doi.org/10.1016/j.cell.2014.11.021.

152. Griffiths-Jones, S., Moxon, S., Marshall, M., Khanna, A., Eddy, S.R., and Bateman, A. (2005). Rfam: annotating non-coding RNAs in complete genomes. Nucleic Acids Res. *33*, D121–D124. https://doi.org/10.1093/nar/gki081.

153. Jurka, J., Kapitonov, V.V., Pavlicek, A., Klonowski, P., Kohany, O., and Walichiewicz, J. (2005). Repbase Update, a database of eukaryotic repetitive elements. Cytogenet. Genome Res. *110*, 462–467. https://doi.org/10.1159/000084979.

154. Goodstein, D.M., Shu, S., Howson, R., Neupane, R., Hayes, R.D., Fazo, J., Mitros, T., Dirks, W., Hellsten, U., Putnam, N., and Rokhsar, D.S. (2012). Phytozome: a comparative platform for green plant genomics. Nucleic Acids Res. *40*, D1178–D1186. https://doi.org/10.1093/nar/gkr944.

155. Sundell, D., Mannapperuma, C., Netotea, S., Delhomme, N., Lin, Y.C., Sjödin, A., Van de Peer, Y., Jansson, S., Hvidsten, T.R., and Street, N.R. (2015). The plant genome integrative explorer resource: plantgenie.org. New Phytol. *208*, 1149–1156. https://doi.org/10.1111/nph.13557.

156. Sneddon, T.P., Li, P., and Edmunds, S.C. (2012). GigaDB: announcing the GigaScience database. GigaScience *1*, 11. https://doi.org/10.1186/2047-217X-1-11.

157. Chen, M., Ma, Y., Wu, S., Zheng, X., Kang, H., Sang, J., Xu, X., Hao, L., Li, Z., Gong, Z., et al. (2021). Genome Warehouse: a public repository housing genome-scale data. Genom. Proteom. Bioinf. *19*, 584–589. https://doi.org/10.1016/j.gpb.2021.04.001.

158. Li, F.W., Brouwer, P., Carretero-Paulet, L., Cheng, S., de Vries, J., Delaux, P.M., Eily, A., Koppers, N., Kuo, L.Y., Li, Z., et al. (2018). Fern genomes elucidate land plant evolution and cyanobacterial symbioses. Nat. Plants *4*, 460–472. https://doi.org/10.1038/s41477-018-0188-8.

159. Jiao, Y., Wickett, N.J., Ayyampalayam, S., Chanderbali, A.S., Landherr, L., Ralph, P.E., Tomsho, L.P., Hu, Y., Liang, H., Soltis, P.S., et al. (2011). Ancestral polyploidy in seed plants and angiosperms. Nature *473*, 97–100. https://doi.org/10.1038/nature09916.

160. Ruprecht, C., Lohaus, R., Vanneste, K., Mutwil, M., Nikoloski, Z., Van de Peer, Y., and Persson, S. (2017). Revisiting ancestral polyploidy in plants. Sci. Adv. *3*, e1603195. https://doi.org/10.1126/sciadv.1603195.

161. Sun, X., Liu, D., Zhang, X., Li, W., Liu, H., Hong, W., Jiang, C., Guan, N., Ma, C., Zeng, H., et al. (2013). SLAF-seq: an efficient method of large-scale de novo SNP discovery and genotyping using high-throughput sequencing. PLoS One *8*, e58700. https://doi.org/10.1371/journal.pone.0058700.

162. Mistry, J., Chuguransky, S., Williams, L., Qureshi, M., Salazar, G.A., Sonnhammer, E.L.L., Tosatto, S.C.E., Paladin, L., Raj, S., Richardson, L.J., et al. (2021). Pfam: The protein families database in 2021. Nucleic Acids Res. *49*, D412–D419. https://doi.org/10.1093/nar/gkaa913.

STAR★METHODS

KEY RESOURCES TABLE

REAGENT or RESOURCE	SOURCE	IDENTIFIER
Bacterial and virus strains		
E. coli JM109	TaKaRa	ATCC: 53323
Agrobacterium GV3101	Lab owned	DSM: 12364
Biological samples		
C.lanceolata: leaf	This work	N/A
C.lanceolata: astringent seed	This work	N/A
C.lanceolata: germinating seed	This work	N/A
C.lanceolata: phloem	This work	N/A
C.lanceolata: stem	This work	N/A
C.lanceolata: root	This work	N/A
C.lanceolata: homozygous female gametophytes	This work	N/A
C.lanceolata: male cones	This work	N/A
C.lanceolata: female cones	This work	N/A
C.lanceolata: seed scales	This work	N/A
C.lanceolata: bract scales	This work	N/A
A.thaliana: seed	This work	N/A
Critical commercial assays		
P6-C4	Pacific Biosciences(PacBio)	N/A
HiSeq X Reagent Kit	Illumina	N/A
NovaSeq 6000 Reagent Kit	Illumina	N/A
Deposited data		
whole-genome sequencing data, Illumina data, and genome assembly sequences	This work	NCBI BioProject: PRJNA668674
Oligonucleotides		
ANR3301-F: 5'TGACCTCGAGACTAGT ATGAGTTGCACTAAGAAGGT3'	This work	N/A
ANR3301-R: 5'TGTAGTCCATACTAGTGTT ACCGACATCATTAGAGC3'	This work	N/A
LAR3301-F: 5'TGACCTCGAGACTAGTATGG CCTGTGCTCCCAAAGT3'	This work	N/A
LAR3301-R: 5'TGTAGTCCATACTAG TAAGGTACTGGTTGAAAAAAT3'	This work	N/A
DET23301-F: 5'TGACCTCGAGACTAGTAT GGCGCATGTTCTGCAACA3'	This work	N/A
DET23301-R: 5'TGTAGTCCATACTAGTGAATA TGAAGGGGAAAAGAG3'	This work	N/A
BZR13301-F: 5'TGACCTCGAGACTAGTAT GTCCACCCGCTCCGTAAT3'	This work	N/A
BZR13301-R: 5'TGTAGTCCATACTAGTGGGAC AAAAATGTTGAGACA3'	This work	N/A
Recombinant DNA		
pCambia3301	Lab owned	RRID: Addgene_210757

(*Continued on next page*)

Continued

REAGENT or RESOURCE	SOURCE	IDENTIFIER
Software and algorithms		
FASTX Toolkit (ver. 0.0.11)	Hannon Lab	https://github.com/agordon/fastx_toolkit
GenomeScope	Ranallo-Benavidez et al.[102]	http://genomescope.org
Bwa software	Li et al.[103]	http://maq.sourceforge.net
Canu v1.5	Koren et al.[104]	https://github.com/marbl/canu
WTDBG2	Ruan et al.[105]	https://github.com/ruanjue/wtdbg
Pilon v1.22	Walker[106]	https://github.com/broadinstitute/pilo
BUSCO v5.8.2	Manni et al.[107]	https://gitlab.com/ezlab/busco
HiC-Pro v2.8.1	Servant et al.[108]	https://github.com/nicmoya/HiC-Pro
LACHESIS	Burton et al.[109]	https://github.com/shendurelab/LACHESIS
GenScan v3.1	Burge et al.[110]	https://pbil.univ-lyon1.fr/members/duret/cours/INSA/exercise4/pgscan.html
Augustu v3.1	Stanke et al.[111]	http://augustus.gobics.de
Glimmer HMM v3.0.4	Majoros et al.[112]	https://ccb.jhu.edu/software/glimmerhmm/
Gene ID v1.4	Alioto et al.[113]	https://github.com/guigolab/geneid
SNAP v 2006-07-28	Johnson et al.[114]	http://homepage.mac.com/iankorf
GeMoMa v1.3.1	Keilwagen et al.[115]	https://www.jstacs.de/index.php/GeMoMa
Hisat v2.0.4	Kim et al.[116]	https://ccb.jhu.edu/software/hisat/index.shtml
StringTie v1.2.3	Pertea et al.[117]	https://ccb.jhu.edu/software/stringtie/
TransDecoder v2.0	Haas, BJ	https://github.com/TransDecoder/TransDecoder
GeneMarkS-T v5.1	Tang et al.[118]	https://exon.gatech.edu/
PASA v2.0.2	Haas et al.[119]	https://github.com/PASApipeline
EVM v1.1.1	Haas et al.[120]	https://github.com/EVidenceModeler
BLAST v2.2.31	Altschul et al.[121]	https://blast.ncbi.nlm.nih.gov/Blast.cgi?CMD=Web&PAGE_TYPE=BlastHome
Infernal v1.1.1	Nawrocki et al.[122]	http://infernal.janelia.org/
tRNAscan-SE 1.3.1	Lowe et al.[123]	https://trna.ucsc.edu/tRNAscan-SE/
GenBlastA v1.0.4	She et al.[124]	http://genome.sfu.ca/projects/genBlastA/
GeneWise v2.4.1	Birney et al.[125]	https://www.ebi.ac.uk/~birney/wise2/
LTR_FINDER v. 1.06	Zhao et al.[126]	http://tlife.fudan.edu.cn/ltr_finder/
PILER-DF v 2.4	Edgar et al.[127]	https://www.drive5.com/piler/
PASTEClassifier v1.0	Hoede et al.[128]	http://urgi.versailles.inra.fr/Tools/PASTEClassifier
RepeatMasker v. 4.0.5	Tarailo-Graovac et al.[129]	http://www.repeatmasker.org/RepeatModeler/
LTR retriever	Ou et al.[130]	https://github.com/oushujun/LTR_retriever
OrthoMCL v1.4	Li et al.[131]	http://orthomcl.org/orthomcl/
MUSCLE v3.8.31	Edgar et al.[132]	http://www.drive5.com/muscle/
Trimal	Capella-Gutierrez et al.[133]	https://github.com/inab/trimal
RAxML	Stamatakis[134]	https://github.com/stamatak/standard-RAxML
PAML4.9	Yang[135]	http://abacus.gene.ucl.ac.uk/software/paml.html
CAFÉ 4.2	De Bie et al.[52]	https://github.com/hahnlab/CAFE
Wgd v1.1.2	Chen et al.[136]	https://github.com/heche-psb/wgd
Diamond v2.1.11	Buchfink et al.[137]	https://github.com/bbuchfink/diamond
MCL	van Dongen[138]	http://www.micans.org/mcl/

(*Continued on next page*)

Continued

REAGENT or RESOURCE	SOURCE	IDENTIFIER
i-adhore v3.0.01	Proost et al.[139]	http://bioinformatics.psb.ugent.be/webtools/i-adhore/
JCVI	Tang et al.[140]	https://github.com/tanghaibao/jcvi
OrthoFinder v2.3.3	Emms et al.[141]	https://github.com/davidemms/OrthoFinder
PRANK	Löytynoja et al.[142]	http://wasabiapp.org/software/prank/
trimAl	Capella-Gutiérrez et al.[143]	https://vicfero.github.io/trimal/
WHALE	Zwaenepoel et al.[50]	https://github.com/arzwa/Whale.jl
Mrbayes v.3.2.6	Huelsenbeck et al.[49]	https://github.com/NBISweden/MrBayes
ALEobserve	Szöllősi et al.[144]	https://github.com/ssolo/ALE
TimeTree	Kumar et al.[145]	https://timetree.org/
MEGAX	Kumar et al.[146]	https://www.megasoftware.net/
ADMIXTURE v 1.3.0	Alexander et al.[147]	https://dalexander.github.io/admixture/
SMART	Letunic et al.[148]	http://smart.embl-heidelberg.de/
MAFFT	Katoh et al.[149]	https://mafft.cbrc.jp/alignment/software/
FastTree v2.1.10	Price et al.[150]	https://github.com/morgannprice/fasttree
Figtree v1.4.4	Andrew Rambaut	http://tree.bio.ed.ac.uk/software/figtree/

EXPERIMENTAL MODEL AND STUDY PARTICIPANT DETAILS

Plant materials and growing conditions

All genome sequencing materials used in this study were collected in April 2016 from a 10-year-old *C. lanceolata* plant cultivated in the third-generation seed orchard of *C. lanceolata* at the Youxi National Forest Farm in Fujian Province, China. The geographical coordinates of the orchard are 25°50 ’ – 26°26’ N, 117°48 ’ – 118°39’ E.

METHOD DETAILS

DNA preparation and sequencing

All genome sequencing materials used in this study were sourced from an adult *C. lanceolata* plant cultivated in the third-generation seed orchard of *C. lanceolata* at the Youxi National Forest Farm in Fujian Province, China. A sodium dodecyl sulphate-based lysis method was used to extract total genomic DNA from young leaves for Illumina and PacBio sequencing. For Illumina sequencing, the DNA was ultrasonicated to a fragment size of 270 bp, followed by library preparation using an Ultra DNA Library Prep Kit (NEB, 240 County Road, Ipswich, USA) according to the manufacturer’s instructions. Library sequencing was performed using a Hiseq4000 platform, generating paired-end sequencing reads. These reads were then trimmed using Fastq _quality_trimmer in the FASTX Toolkit ver. 0.0.11 (https://github.com/agordon/fastx_toolkit) with default parameters. For PacBio sequencing, DNA was interrupted using g-TUBE (Covaris), and the SMRTbell template preparation, involving DNA concentration, damage repair, end repair, hairpin adapter ligation, and template purification, was performed using the AMPure PB Magnetic Beads (Pacific Biosciences, 1305 O’Brien Drive, Menlo Park, USA). Subsequently, The the PacBio Sequel platform was employed to perform 20-kb single-molecule real-time DNA sequencing.

Genome size and heterozygosity estimation

To estimate the genome size and heterozygosity of *C. lanceolata*, we constructed eight 270-bp paired-end libraries and generated a *K*-mer distribution map. As shown in Figure S1, the average *K*-mer depth corresponding to the main peak was 39, from which we inferred the genome size based on *K*-mer number/*K*-mer depth. In addition, the *K*-mer depth of the small peak to the left of the main peak was 20. We estimated the *C. lanceolata* genome size to be 10.42 Gb using the GenomeScope.[102] For heterozygosity estimation, we employed the combined SNP calling results obtained upon comparing the 217 Gb Illumina data with the assembled *C. lanceolata* genome using the Bwa software.[103] This allowed us to determine the number of heterozygous SNPs in the genome and calculate the rate of heterozygosity. In total, we obtained 77 334 119 SNPs, with 77 050 655 being heterozygous and 283,464 being homozygous. Consequently, the heterozygosity of the Chinese fir genome was estimated to be 0.69%.

Genome assembly

The assembly of the *C. lanceolata* genome involved three main steps. Initially, Canu v1.5 (available at https://github.com/marbl/canu)[104] was utilised to correct errors in the clean PacBio data. Canu selects longer seed reads (genomeSize = 1000000000’ and

'corOutCoverage = 50'), detects clean-read overlaps using the high-sensitive overlapper MHAP (map-2.1.2, option 'corMhapSensitivity = low/normal/high'), and performs an error correction through the falcon_sense method (option 'correctedErrorRate = 0.025'). Subsequently, error-corrected reads were trimmed using unsupported bases and hairpin adapters to obtain the longest supported range using default parameters. Finally, Canu generated a draft assembly using the longest 80 coverage-trimmed reads. Additionally, WTDBG2 (https://github.com/ruanjue/wtdbg)[105] was employed to construct draft assembly reads. WTDBG2 initially generated a draft assembly with the parameter 'wtdbg -i pbreads.fasta -t 64 -H -k 21 -S 1.02 -e 3 -o wtdbg' and then utilized error-corrected reads from Canu to enhance draft assembly performance. The consensus draft assembly results were obtained with the parameter 'wtdbg-cns -t 64 -i wtdbg.ctg.lay -o wtdbg.ctg.lay.fa -k 15', followed by three rounds of polishing using Illumina data through Pilon v1.22 (https://github.com/broadinstitute/pilon).[106] The first polishing step adopted a quiver/arrow algorithm using SMS data with 40 threads, while the second polishing adopted the Pilon algorithm using Illumina data with the parameters '–mindepth 10 –changes –threads 4 –fix bases'. The completeness of the *C. lanceolata* genome assembly was evaluated against the embryophyta_odb10 lineage (n = 1375) using BUSCO v5.8.2.[107] Additionally, BWA was used to compare the short sequences obtained through Illumina sequencing with the assembled genome to assess its integrity.

Hi-C library construction and chromosome assembly

Hi-C fragment libraries, ranging from 300 to 700 bp in insert size, were constructed as described by Rao et al.[151] These libraries were then sequencing using Illumina high-throughput sequencing technology, with sequencing read lengths set to PE150. Upon filtering out raw-read adapter sequences and low-quality PE reads, a total of 638.73 Gb clean Hi-C reads were obtained, representing 62.23× coverage of the estimated genome size (Table S29. The clean Hi-C reads were initially truncated at putative Hi-C junctions and were subsequently aligned to the assembly results using the BWA. Only uniquely aligned read pairs with a mapping quality of >20 were retained for further analysis, while invalid read pairs, including the Da gluing-end and self-cycle, re-ligation, and dumped products, were filtered out using HiC-Pro v2.8.1,[108] resulting in 91.98% of valid interaction pairs. These pairs were then used for clustering or sorting and orienting scaffolds onto chromosomes through LACHESIS[109] (Table S30). The final pseudochromosomes were constructed manually. To assess the accuracy of the Hi-C assembly, an interaction heatmap of the Hi-C assembly chromosomes was generated.

Gene prediction and annotation

Three independent methods were employed to predict protein-coding genes: *de novo*, homology-based, and transcriptome-based prediction. For *de novo* gene prediction, GenScan v3.1,[110] Augustu v3.1,[111] Glimmer HMM v3.0.4,[112] Gene ID v1.4,[113] and SNAP v 2006-07-28 (http://homepage.mac.com/iankorf)[114] were utilized with default parameters. Homologous proteins from six known whole-genome sequences (*Arabidopsis*, *Ginkgo*, *G. montanum*, *P. abies*, *Populus trichocarpa*, and *P. taeda*) were aligned to the *C. lanceolata* genome sequence using GeMoMa v1.3.1[115] with default parameters. Transcriptome data assembly was performed using Hisat v2.0.4[116] and StringTie v1.2.3[117] with default parameters, followed by gene prediction using TransDecoder v2.0 (https://github.com/TransDecoder/TransDecoder) and GeneMarkS-T v5.1[118] with default parameters. PASA v2.0.2[119] with default parameters was employed to predict unigene sequences assembled from transcriptome data and full-length transcripts assembled from full-length transcriptome data. The results from all methods were combined and refined using EVM v1.1.1[120] and PASA v2.0.2. The completeness of the annotated *C. lanceolata* genome was assessed using BUSCO v5.8.2. Additionally, protein-coding gene annotation was performed using BLAST v2.2.31 (1e−5)[121] against the NR, EuKaryotic Orthologous Groups (KOG), GO, Translated European Molecular Biology Laboratory, and KEGG databases.

Identification of non-coding RNA, pseudogene, and repetitive sequences

Non-coding RNAs encompass RNA types with known functions, such as microRNAs (miRNAs), ribosomal RNA (rRNA), and transfer RNA (tRNA). The Rfam v12.1[152] and miRBase (v21) databases, along with Infernal v1.1.1 (http://infernal.janelia.org/)[122] (1e−5), were used to predict rRNA and miRNA, respectively. The tRNAs were predicted using tRNAscan-SE 1.3.1[123] with the option '-E -H'. Pseudogene homologue sequences were BLASTed using GenBlastA v1.0.4 (-e 1e−5),[124] and non-mature termination codes and frameshift mutations were identified using GeneWise v2.4.1 (-both -pseudo).[125]

Repeat sequences in *C. lanceolata* genome were predicted as follows: Initially, a *de novo* repeat library was constructed using LTR_FINDER v. 1.06 (http://tlife.fudan.edu.cn/ltr_finder/),[126] Repeat Scount v1.0.5, and PILER-DF v 2.4[127] with default parameters. The database was classified using PASTEClassifier v1.0[128] before being combined to build a new repeat database using the RepBase v19.06[153] (http://www.girinst.org/repbase). RepeatMasker v. 4.0.5 (http://www.repeatmasker.org/RepeatModeler/)[129] was used to align sequences and to screen repeats, including simple repeats, satellites, and low-complexity repeats, using the set parameter '-nolow -no_is -norna -engine wublast -qq -frag 20000'. The timing of LTR insertion was estimated using an LTR retriever.[130]

Building gene families with OrthoMCL

The amino acid and nucleotide sequences of 19 representative species were obtained from various sources: *Arabidopsis thaliana*, *Oryza sativa*, *Selaginella moellendorffii*, and *Physcomitrella patens* from Phytozome (https://phytozome.jgi.doe.gov/)[154]; *Cinnamomum kanehirae*, *P. taeda, Anthoceros angustus*, *G. montanum*, *W. mirabilis*, *C. panzhihuaensis*, and *T. wallichiana* from the National

CellPress
OPEN ACCESS

Center for Biotechnology Information (NCBI; https://www.ncbi.nlm.nih.gov/genome); *A. alba* and *P. abies* from the Plant Genome Integrative Explorer Resource (http://plantgenie.org/)[155]; Ginkgo from GigaDB[156]; *Amborella* from Ensembl plants; *Nymphaea tetragona* from Genome Warehouse (https://bigd.big.ac.cn/gwh/)[157]; and *Salvinia cucullata* and *Azolla filiculoides* from FernBase (www.fernbase.org).[158] Gene families or orthologous groups of these species and *C. lanceolata* were constructed using OrthoMCL v1.4 (http://orthomcl.org/orthomcl/).[131] In addition, KEGG and GO enrichment analyses were performed on the unique gene families identified in the *C. lanceolata* genome.

Phylogenetic reconstruction

Orthological analysis revealed the absence of single-copy gene families in 19 species. Therefore, we extracted genes from the pan-single-copy gene family (i.e., the single-copy gene family found in at least 50% of the species) to construct a phylogenetic tree. Initially, we identified 27 pan-single-copy gene families. Subsequently, BLASTP was employed to align multiple-copy gene families in a particular species with single-copy gene families in other species. The gene with the best alignment score was selected as the single-copy gene of the species. A total of 58 single-copy gene families were identified.

The amino acid sequences of these single-copy orthologues were aligned using MUSCLE v3.8.31 (http://www.drive5.com/muscle/).[132] Subsequently, the amino acid alignment was converted to nucleic acid multiple sequence alignment based on codon correspondence. After filtering the multiple sequence alignment results using Trimal,[133] phylogenetic trees were constructed using RAxML[134] via concatenation and ASTRAL methods based on nucleic acid and amino acid sequences, respectively. For nucleic acid and amino acid sequences, the parameter was set to -m GTRGAMMA and -m PROTGAMMAJTT, respectively.

Additionally, to mitigate LBA artifacts, we selected the first two bases of the codon for nucleotide construction in concatenated and astral trees. Subsequently, we constructed the Bayesian phylogenetic tree using PhyloBayes with the ‘cat model’ to effectively avoid LBA artifacts.

Estimation of divergence time

The divergence time of each tree node was inferred using the MCMCtree tool from the PAML4.9 package[135] The analysis utilized a correlated molecular clock and the JC69 model, with other settings left as default). Nucleic acid replacement was modeled using the GTR model and the molecular clock followed an independent rate model. The Markov Chain Monte Carlo (MCMC) process included 100 000 burn-in iterations, followed by 1 000 000 sampling iterations, with one sample collected every 100 iterations). The phylogeny was calibrated using various fossil records or molecular divergence estimates, with soft bounds placed at the split nodes of several key species pairs, including *G. montanum*–Ginkgo (230–282 Ma), *A. thaliana–G. montanum* (289–330 Ma), *A. angustus–G. montanum* (392–422 Ma), *and P. patens–G. montanum* (450–514 Ma).

Gene family expansion and contraction

Based on the phylogenetic tree, gene family expansion and loss rates were inferred using CAFÉ 4.2 (https://github. com/hahnlab/CAFE).[52] Functional enrichment analysis was conducted on genes belonging to significantly expanded and contracted gene families in *C. lanceolata*. However, owing to the limited number of genes showing significant contraction and lack of enriched functions, the functional annotation results of these genes are listed in Tables S11 and S12.

Analysis of whole-genome duplications (WGDs) and intra- and inter-genomic comparisons

We used synonymous substitutions per synonymous site (K_s) distribution analysis to identify WGD events in the gymnosperm genomes with the package of wgd v1.1.2.[136] Simply speaking, Diamond v2.1.11[137] was employed for self-alignment of the protein sequences of the genomes of these species, with subsequent extraction of the mutual optimal alignment from the results. MCL 22–282[138] was executed to cluster all alignment results into gene families. Codeml in the PAML package[135] was used to calculate the K_s values between gene pairs in each gene family. Additionally, i-adhore v3.0.01[139] (default parameter) was utilized to identify collinear segments within paranomes and to filter out all paralogous gene pairs that not located in colinear segments. Finally, the K_s distribution of all paralogous gene pairs, and of all anchor pairs, were obtained by the internal function of wgd v1.1.2.

For intra- and inter-genomic comparisons, JCVI[140] with default parameters was employed to conduct a collinear analysis and visualization of the gymnosperms’ genomes, including *G. biloba*, *C. panzhihuaensis*, *C. lanceolata*, *T. wallichiana*, *W. mirabilis*, and *G. montanum*.

Then, we performed the phylo-K_s analysis to place the identified WGD events in *G. biloba*, *C. panzhihuaensis*, and *W. mirabilis* on a species tree with branch lengths in the K_s unit.[46] Concisely, OrthoFinder v2.3.3[141] was used to infer orthologous gene families using default parameters for 10 selected seed plants genomes, including *Selaginella moellendorffii, Adiantum capillus, Azolla filiculoides, Cunninghamia lanceolata, Taxus wallichiana, Gnetum montanum, Welwitschia mirabilis, Cycas panzhihuaensis, Ginkgo_biloba,* and *Amborella trichopoda.* All single-copy orthologous amino acid gene families were obtained from the output of OrthoFinder. PRANK[142] with default parameters was used to perform multiple sequences alignment (MSA) for each gene family with protein sequences. Then, trimAl[143] with ‘-backtrans -automated1’ parameters were employed to map each nucleotide data to corresponding amino acid MSA matrix and simultaneously trim it to obtain final codon-level MSA matrix for each single-copy gene families. All codon-level MSAs were concatenated followed by a Codeml analysis to infer the branch lengths in the K_s unit under the free ratio model.

For the statistical gene tree – species tree reconciliation, WHALE[50] was used to test the validity of the previously proposed ancient WGD events.[159,160] OrthoFinder v2.3.3[141] was used to infer orthologous gene families using default parameters for 11 selected seed plants genomes, including *Selaginella moellendorffii, Adiantum capillus, Azolla filiculoides, Cunninghamia lanceolata, Taxus wallichiana, Picea abies, Pinus taeda, Amborella trichopoda, Nymphaea colorata, Arabidopsis thaliana, Oryza sativa*. Gene families lacking genes from both clades at the root or exceeding twice the median of the square root of the family size based on a Poisson outlier criterion, were filtered out. An amino acid multiple sequence alignment (MSA) for each gene family was obtained using PRANK.[142] The resulting MSAs were then used as input for Markov Chain Monte Carlo (MCMC) analysis in mrbayes v.3.2.6[49] to sample from the posterior probability distribution. 'Aamodelpr' was set as 'fixed (LG)', and the rates were set as gamma-distributed variations approximated using four categories. The sampling frequency was set to 100, and 1 100 000 generations were run to obtain 11 000 posterior samples. Subsequently, ALEobserve[144] from ALE v1.0 was used to construct the conditional clade distribution (CCD) containing marginal clade frequencies, with a burn-in of 1000 based on 11 000 posterior samples for each gene family. The topology of the species tree was set as shown in Figure 2B, and divergence times were retrieved from TimeTree.[145]

The duplication-loss DL + WGD model under the critical and relaxed branch-specific model was employed to infer corresponding WGD retention rates (q) with the three hypothetical WGDs under the using Bayesian scheme, with WGD1 before the divergence of the seed plants, WGD2 before the divergence of gymnosperms, and WGD3 before the divergence of angiosperms. In the critical branch-specific DL + WGD model, several priors were specified, with η representing the parameter of the geometric prior distribution on the number of genes at the root set to follow a truncated univariate Beta distribution with shape parameters (3,1) within the interval [0.01, 0.99]. The prior r, denoting the mean of the branch rate distribution was set to follow a flat distribution, while the prior σ, representing the deviation of the branch rate distribution, followed an exponential distribution within a scale of 0.1. Additionally, λ, denoting the duplication rate of each branch, was set to follow a multivariate normal distribution for each branch, with the loss rate μ equal to λ.

Conversely, in the relaxed branch-specific model, λ and μ were considered independent, with the rate variation parameter τ set to follow an exponential distribution with a scale of 1. In the model estimating the branch-specific duplication and loss rates, λ and μ were set to follow a normal distribution with a mean of 0 and a standard deviation of 5 in log scale for each branch, independently. All branch lengths were set to 1, and no WGD nodes were considered. The Bayes Factor was calculated using the "bfact.jl" script within the public Github repository of WHALE to measure the strength of evidence favoring the assumed WGD models using the Savage-Dickey density ratio.

Transcriptomic data and analysis

The materials used for the transcriptome sequencing were obtained from four sources. Firstly, young tissues from both vegetative organs (leaf, phloem, stem, and root) and reproductive organs (homozygous female gametophytes, male cones, female cones, seed scales, and bract scales) were obtained as the initial genome sequencing material. Secondly, germinating and astringent seeds were collected at four distinct time points (105, 115, 125, and 135 days) (Method S1).

For library construction, a total of 1.5 μg RNA was prepared, and libraries were constructed using the NEBNextR UltraTM Directional RNA Library Prep Kit for IlluminaR (NEB). Index codes were incorporated to attribute sequences to each sample. The index-coded samples were then clustered using an acBot Cluster Generation System with a TruSeq PE Cluster Kitv3-cBot-HS (Illumina) according to the manufacturer's instructions. Following cluster generation, the libraries were sequenced on an Illumina HiSeq platform, resulting in the generation of paired-end reads. Raw data in FASTQ format were first processed using in-house Perl scripts. This step involved obtaining clean data by eliminating reads containing adapters, poly-N, and low-quality reads from the raw data. StringTie (1.3.1) was used to calculate the Fragments Per Kilobase of exon model per Million mapped fragments (FPKMs) of coding genes in each sample. Gene fragments per kilobase of transcript per million mapped reads (FPKMs) were computed by aggregating the FPKMs of the transcripts in each gene group.

Demographic history analysis

Considering the extensive artificial introduction and transfer of germplasm resources across *C. lanceolata* provinces in China over decades, the genetic structure of commercial forests across different regions may have been strongly influenced by artificial intervention; thus, they cannot accurately represent the phylogeographical characteristics of *C. lanceolata*. Therefore, we focused on identifying communities of *C. lanceolata* in natural forests located within their distribution areas. We specifically targeted individual plants aged over 50 years as samples. A total of 128 individuals from 10 natural *C. lanceolata* populations were selected to represent the majority of known *C. lanceolata* localities (Table S22). DNA was extracted from young leaves of each individual collected for SLAF sequencing. Library construction followed the methodology outlined by Sun et al.[161] Genomic DNA was digested into fragments of approximately 330 bp using the EcoRV-HF restriction enzyme. Thereafter, fragment ends were repaired and ligated with indexed paired-end adapters to obtain adapter-modified ends.

PCR was performed to amplify the target fragments, and fragments of appropriate sizes were selected based on agarose gel electrophoresis. Finally, the pooled libraries were sequenced using an Illumina HiSeq X-ten paired-end sequencer, according to the manufacturer's protocol (Illumina, San Diego, CA, USA).

The reads of each sample were aligned to the reference genome using Bwa software. Reads in which both ends were aligned with the reference genome successfully and uniquely were considered reliable and were used to define SLAF tags. We used GATK v3.8 to obtain SNPs based on the corresponding genome-wide SLAF tags. A total of 3,419,532 genome-wide SNPs were obtained, and

187,611 genome-wide SNPs were quality-filtered with a minor allele frequency of >0.05, and no more than 20% missing data. Finally, we performed population evolution analysis.

A neighbour-joining tree was constructed using MEGAX[146] and a p-distance model with genome-wide SNPs. A bootstrap consensus tree was constructed using 1000 replicates. Principal component analysis (PCA) of genome-wide SNPs was performed using EIGENSOFT software version 6.0, and the first three eigenvectors were plotted in two or three dimensions. The software ADMIXTURE v 1.3.0,[147] which is based on the likelihood model embedded in STRUCTURE software, was applied to infer historical lineages that show clusters of similar genotypes. The membership of each genotype was run for a range of genetic clusters from K = 1–10 using the admixture model.

MADS-box gene family analysis

HMM profiles of MADS (PF00319) genes were obtained from Pfam (https://pfam.xfam.org/).[162] The MADS-box candidate protein was searched separately using HMMER 3.2.1 (http://hmmer.org)(with default parameters) and BLASTP (E-value of e^{-5}). Subsequently, the domains of all MADS-box candidate gene sequences were identified using SMART (http://smart. embl-heidelberg.de/).[148] MADS-box classification was based on sequence similarity searches of MADS-box genes identified in *Arabidopsis* and *Amborella*. All candidate MADS-box genes were aligned using MAFFT.[149] A phylogenetic tree was constructed using FastTree v2.1.10[150] and edited using Figtree v1.4.4(http://tree.bio.ed.ac.uk/software/figtree/).

QUANTIFICATION AND STATISTICAL ANALYSIS

All details of the statistics applied in this study are provided alongside the respective analysis in the method details section.

Cell Reports, Volume *45*

Supplemental information

Cunninghamia lanceolata genome illuminates the evolutionary dynamics of gymnosperms

Si-Zu Lin, Yu Chen, Chao Wu, Wei-Hong Sun, Zhen Li, Heng-Chi Chen, Jie-Yu Wang, Chang-Mian Ji, Shu-Bin Li, Zhi-Wen Wang, Wen-Chieh Tsai, Xiang-Qing Ma, Si-Ren Lan, Fei-Ping Zhang, Ya-Cong Xie, Lei Yao, Yan Zhang, Meng-Meng Lü, Jia-Jun Zhang, Di-Yang Zhang, Yi-Quan Ye, Xia Yu, Shan-Shan Xu, Zhi-Hui Ma, Guo-Chang Ding, Guang-Qiu Cao, Zong-Ming He, Peng-Fei Wu, Kai-Min Lin, Ai-Qin Liu, Yan-Qing Lin, Shao-Ning Ruan, Bao Liu, Shi-Jiang Cao, Li-Li Zhou, Ming Li, Peng Shuai, Xiao-Long Hou, Yi-Han Wu, Nuo Li, Sheng Xiong, Yang Hao, Zhuang Zhou, Xue-Die Liu, Dan-Dan Zuo, Jia Li, Pei Wang, Jian Zhang, Ding-Kun Liu, Gui-Zhen Chen, Jie Huang, Ming-Zhong Huang, Yuan-Yuan Li, Qin-Yao Zheng, Xue-Wei Zhao, Xiang Zhao, Wen-Ying Zhong, Xue-Wen Zhang, Zheng-Bao Xia, Ying Yu, Zhi-Wei Liu, Hong-Kun Zheng, Ray Ming, Yves Van de Peer, and Zhong-Jian Liu

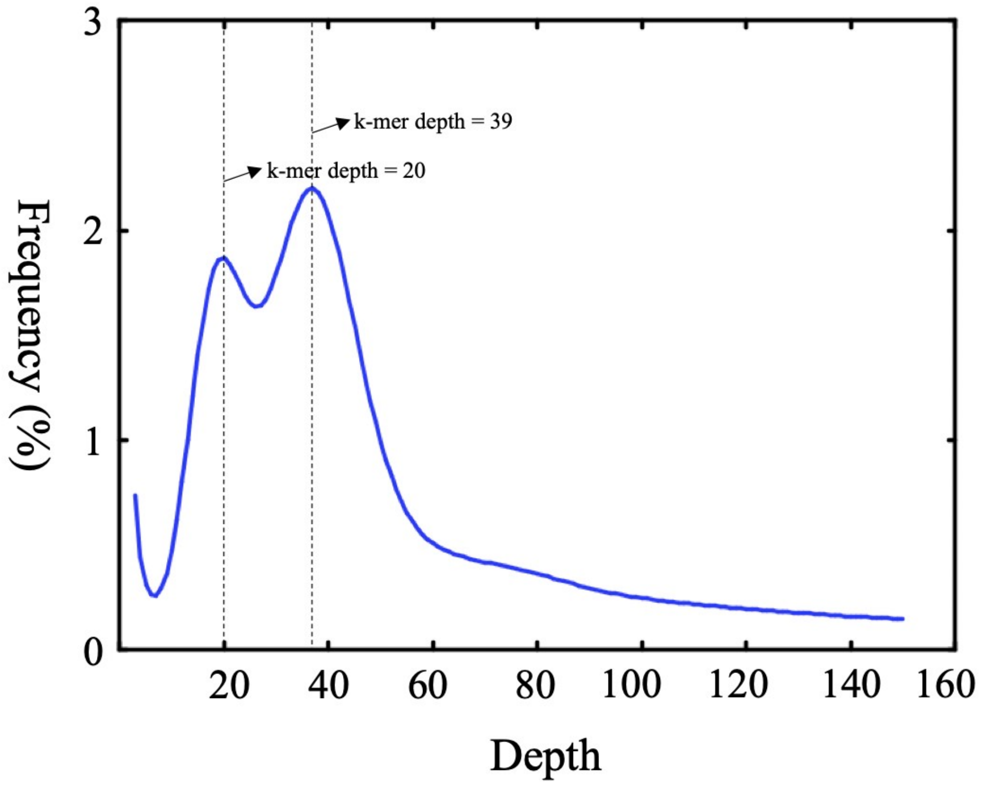

Figure S1. Genome size and heterozygosity of *C. lanceolata* estimation using K-mer distribution.

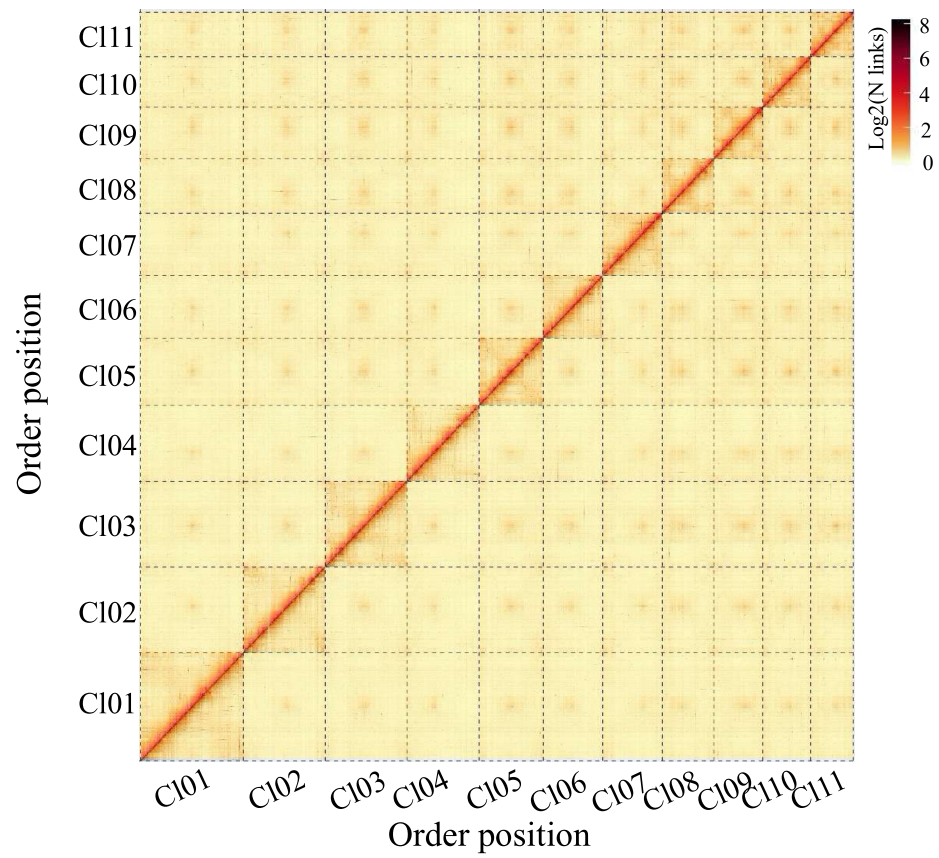

Figure S2. Hi-C interaction heatmap for *C. lanceolata* genome showing interactions among eleven chromosomes. Darker red pixels denote higher contact probabilities. Most interactions were observed within the chromosomes.

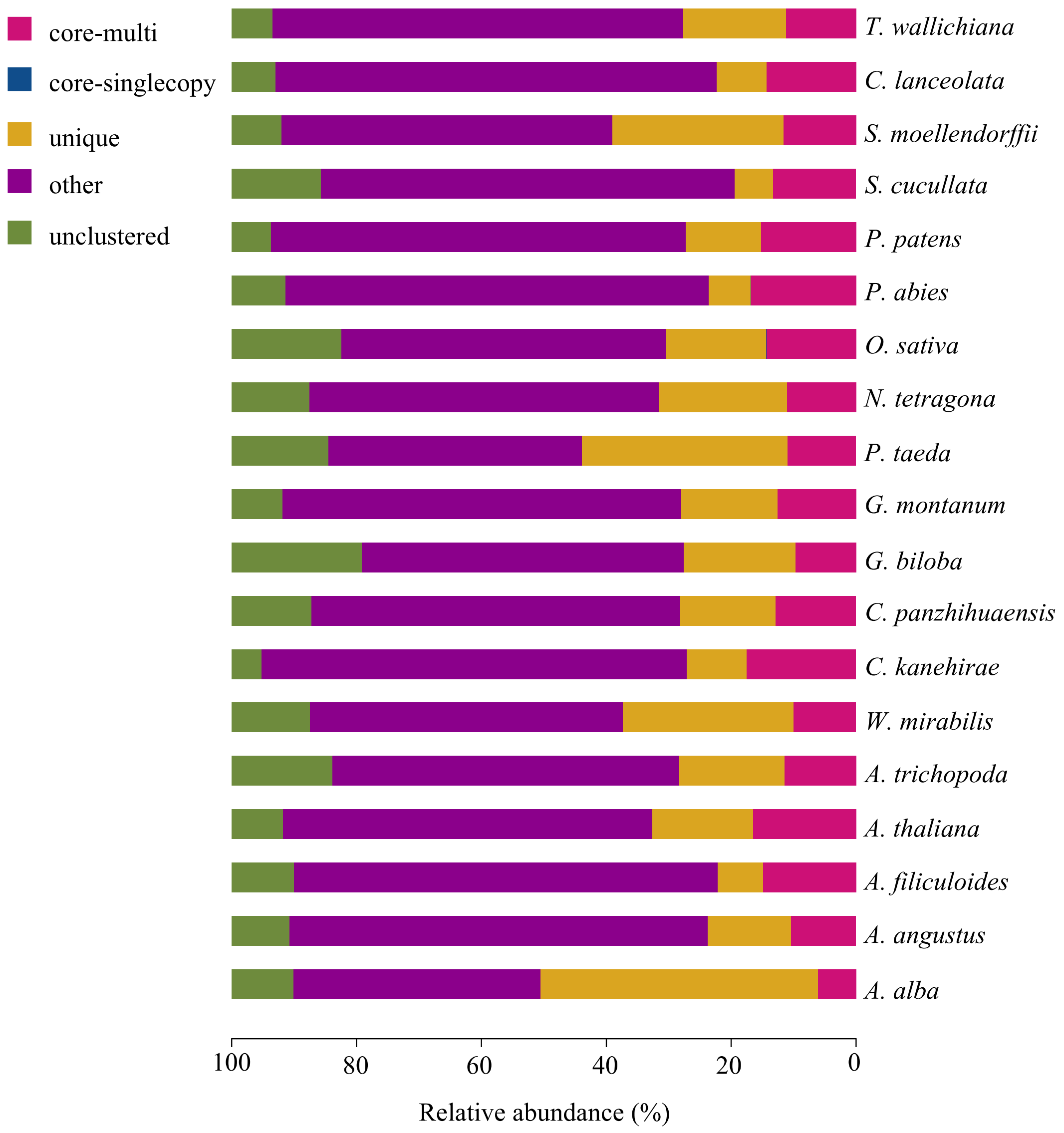

Figure S3. Orthologous genes in *C. lanceolata* and other species.

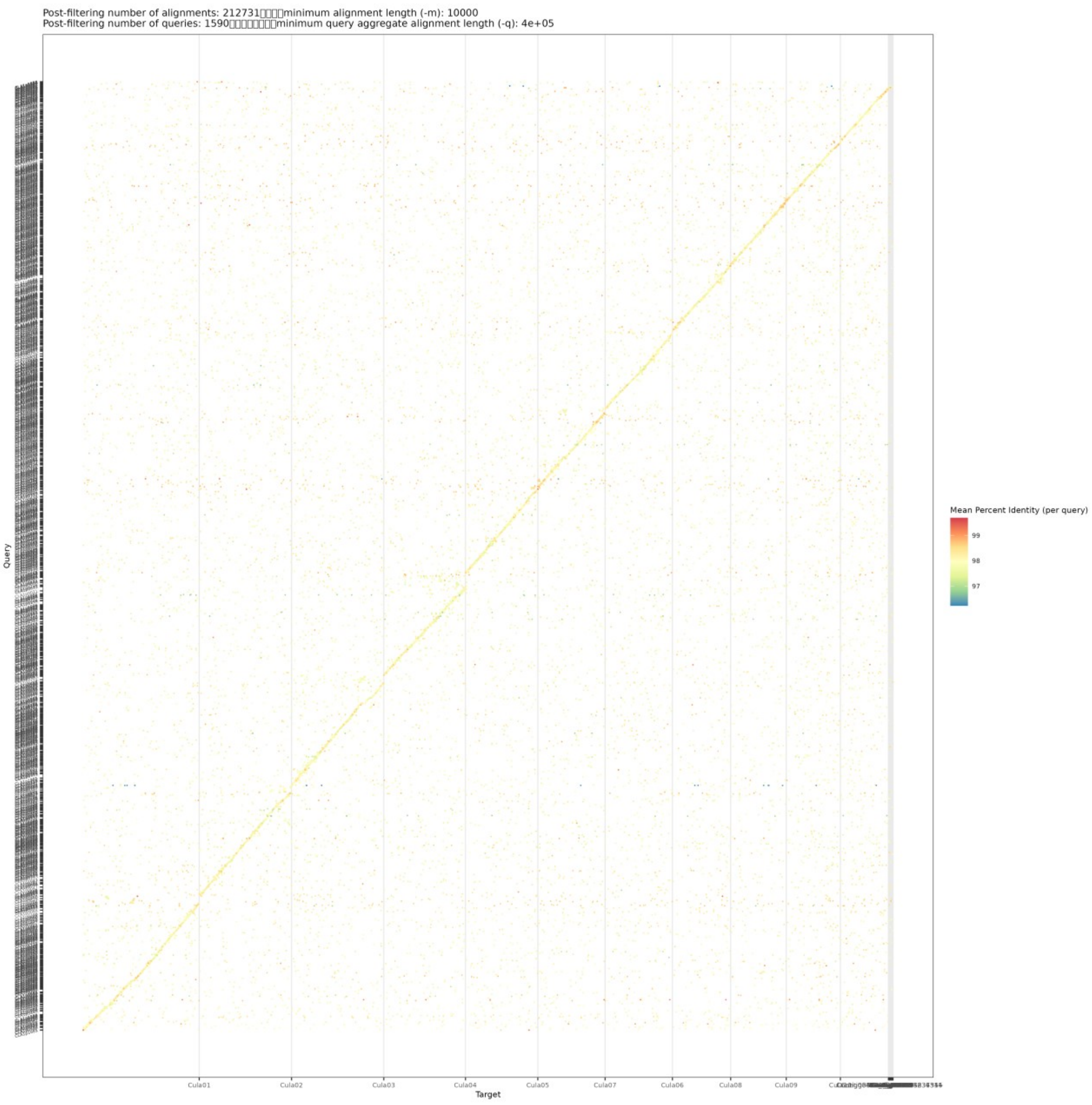

Figure S4. Visualization of the overage of the assembly map by Shirasawa et al. on chromosomal assembly in this paper.

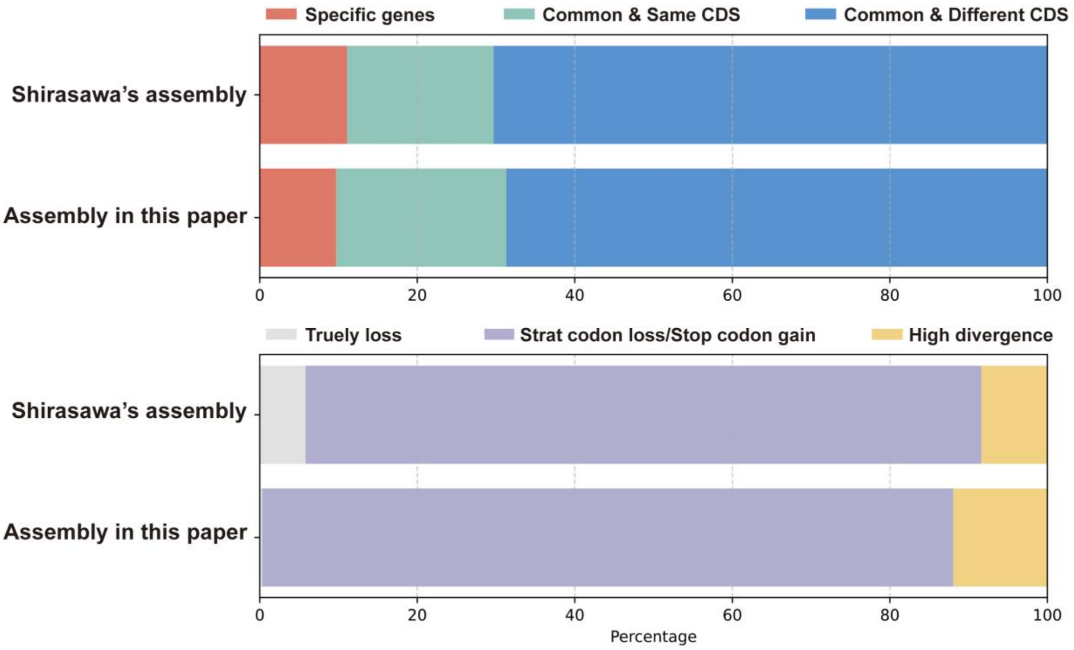

Figure S5. Comparison of annotation results between the two *C. lanceolata* genomes. Specific genes represents unique genes in the Shirasawa's assembly. Common genes represents the shared genes between the to genomes. Truly loss represents the genes completely lost in assembly in this paper. Star codon loss/Stop codon gain represents the mutations occurred in the CDS regions of genes in assembly in this paper, resulting in start codon deletion or premature stop codon acquisition with CDS sequences. High divergence represents orthologous genes exist in both assemblies but with substantial sequence divergence, with identity and coverage both lower than 80%.

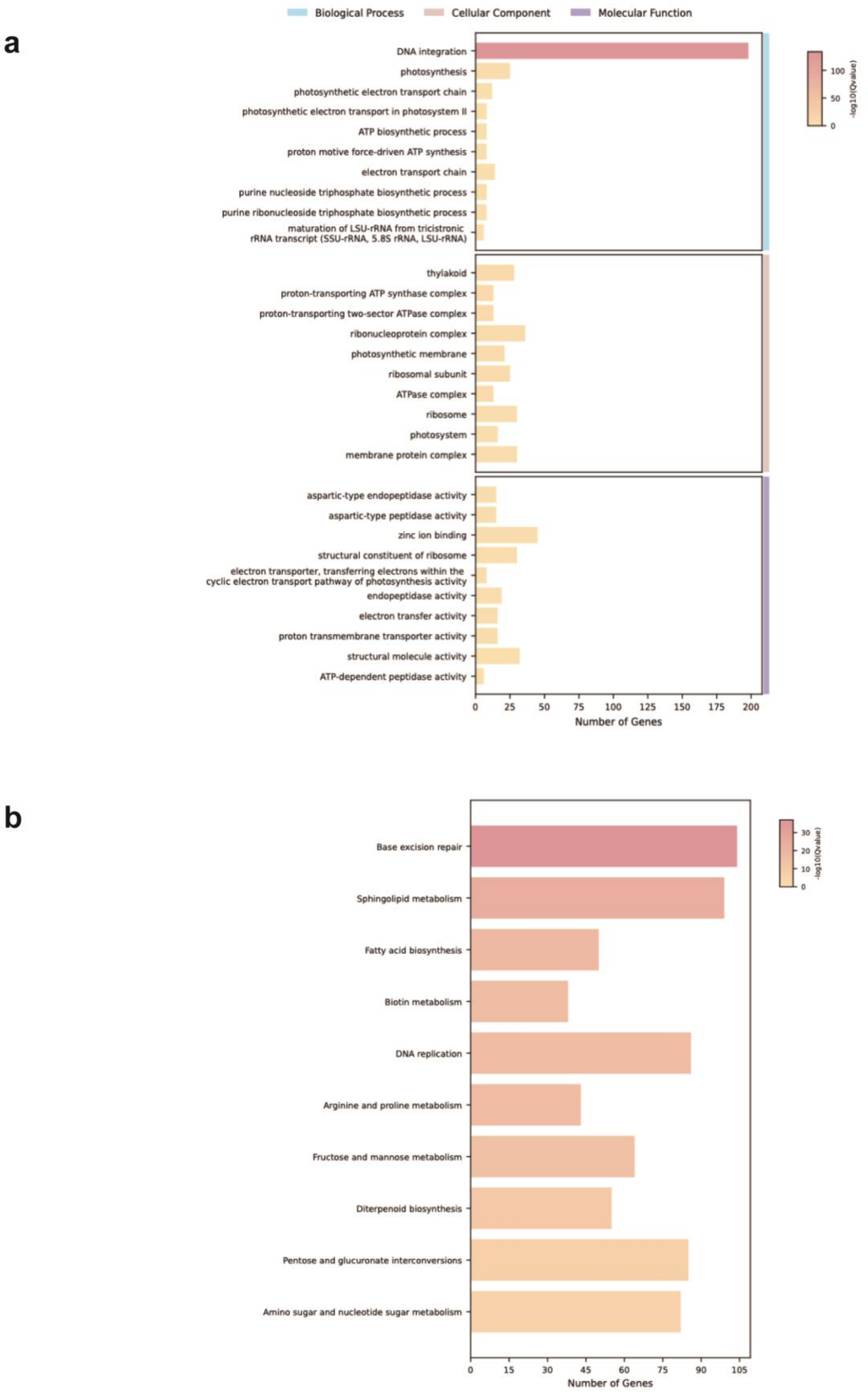

Figure S6. Enrichment analysis of specific genes. (a) GO enrichment. (**b)** KEGG enrichment.

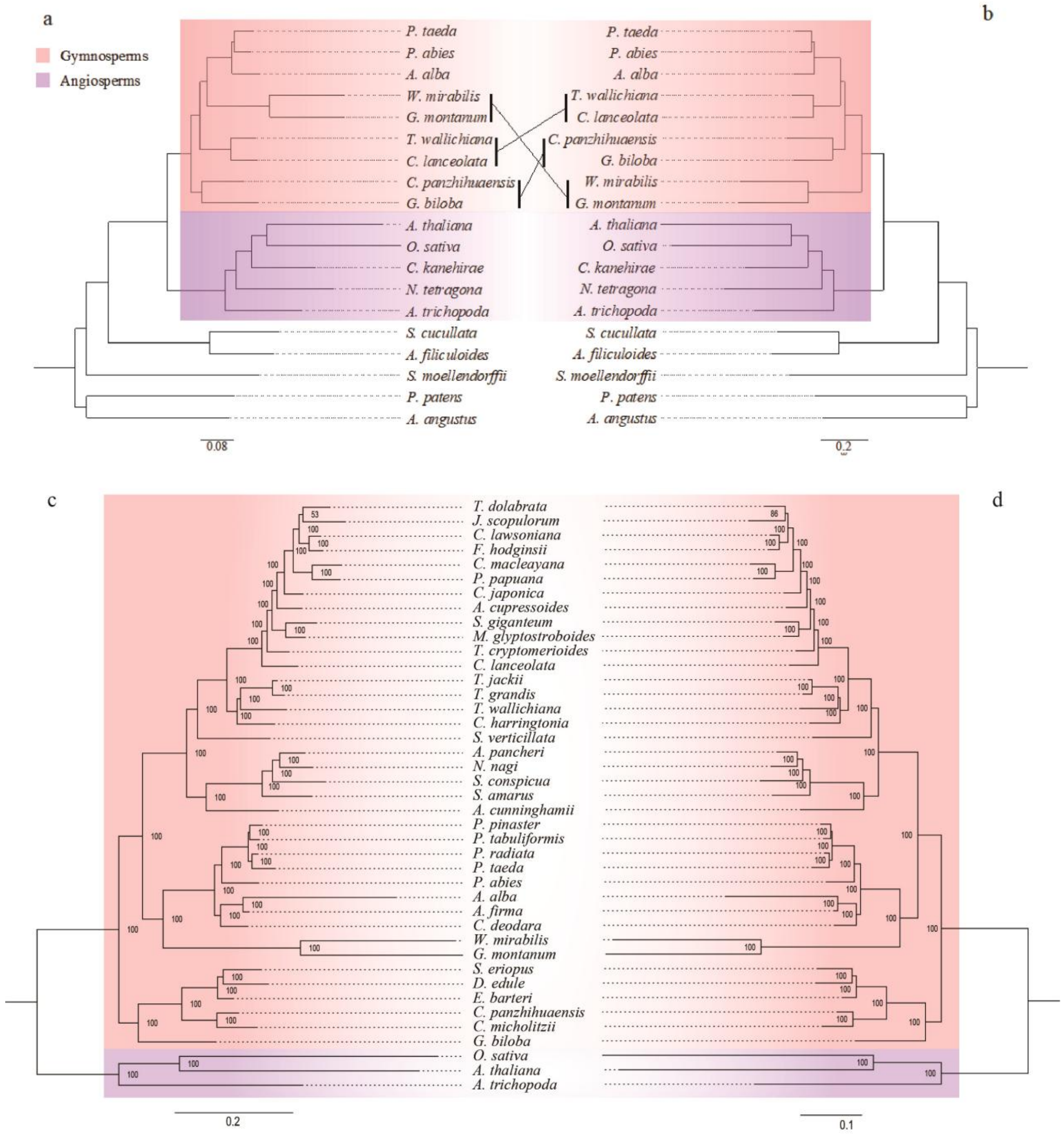

Figure S7. Phylogenetic tree constructed by different methods based on single copy-genes. **(a)** The concatenated tree based on amino acid, concatenated tree constructed by the first and two codons, and the Bayesian tree. **(b)** The concatenated tree based on nucleotides, ASTRAL tree, andASTRAL, tree based on constructed by the first and two codons. The topological structure ofconcatenated tree based on amino acid, concatenated tree based on constructed by the first and twocodons, and the Bayesian tree are the same, while the topological structure of concatenated treebased on nucleotides, ASTRAL, tree, and ASTRAl, tree based on constructed by the first and twocodons are same. **(c)** The concatenated tree based on amino acid sequences from genomes and transcriptomes of 32 species.**(d)** The concatenated tree based on nucleotide sequences from genomes and transcriptomes of 32 species.

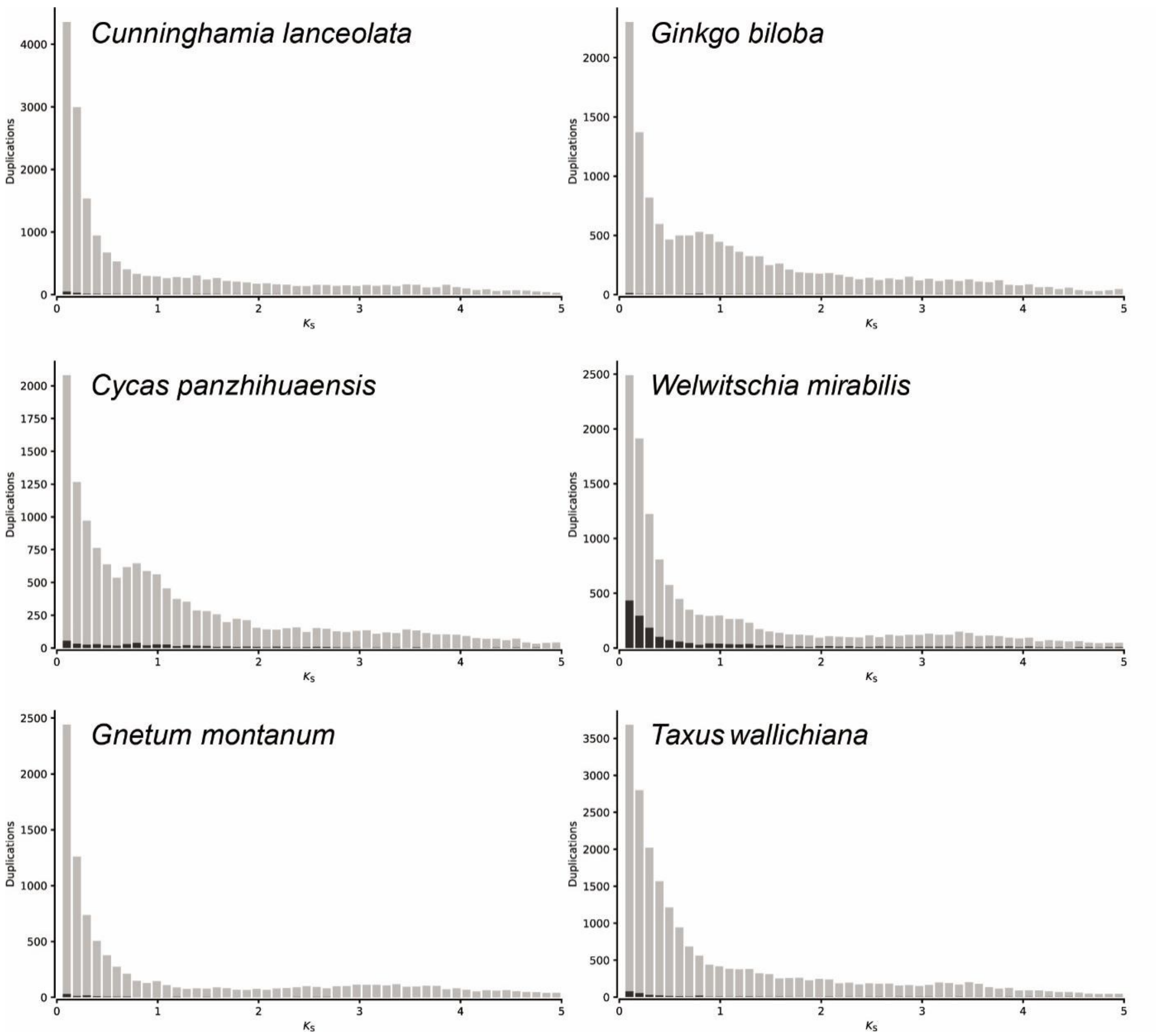

Figure S8. Distribution of synonymous substitutions per synonymous site (K_S) for paralogous gene pairs (gray bars) and syntenic retained paralogous pairs (black bars) in six gymnosperm species.

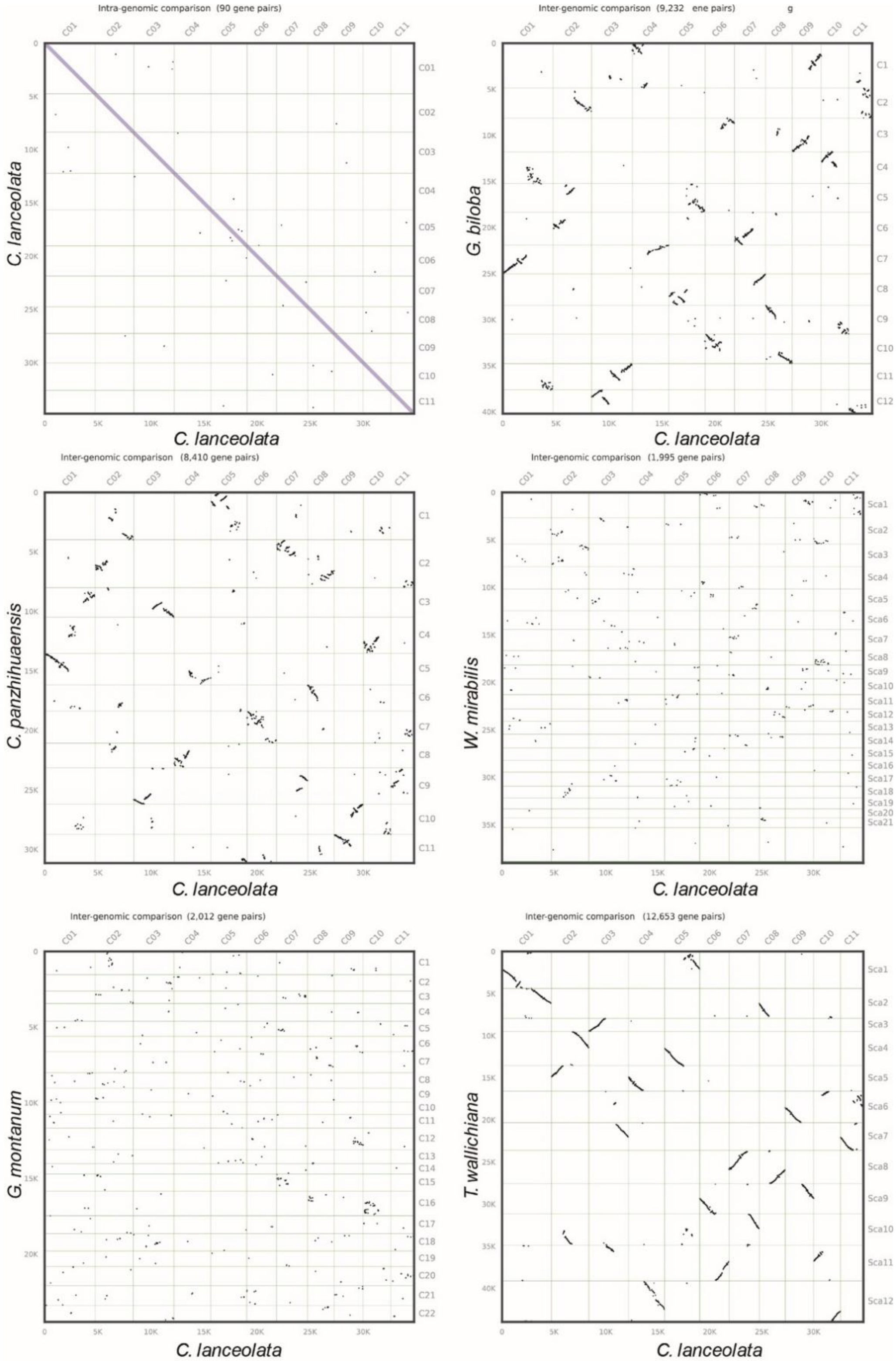

Figure S9. **Colinear dot plots for intra-genomic comparison within *C. lanceolata* genome and inter-genomic comparisons between *C. lanceolata* and each of five other gymnosperm genomes** (i.e. *G. biloba*, *C. panzhihuaensis*, *W. mirabilis*, *G. montanum*, and *T. wallichiana*)

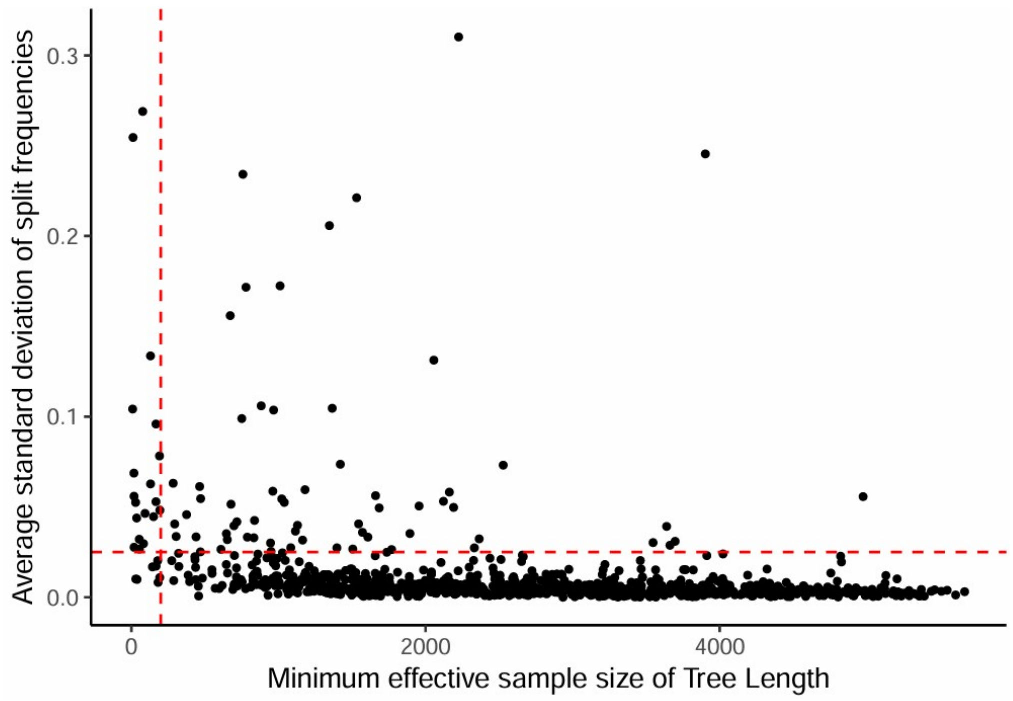

Figure S10. **Minimum effective sample size of Tree Length and the average standard deviation of split frequencies for the 1,000 randomly selected gene families.** The gene families with the minimum effective sample size of tree length > 200 (the red dashed line in vertical) and the average standard deviation of split frequencies < 0.025 (the red dashed line in horizontal) were selected for the WHALE analysis.

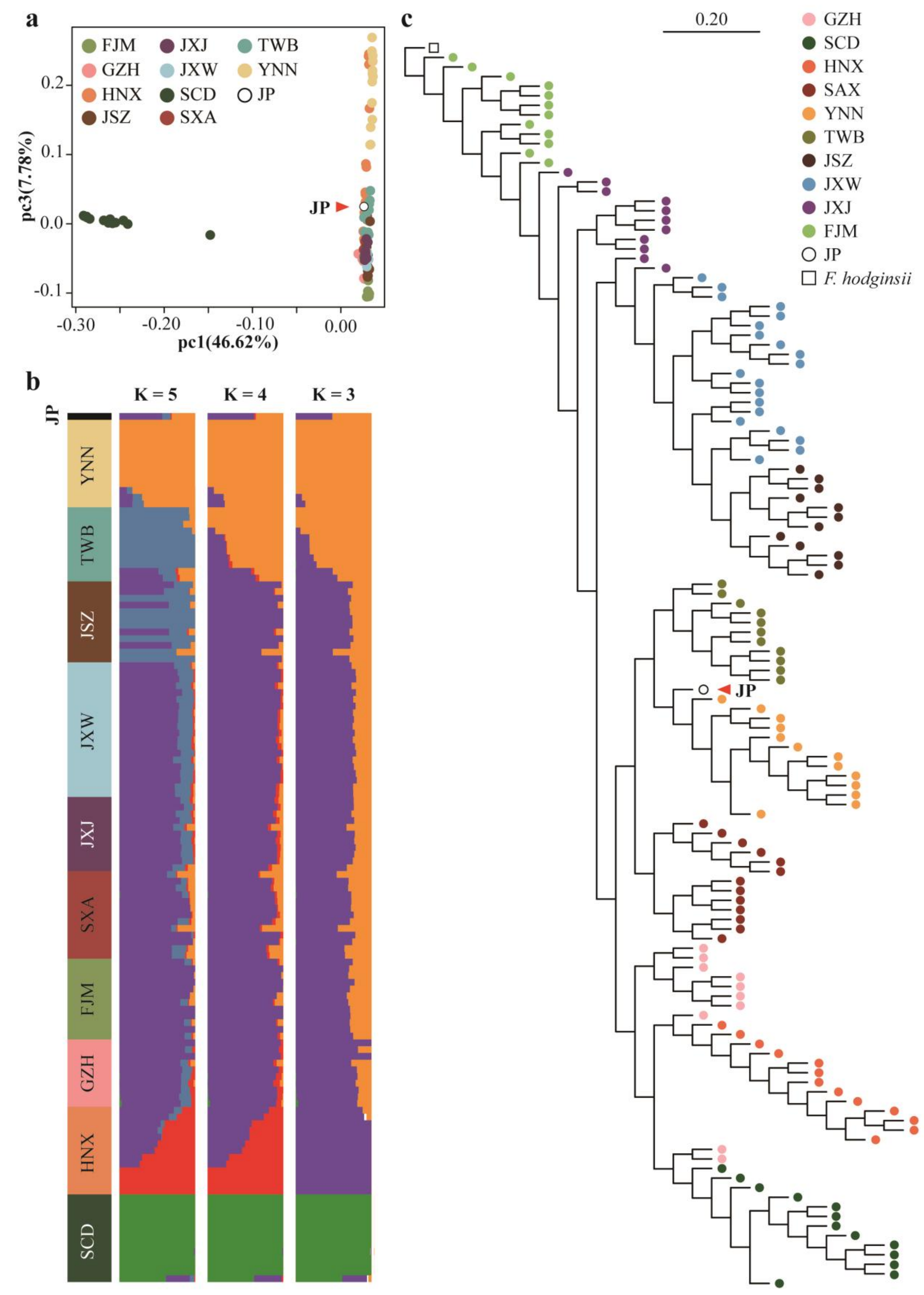

Figure S11. **Population evolutionary analysis of *C. lanceolata* with the inclusion of Japanese-grown *C. lanceolata* genome reported by Shirasawa et al..**
Consistent results from PCA **(a)** STRUCTURE analysis **(b)** and phylogenetic tree reconstruction **(c)** demonstrate that the genetic structure of Japanese-introduced *C. lanceolata* is highly similar to those of the TWB and YNN populations.

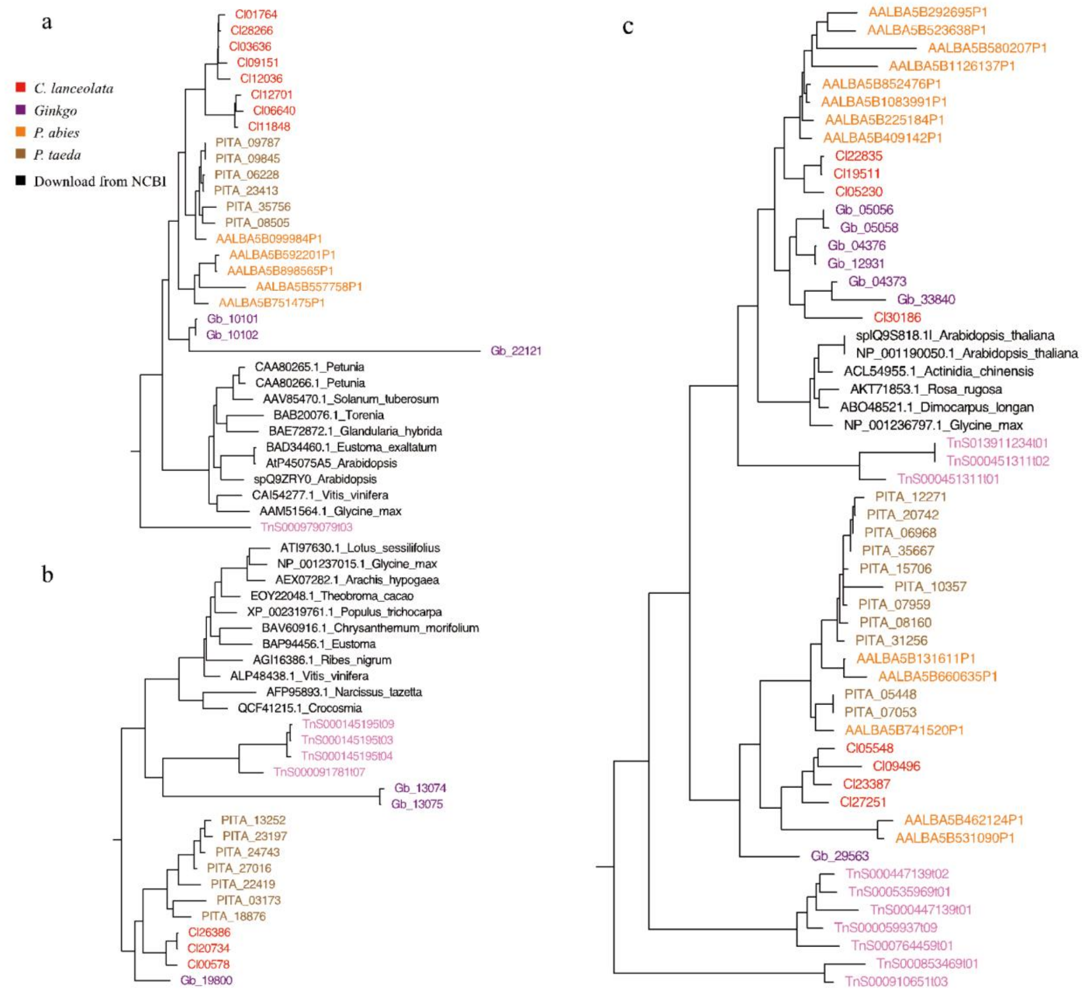

Figure S12. Phylogenetic tree of flavonoid 3',5'-hydroxylase (*F3′5′H*), flavonoid 3'-hydroxylase (*F3′H*), and flavanone-3-hydroxylase (*F3H*) from seed plants.(a) Flavonoid 3',5'-hydroxylase (*F3′5′H*). **(b)** Flavonoid 3'-hydroxylase (*F3′H*). **(c)** Flavanone-3-hydroxylase (*F3H*).

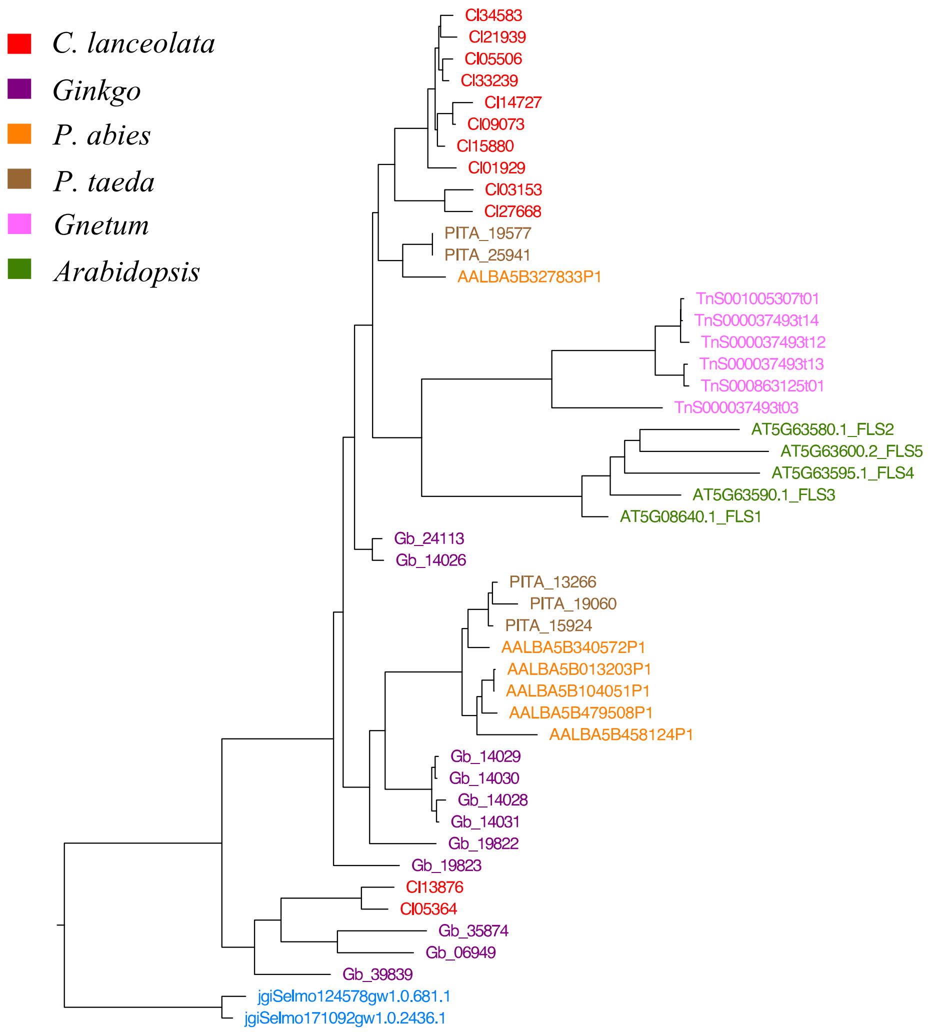

Figure S13. Phylogenetic relationships of flavonol synthase (*FLS*) gene. Two genes, *jgiSelmo124578gw1.0.681* and *jgiSelmo171092w1.0.2436.1*, from *Selaginella tamariscina* as outer group.

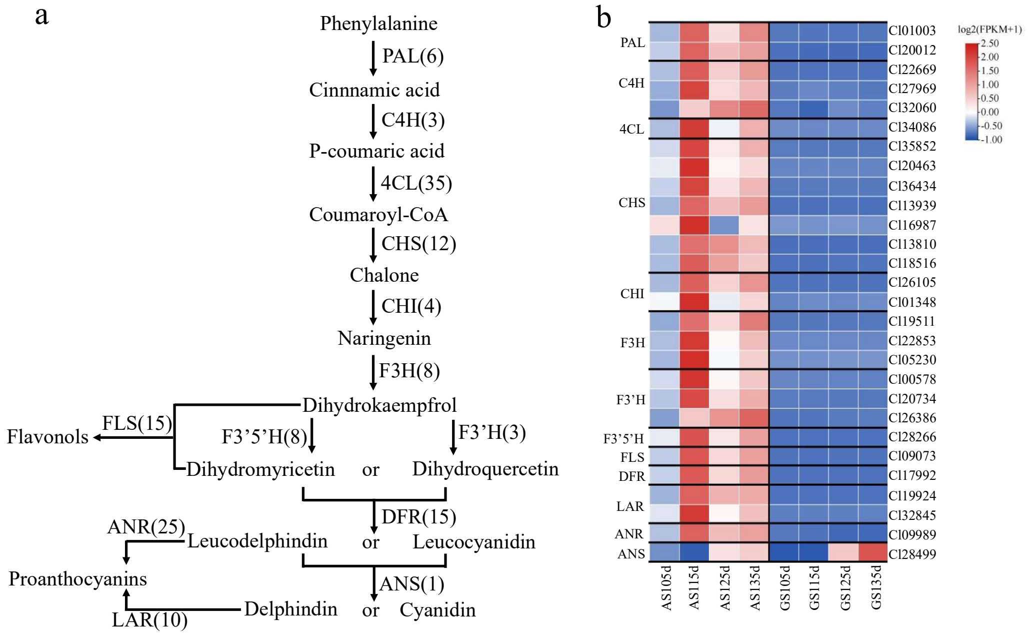

Figure S14. Expression of flavonoid synthesis related genes in astringent seeds (AS) and germinating seeds (GS) during different developmental stage. (a) Synthesis pathways of flavonoids. **(b)** The expression patterns of genes related to flavonoid synthesis in astringent seeds and germinated seeds at different stages. (see the full name of genes in Table S23)

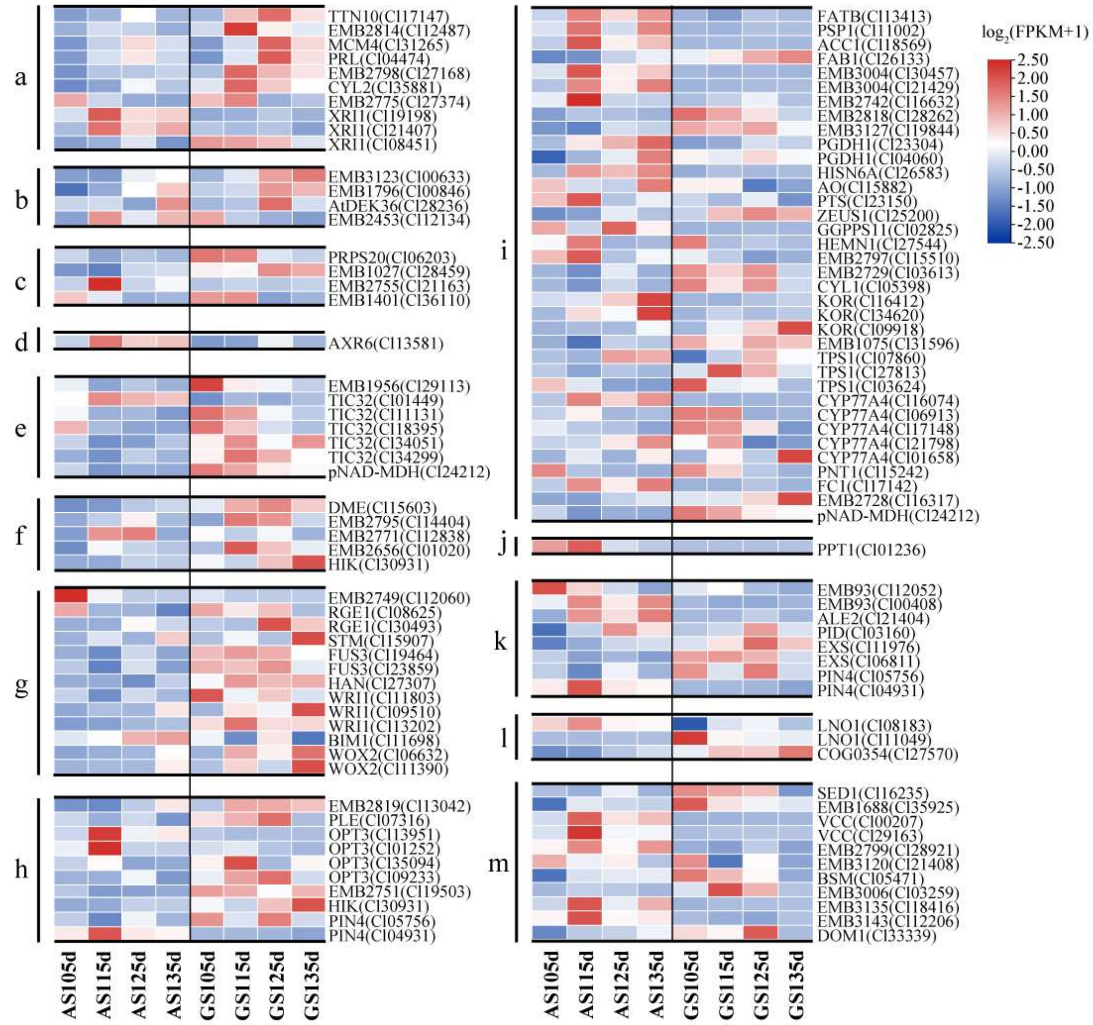

Figure S15. Expression pattern of 109 differential embryo-defective genes (*EMBs*) in astringent seeds (AS) and germinating seeds (GS) during four developmental stages. The letters on the left of the heat map represent the functional classification of the gene, the right side is the gene name, and the *C. lanceolata* gene ID is in parentheses. (**a**) DNA synthesis/repair.(**b**) DNA synthesis/modification. **(c)** Protein synthesis. (**d**) Protein degradation.(**e**) Protein modification/transport. **(f)** Chromosome dynamics. **(g)** Transcriptional regulation.**(h)** Cell structure. **(i)** metabolism. (**j**) Energy electron. (**k**) Signaling and regulatory pathways. (**l**) other (miscellaneous). (**m**) Uncertain/unknown.

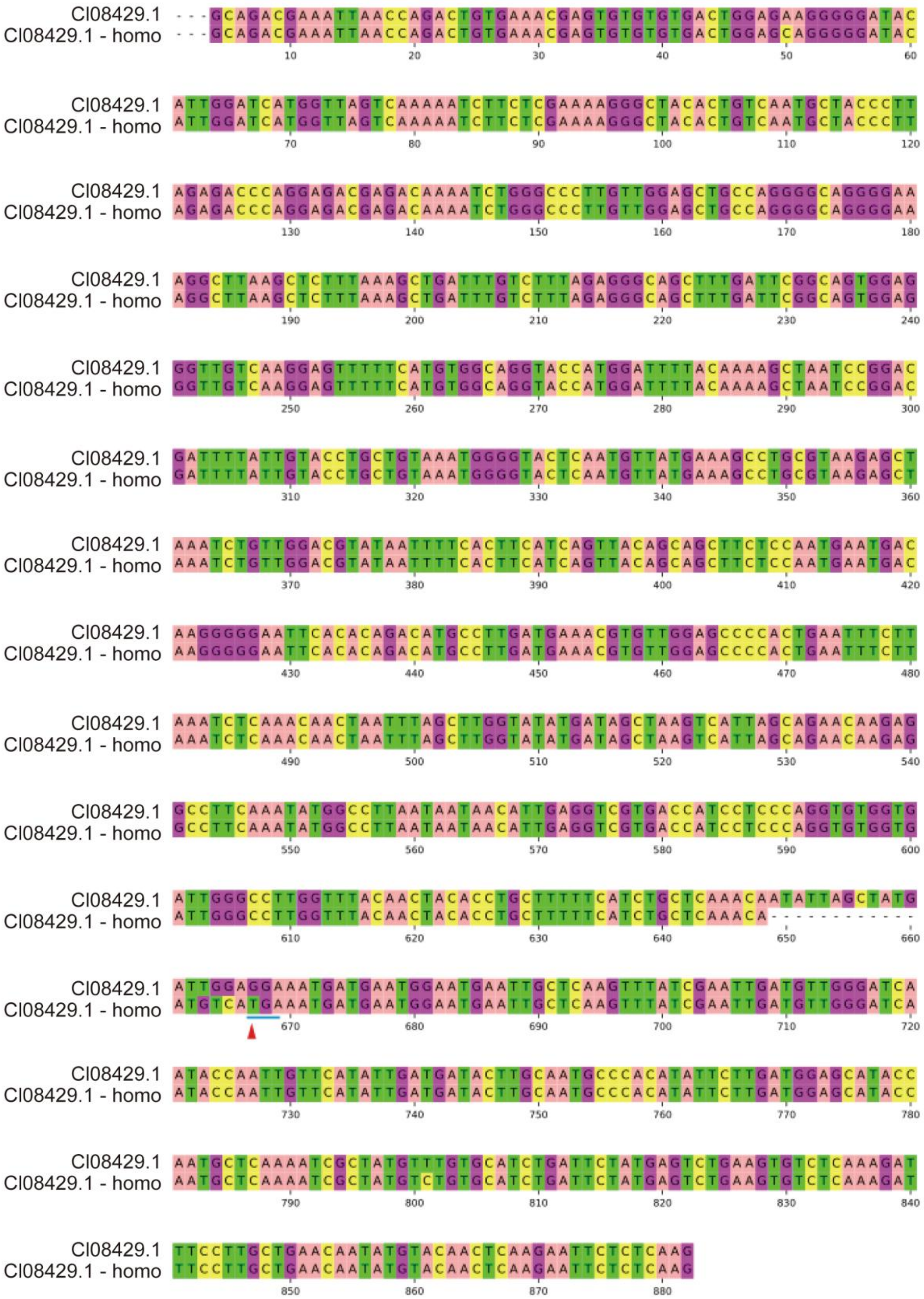

Figure S16. CDS alignment between Cl08429.1 and its homologous gene Cl08429.1-homo in the Shirasawa et al. assembly.

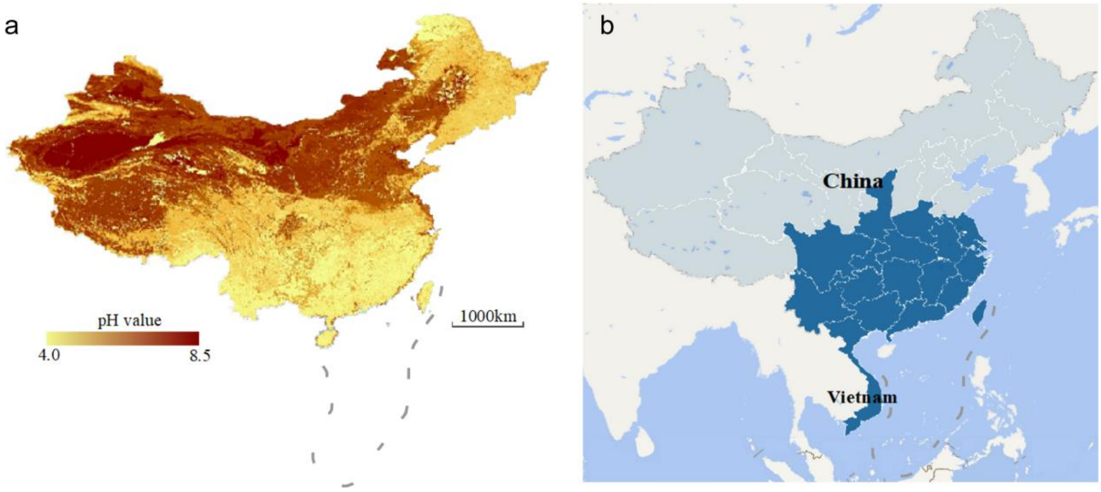

Figure S17. The distribution of *C. lanceolata* coincides with the distribution of acid red soil. (**a**) China's acid soil distribution map. Data sourced from the National Soil Information Service Platform of China (http://www.soilinfo.cn) (**b**) *C. lanceolata* distribution (dark blue) map.

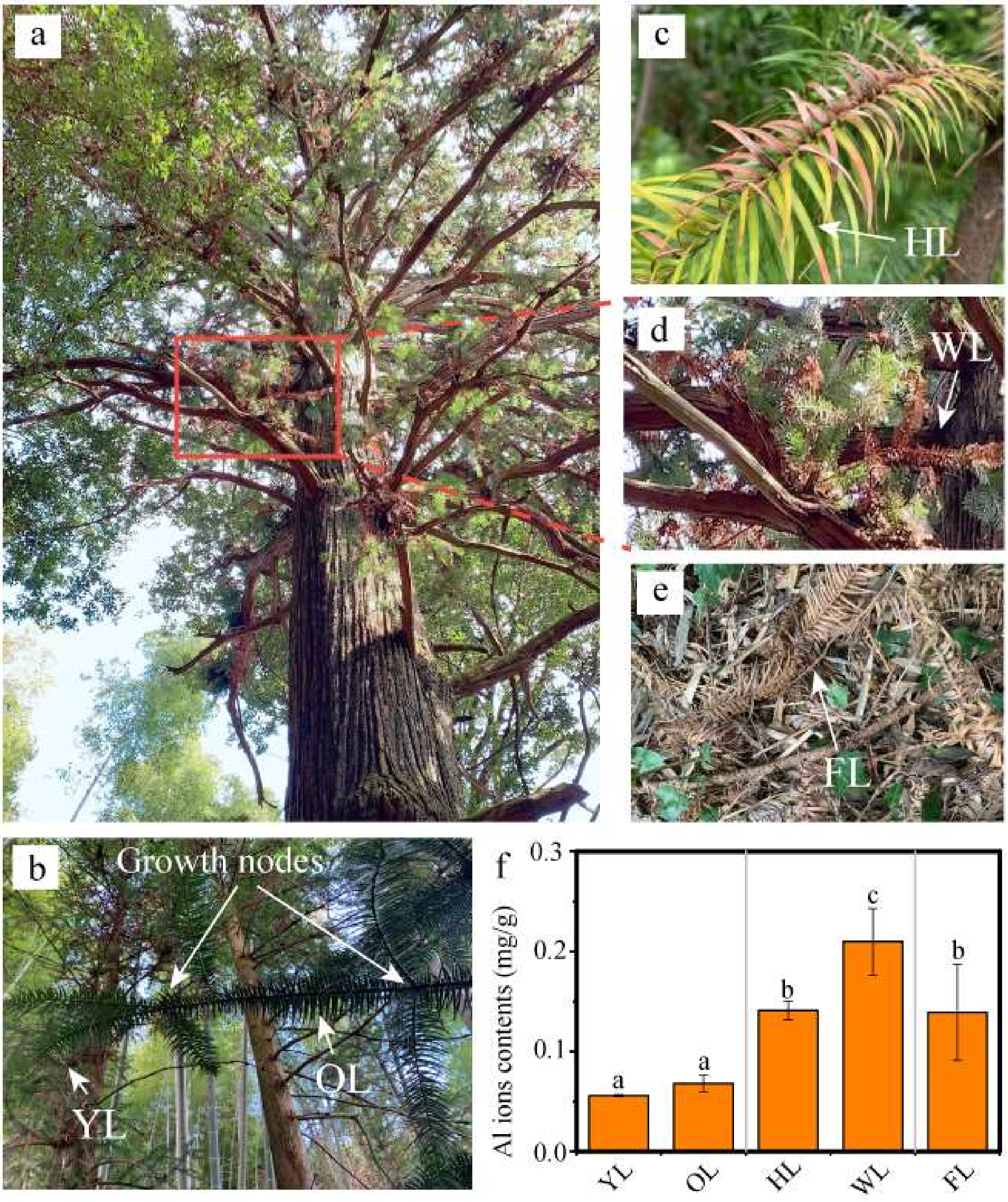

Figure S18. ***C. lanceolata*** **leaves in different growth periods and their Al ions contents.** **(a)** A mature individual of *C. lanceolata*. Living and withered leaves could be observed in this individual. The details of the leaves at different stages: **(b)** The young leaves (YL) and the old leaves (OL). **(c)** The half-withered leaves (HL). **(d)** Persistent withered leaves (WL). **(e)**The fallen withered leaves (FL). Withered leaves fall off with the branches only when self-pruning occurs. **(f)** The aluminum ion content in different growth stages of leaves. There is no significant difference at p = 0.05 level between groups labeled with the same letter.

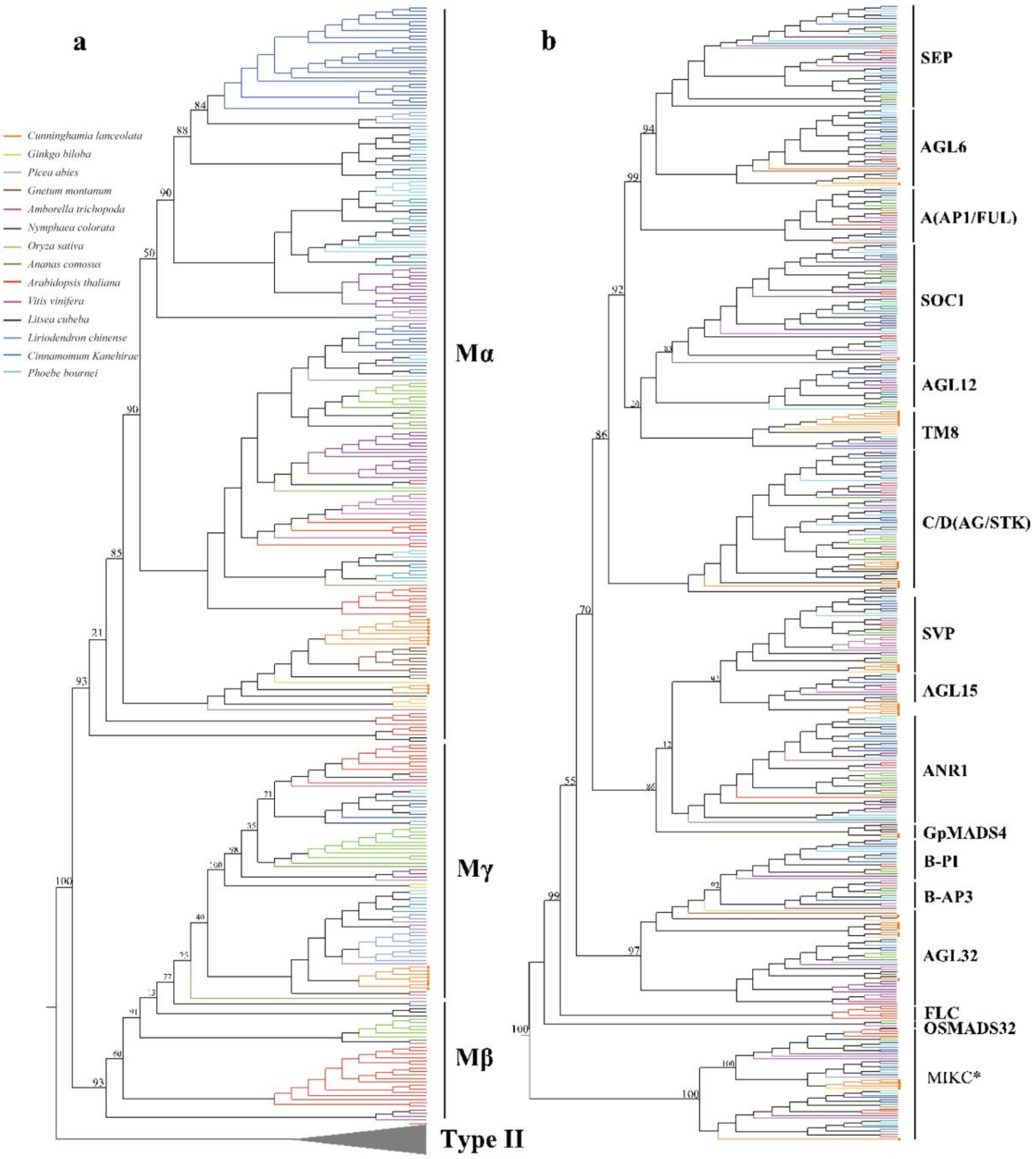

Figure S19. Phylogenetic tree of MADS-box genes from gymnosperms and angiosperms. **(a)** Phylogenetic tree of MADS-box Type I genes. **(b)** Phylogenetic tree of MADS-box Type II genes.

Table S1. The statistics of the quality on the PacBio sequencing data of *C. lanceolata* genome.

Reads type	Reads num	Total bases (bp)	Reads N50 (bp)	Mean length (bp)	Longest read (bp)
Subreads	89,507,038	1,113,159,857,423	20,648	12,437	129,985
ZMWreads	66,589,949	959,970,038,241	23,042	14,416	129,985

Reads Type: Subreads is the data set produced by Subreads, and ZMWreads is the longest subreads data set in the ZMW hole.

Table S2. The statistics of the Illumina sequencing data volume, sequencing depth and quality inspection of each library of *C. lanceolata* genome.

Library	Data (Gb)	Depth (X)	Q20 (%)	Q30 (%)
270_1 bp	67.40	6.47	94.87	85.19
270_2 bp	61.53	5.91	94.23	86.42
270_3 bp	65.17	6.25	94.67	87.25
270_4 bp	58.48	5.61	94.94	87.86
270_5 bp	63.25	6.07	94.34	86.70
270_6 bp	66.94	6.42	94.42	86.88
270_7 bp	70.19	6.74	94.50	87.06
270_8 bp	63.46	6.09	94.31	86.47
Total	516.42	49.56	--	--

Library: the sequencing library of the survey graph; Data (Gb): the amount of sequencing data of the corresponding sequencing library; Depth (X): the depth of sequencing; Q20 (%): the percentage of bases with a sequencing quality value of 20 or more; Q30 (%): The proportion of bases whose sequencing quality value is above 30.

Table S3. Assembly statistics of the *C. lanceolata* genome.

Type	Number/length/percentage
Contig number	28,364
Contig length (bp)	11,242,038,337
Contig N50 (bp)	2,155,103
Contig N90 (bp)	232,277
Contig max (bp)	22,388,200
GC content (%)	36.95

Table S4. The length of chromosome by Hi-C assemble of the *C. lanceolata* genome.

Chromosome	Cluster Num	Cluster Len (bp)	Order Num	Order Len (bp)
Chr01	3,692	1,550,680,665	3,251	1,488,319,881
Chr02	2,701	1,263,832,510	2,267	1,184,309,377
Chr03	2,640	1,234,995,222	2,238	1,182,746,149
Chr04	2,760	1,178,736,379	2,063	1,047,693,580
Chr05	1,913	960,425,685	1,611	927,725,105
Chr06	1,901	931,596,408	1,552	862,932,688
Chr07	1,804	897,221,591	1,527	863,305,216
Chr08	1,365	778,813,723	1,139	747,012,144
Chr09	1,232	749,408,730	988	712,708,477
Chr10	2,070	710,732,703	1,914	694,515,374
Chr11	1,682	637,087,857	1,491	616,297,228
Total<br>(Ratio %)	23,760<br>(83.03)	10,893,531,473<br>(96.9)	20,041<br>(84.35)	10,327,565,219<br>(94.8)

Cluster Len (bp): The length of the sequence located on the chromosome. Order Len (bp): In the sequence located on the chromosome, the length of the sequence can be determined in order and direction.

Table S5. The statistic result of Hi-C assembled of the genome of *C. lanceolata*.

Type	Contig	Scaffold
	**Size (bp)**	**Size (bp)**
N90	227,972	616,446,228
N50	2,097,655	927,886,105
Longest	21,974,839	1,488,644,881
Total length	11,242,038,337	11,244.041,337

Table S6. Illumina sequence alignment statistics of the genome of *C. lanceolata*.

Type	Number/percentage
Total reads	1,882,235,817
Mapped reads	1,864,713,766
Mapped (%)	99.07
Properly mapped reads	1,737,960,488
Properly mapped (%)	92.83

Total reads: reads statistics of filtered data; Mapped Reads: Reads statistics of matched genomes; Properly mapped reads: Reads statistics of mapped genomes and paired.

Table S7. The prediction of gene numbers of the *C. lanceolata* genome.

Method	Software	Species	Gene number
Ab initio	Genscan		68,124
	GlimmerHMM		190,423
	GeneID		179,448
	SNAP		118,942
Homology-based	GeMoMa	*Arabidopsis thaliana*	25,405
		Ginkgo biloba	38,598
		Gnetum montanum	29,888
		Picea abies	32,849
		Populus trichocarpa	30,476
		Pinus taeda	53,107
RNAseq	TransDecoder		115,300
	GeneMarkS-T		58,760
	PASA		55,025
Integration	EVM		37,225

Table S8. The statistics results of function annotation in genome of *C. lanceolata*.

Annotation database	Annotated number	Percentage (%)
GO Annotation	18,857	50.66
KEGG Annotation	12,874	34.58
KOG Annotation	20,771	55.8
Pfam Annotation	29,051	78.04
Swissprot Annotation	26,239	70.49
TrEMBL Annotation	34,202	91.88
Nr Annotation	34,392	92.39
All Annotation	34,559	92.84

Table S9. Statistics on the annotation of non-coding RNA of the *C. lanceolata* genome.

RNA classification	Number	Family
miRNA	50	13
rRNA	2,930	4
tRNA	3,955	24
snRNA	284	8
snoRNA	208	2

Table S10. Statistic result of clustered gene families of 19 species.

Species	Genes	Unclustered genes	Clustered genes	Familys	Unique families	Unique families genes	Common families	Common families genes	Average genes per family
A. alba	50,466	4,996	45,470	11,628	2,556	22,384	876	3,119	3.91
A. angustus	14,629	1,358	13,271	8,461	428	1,953	876	1,529	1.568
A. filiculoides	20,203	2,016	18,187	9,615	473	1,478	876	3,015	1.892
A. thaliana	27,416	2,244	25,172	10,084	983	4,422	876	4,523	2.496
A. trichopoda	26,846	4,326	22,520	10,929	986	4,546	876	3,075	2.061
W. mirabilis	39,019	4,877	34,142	10,458	1,519	10,647	876	3,921	3.265
C. kanehirae	26,531	1,273	25,258	10,012	571	2,551	876	4,660	2.523
C. panzhihuaensis	32,353	4,114	28,239	11,851	1,083	4,940	876	4,182	2.383
G. biloba	41,309	8,606	32,703	12,991	1,646	7,400	876	4,008	2.517
G. montanum	27,491	2,223	25,268	10,999	984	4,245	876	3,464	2.297
P. taeda	36,732	5,668	31,064	6,380	1,995	12,092	876	4,051	4.869
N. tetragona	31,589	3,930	27,659	10,834	1,100	6,484	876	3,500	2.553
O. sativa	27,694	4,856	22,838	9,883	1,188	4,445	876	3,986	2.311
P. abies	26,437	2,273	24,164	9,880	545	1,788	876	4,466	2.446
P. patens	20,328	1,273	19,055	9,155	866	2,455	876	3,097	2.081
S. cucullata	19,779	2,821	16,958	9,634	387	1,205	876	2,641	1.76
S. moellendorffii	22,285	1,770	20,515	9,721	1,550	6,100	876	2,594	2.11
C. lanceolata	37,225	2,605	34,620	13,266	797	2,982	876	5,337	2.61
T. wallichiana	44,035	2,882	41,153	14,364	1,761	7,231	876	4,959	2.865

Table S16. Primary genome assembly statistics of two assembly version.

	Assembly in this paper	Shirasawa's assembly
Sequencing / assembly method	PacBio 20-Kb single-molecule real-time sequencing; Illumina paired-end sequencing; Hi-C sequencing	Long-insert libraries sequencing (PacBio HiFi sequencing); Short-insert libraries sequencing
Assembly level	Chromosome	Contig
No. of Chromosome	11	--
GC content (%)	36.95	36.8
No. of contigs	28,364	2,472
Total contig size (bp)	11,242,038,337	11,548,046,079
Contig N50 (bp)	2,155,103	11,743,989
Assembled genome BUSCO score (%)	93.64	89.10
No. of protein-coding genes	37,225	--
Assembled protein BUSCO score (%)	89.60	--

Table S17. The coverage of the assembly map by Shirasawa *et al.* on chromosomal assembly in this paper.

	Chromosome length (Lin et al.)	Mapped length (Shirasawa et al.)	Coverage (%)
Chr 01	1,488,644,881	1,404,697,105	94.36
Chr 02	1,184,535,977	1,106,996,180	93.45
Chr 03	1,182,969,849	1,120,323,086	94.70
Chr 04	1,047,899,780	962,253,861	91.83
Chr 05	927,886,105	880,128,580	94.85
Chr 06	863,087,788	803,707,019	93.12
Chr 07	863,457,816	815,136,686	94.40
Chr 08	747,125,944	704,957,323	94.36
Chr 09	712,807,177	679,238,847	95.29
Chr 10	694,706,674	656,190,478	94.46
Chr 11	616,446,228	586,853,238	95.20

Table S18. Statistics of *C. lanceolata* gene structure information.

Gene type	Number/length
Gene number	37,225
Gene length (bp)	1,112,864,908
Average gene length (bp)	29,895.63
Exon number	164,956
Exon length (bp)	43,643,130
Average exon length (bp)	264.57
Intron number	164,955
Intron length (bp)	1,069,221,778
Average intron length (bp)	6,482

Table S19. Statistic of different types of repeat sequence in *C. lanceolata* genome.

Type	Number	Length	Rate (%)
ClassI	13,217,000	9,755,331,388	86.78
ClassI/DIRS	954,220	928,392,143	8.26
ClassI/LINE	595,272	365,970,956	3.26
ClassI/LTR	485,367	411,052,389	3.66
ClassI/LTR/Copia	3,107,217	2,684,662,505	23.88
ClassI/LTR/Gypsy	4,233,916	4,764,617,838	42.38
ClassI/LTR|DIRS	140	65,857	0
ClassI/PLE|LARD	3,797,243	2,243,877,805	19.96
ClassI/SINE	3,706	805,063	0.01
ClassI/SINE|TRIM	832	875,820	0.01
ClassI/TRIM	33,556	21,340,727	0.19
ClassI/Unknown	5,531	4,488,270	0.04
ClassII	514,745	382,500,579	3.4
ClassII/Crypton	145	183,686	0
ClassII/Helitron	70,297	34,790,020	0.31
ClassII/MITE	4,567	2,757,596	0.02
ClassII/Maverick	19,158	11,853,833	0.11
ClassII/TIR	368,383	249,279,504	2.22
ClassII/Unknown	52,195	84,861,181	0.75
PotentialHostGene	183,062	118,181,144	1.05
SSR	26,634	30,066,332	0.27
Unknown	1,401,192	700,661,880	6.23
Total	13,941,441	10,377,888,279	92.31

Table S20. Statistic of LAI in each chromosomes.

Chr	LAI
Chr01	19.54
Chr02	20.00
Chr03	20.00
Chr04	20.09
Chr05	17.20
Chr06	16.94
Chr07	17.69
Chr08	18.00
Chr09	17.88
Chr10	17.82
Chr11	19.13

Table S21. Hypothetical WGDs, posterior mean of duplicate retention rate (q), and the Bayes Factor (K) to compare the likelihood of q = 0 (H0) to the likelihood of q > 0 (H1) using the Savage-Dickey density ratio.

Hypotheses	Relaxed branch-specific model		Critical branch-specific model	
	$\bar{q}$	K	$\bar{q}$	K
WGD1	0.11233	0.25962*	0.23817	0.06824**
WGD2	0.37868	0.04426**	0.17672	0.09463**
WGD3	0.08559	0.72805	0.00028	2899.97850
WGD4	0.03037	3.58108	0.00037	3564.65813
WGD5	0.59532	0.02821***	0.00332	246.95914

$K < 1/102$ or $K < 0.01$, decisive evidence against H_0****; $K < 1/101.5$ or $K < 0.0316$, very strong evidence against H_0***; $K < 1/10$ or K < 0.1, substantial evidence against H_0**; $K < 1/100.5$ or $K < 0.\ 3162$ substantial evidences against H_0*; K < 1, H_1 supported, not worth more than a bare mention; $K > 1$, H_0 supported.

Table S22 Details of the samples collection for evolutionary history analysis of *C. lanceolata*.

Group	Location	Amount	Latitude	Longitude	Altitude (m)
**GZH**	Huishui County, Guizhou province, China	10	25°15′~25°35′N	106°45′~106°57′E	900
**HNX**	Xinning County, Shaoyang City, Hunan Province, China	13	26°15′~26°55′N	110°18′~110°28′E	1300
**SXA**	Xinhua village, Ankang City, Shaanxi province, China	13	31°42′~31°59′N	108°07′~110°11′E	850
**FJM**	Meihuashan Nature Reserve, Longyan City, Fujian Province, China	12	25°15′~25°35′N	116°45′~116°57′E	1800
**SCD**	Dechang County, Sichuan Province, China	13	27°06′~27°35′N	101°12′~102°24′E	1050
**JSZ**	Xiashu County, Zhenjiang City, Jiangsu Province, China	12	32°04′~32°12′N	119°04′~119°15′E	260
**JXJ**	Jiulianshan Nature Reserve, Ganzhou City, Jiangxi Province, China	11	24°30′~24°52′N	114°33′~114°33′E	1200
**JXW**	Wengongshan Forest Park, Wuyuan City, Jiangxi Province, China	20	29°10′~29°11′N	117°46′~117°47′E	200
**TWB**	Baxianshan Nature Reserve, Taichung County, Taiwan Province, China	11	24°07′~24°09′N	120°15′~121°27′E	2000
**YNN**	Na Ri, Bac Kan province, Vietnam	13	23°07′~23°11′N	105°40′~105°44′E	1100

Table S25. List of putative flavonoid synthesis related genes in C. lanceolata genome.

Abbreviation	Gene name	Gene ID
PAL	Phenylalanone ammonia-lyase	Cl16939; Cl23281; Cl20012; Cl15629; Cl01003; Cl10224
C4H	Cinnamate 4-hydroxylase	Cl32060; Cl22669; Cl27969
4CL	Coumarate-4-CoA ligase	Cl32500; Cl18950; Cl23923; Cl29221; Cl20157; Cl11300; Cl08738; Cl36046; Cl18336; Cl23029; Cl33619; Cl25933; Cl21436; Cl09422; Cl29190; Cl26264; Cl16358; Cl02585; Cl23628; Cl07412; Cl15006; Cl27201; Cl16061; Cl01710; Cl33345; Cl22156; Cl13819; Cl22391; Cl33941; Cl36440; Cl15332; Cl34086; Cl04819; Cl06575; Cl24383
CHS	Chalcone synthase	Cl20463; Cl16987; Cl13810; Cl18516; Cl13939; Cl35852; Cl36558; Cl25812; Cl36434; Cl23043; Cl12414; Cl02164
CHI	chalcone-flavanone isomerase	Cl23637; Cl26105; Cl35530; Cl01348
F3H	flavanone-3-hydroxylase	Cl05230; Cl22835; Cl19511; Cl30186; Cl27251; Cl05548; Cl23387; Cl09496
F3'H	flavonoid 3'-hydroxylase	Cl26386; Cl20734; Cl00578
F3'5'H	flavonoid 3',5'-hydroxylase	Cl11848; Cl12701; Cl03636; Cl28266; Cl06640; Cl09151; Cl01764; Cl12036
FLS	Flavonol synthase	Cl13876; Cl03153; Cl27668; Cl21034; Cl01929; Cl15880; Cl05506; Cl33239; Cl21939; Cl14727; Cl09073; Cl34583
DFR	dihydroflavonols 4-reductase	Cl01163; Cl14835; Cl32921; Cl34319; Cl17992; Cl09745; Cl19190; Cl10044; Cl25990; Cl21164; Cl20763; Cl34597; Cl16924; Cl30627; Cl10196
LAR	leucoanthocyanidins reductase	Cl19924; Cl17849; Cl02972; Cl22572; Cl04043; Cl32845; Cl08749; Cl03800; Cl01405; Cl26343
ANS	anthocyanidin synthase	Cl28499
ANR	anthocyanidin reductase	Cl02683; Cl08429; Cl27585; Cl01531; Cl00030; Cl14400; Cl29306; Cl29276; Cl27034; Cl27658; Cl25902; Cl03085; Cl09863; Cl21775; Cl06423; Cl10344; Cl18261; Cl10859; Cl23026; Cl30270; Cl14318; Cl20814; Cl15108; Cl26250; Cl09989

Table S27. Blast results of 4 astringent seed related genes in *C. lanceolata* in the Shirasawa's assembly.

Gid in Shirasawa's assembly	Gid in assembly in this paper	Identity	Query coverage	Subject coverage
Clan1275G00001.1	Cl19924.1	99.76	99.92	99.92
Clan685G00029.1	Cl30227.1	99.41	99.85	90.67
Clan997G00018.1	Cl02910.1	99.05	99.88	99.88
-	Cl08429.1	-	-	-

Table S28. List of MADS-box genes identified in *C. lanceolata* genome.

Gene ID	Accession number	Location	Chr	ORF (bp)	Size (aa)	Group
Cl10262	MT103468	519587430-519650702	1	678	225	AG
Cl36126	MT103469	526300958-526365655	1	678	225	AG
Cl26543	MT103470	521807977-521978150	1	756	251	AG
Cl26063	MT103471	70041826-70094329	11	693	230	AG
Cl13850	MT103472	70233297-70328225	11	471	156	AG
Cl35439	MT103473	89314620-89335958	2	666	221	AG
Cl35065	MT103474	94642941-94733811	2	738	245	AGL6
Cl29520	MT103475	366538841-366706949	11	471	156	AGL6
Cl35039	MT103477	1392696700-1393060149	1	927	308	TM8
Cl22970	MT103478	1393706469-1394059060	1	636	211	TM8
Cl12097	MT103479	1392261362-1392431943	1	636	211	TM8
Cl11956	MT103480	201000156-201216026	1	636	211	TM8
Cl30647	MT103481	1395412787-1395891794	1	636	211	TM8
Cl17451	MT103482	426919818-427049392	10	465	154	TM8
Cl36264	MT103483	98633241-98845766	2	696	231	TM3
Cl34784	MT103484	596818838-596974165	6	708	235	SVP
Cl35571	MT103485	745221257-745358270	3	651	216	SVP
Cl01446	MT103486	746814647-747037347	3	720	239	SVP
Cl22549	MT103492	505217517-505359167	11	681	226	GpMADS4
Cl29629	MT103493	502196965-502224894	11	612	203	GpMADS4
Cl27678	MT103494	291146007-291466416	8	813	270	GGM13
Cl28889	MT103495	758894530-758896706	2	846	281	DEF/GLO
Cl22633	MT103496	750408487-750411952	2	588	195	DEF/GLO
Cl08169	MT103497	750587944-750592183	2	621	206	DEF/GLO
Cl27804	MT103498	759848871-759850338	2	525	174	DEF/GLO
Cl17023	MT103499	759890589-759892578	2	726	241	DEF/GLO
Cl14691	MT103500	746553907-746558043	2	642	213	DEF/GLO
Cl31654	MT103501	568065106-568070272	2	1062	353	MIKC*
Cl08187	MT103502	685865937-685938418	9	1152	383	MIKC*
Cl07566	MT103503	684871634-684879797	9	1131	376	MIKC*

Cl22501	MT103504	567007448-567046037	11	1131	376	MIKC*
Cl31662	MT103505	687228890-687234262	9	1146	381	MIKC*
Cl15999	MT103506	621864422-621864784	4	363	120	Mα
Cl24955	MT103507	716567113-716567590	4	318	105	Mα
Cl21596	MT103508	4079400-4079645	6	246	81	Mα
Cl00442	MT103509	143546-144199	/	654	217	Mα
Cl08495	MT103510	718529476-718530006	4	531	176	Mα
Cl21016	MT103511	695835786-695836619	1	834	277	Mα
Cl09032	MT103512	575513819-575514562	3	744	247	Mα
Cl29136	MT103513	574354493-574355266	3	774	257	Mα
Cl07839	MT103514	835739771-835740823	3	1053	350	Mα
Cl07629	MT103515	6402122-6403189	8	1068	355	Mα
Cl30941	MT103516	492704690-492705757	10	1068	355	Mα
Cl01366	MT103517	561794249-561795499	3	1251	416	Mγ
Cl19059	MT103518	979636832-979638091	3	1260	419	Mγ
Cl05223	MT103519	494123266-494124511	5	1182	393	Mγ
Cl18167	MT103520	979988133-979989272	3	1140	379	Mγ
Cl14558	MT103521	531037074-531037772	7	699	232	Mγ
Cl34141	MT103522	770338139-770339431	7	1293	430	Mγ
Cl00625	MT103523	980578572-980579186	3	615	204	Mγ

Table S29. The sequencing quality of the raw data from the nine Hi-C sequencing libraries.

Library	ReadSum	BaseSum	GC (%)	N (%)	Q20 (%)	Q30 (%)
L01	231,458,781	69,320,910,642	38.33	0	97.01	93.09
L02	216,574,783	64,867,446,176	38.26	0.03	94.86	88.84
L03	245,663,762	73,581,674,890	38.2	0.03	95.09	89.24
L04	214,400,226	64,223,373,938	38.23	0.01	95.81	90.6
L05	196,182,529	58,763,528,824	38.33	0.01	95.71	90.43
L06	248,263,233	74,357,707,184	38.34	0	97.05	93.21
L07	274,734,867	82,259,170,272	38.17	0	97	95.29
L08	251,334,974	75,261,718,710	38.17	0	96.16	94.07
L09	254,106,397	76,093,916,328	38.15	0	96.76	94.94
Total	2,132,719,552	638,729,446,964	/	/	/	/

Table S30. The statistics of the mapped efficiency of the pairs on the nine Hi-C sequencing library data.

Library	Type	Number	Ratio (%)
L01	Unique Paired Alignments	74,149,083	100
	Valid Interaction Pairs	68,649,601	92.58
	Dangling End Pairs	3,640,918	4.91
	Re-ligation Pairs	405,084	0.55
	Self-cycle Pairs	99,755	0.13
	Dumped Pairs	1,353,725	1.83
L02	Unique Paired Alignments	67,062,534	100
	Valid Interaction Pairs	60,859,580	90.75
	Dangling End Pairs	4,395,499	6.55
	Re-ligation Pairs	415,483	0.62
	Self-cycle Pairs	110,980	0.17
	Dumped Pairs	1,280,992	1.91
L03	Unique Paired Alignments	75,942,872	100
	Valid Interaction Pairs	68,950,679	90.79
	Dangling End Pairs	4,966,975	6.54
	Re-ligation Pairs	468,638	0.62
	Self-cycle Pairs	124,476	0.16
	Dumped Pairs	1,432,104	1.89
L04	Unique Paired Alignments	68,132,268	100
	Valid Interaction Pairs	62,320,281	91.47
	Dangling End Pairs	3,960,157	5.81
	Re-ligation Pairs	403	0.59
	Self-cycle Pairs	116,034	0.17
	Dumped Pairs	1,333,095	1.96
L05	Unique Paired Alignments	60,413,157	100
	Valid Interaction Pairs	54,939,692	90.94
	Dangling End Pairs	3,799,713	6.29
	Re-ligation Pairs	371,197	0.61
	Self-cycle Pairs	92,730	0.15
	Dumped Pairs	1,209,825	2.00
L06	Unique Paired Alignments	80,471,301	100
	Valid Interaction Pairs	74,585,391	92.69
	Dangling End Pairs	3,831,391	4.76
	Re-ligation Pairs	480,089	0.6
	Self-cycle Pairs	87,930	0.11
	Dumped Pairs	1,486,500	1.85
L07	Unique Paired Alignments	89,802,324	100
	Valid Interaction Pairs	82,996,299	92.42
	Dangling End Pairs	4,306,629	4.8

	Re-ligation Pairs	475,349	0.53
	Self-cycle Pairs	146,436	0.16
	Dumped Pairs	1,877,611	2.09
L08	Unique Paired Alignments	82,128,266	100
	Valid Interaction Pairs	76,225,099	92.81
	Dangling End Pairs	3,705,017	4.51
	Re-ligation Pairs	481,714	0.59
	Self-cycle Pairs	86,016	0.1
	Dumped Pairs	1,630,420	1.99
L09	Unique Paired Alignments	84,783,347	100
	Valid Interaction Pairs	79,165,448	93.37
	Dangling End Pairs	3,374,676	3.98
	Re-ligation Pairs	474,558	0.56
	Self-cycle Pairs	76,547	0.09
	Dumped Pairs	1,692,118	2.0

Unique Paired Alignments: The only read pairs aligned to the assembled genome; Valid Interaction Pairs: Valid Read Pairs; Dangling End Pairs: Data of end suspension type in invalid read pairs; Re-ligation Pairs: Dara of adjacent connection type in invalid read pairs; Self-circle Ligation Pairs: The invalid data belongs to read pairs of self-connecting type; Dumped Pairs: The invalid data belongs to other undefined read pairs.

Table S31. Primer designs with *Spe I* restriction sites for the candidate genes infusion cloning.

Primer Name	Primer sequence
ANR3301-F	5’TGACCTCGAGACTAGTATGAGTTGCACTAAGAAGGT3’
ANR3301-R	5’TGTAGTCCATACTAGTGTTACCGACATCATTAGAGC3’
LAR3301-F	5’TGACCTCGAGACTAGTATGGCCTGTGCTCCCAAAGT3’
LAR3301-R	5’TGTAGTCCATACTAGTAAGGTACTGGTTGAAAAAAT3’
DET23301-F	5’TGACCTCGAGACTAGTATGGCGCATGTTCTGCAACA3’
DET23301-R	5’TGTAGTCCATACTAGTGAATATGAAGGGGAAAAGAG3’
BZR13301-F	5’TGACCTCGAGACTAGTATGTCCACCCGCTCCGTAAT3’
BZR13301-R	5’TGTAGTCCATACTAGTGGGACAAAAATGTTGAGACA3’

Method S1. Observation and determination of the structure of astringent and germinating seeds in different development stages.

Samples were collected from the Youxi National Forest Farm, Fujian Province, China. The geographical coordinates of the orchard are 25°50 ' – 26°26' N, 117°48 ' – 118°39' E. In 2017, ten healthy individuals with similar height, diameter, and annual astringent seed incidence rates were randomly selected from the same orchard area. The date of artificial supplementary pollination (1st March) was recorded as day 0. After 60 days, three cones from each individual were collected every 5 days until the seeds were harvested. We observed the structures of the astringent and germinating seeds at 95, 105, 115, 125, 135, 145, 155, 165, 175, and 185 d using an optical microscope. During observation, the seed coats were quickly and carefully removed and the embryos were examined under 20× magnification using an optical microscope. Based on their colour, lustre, and hardness, embryos at different growth stages were judged to be astringent or germinating seeds.

Method S2. Metabolome detection at different stages of astringent seeds and germinating seeds.

2.1 Sample collection, processing, and storage

The samples used for transcriptomic analysis were also used for the metabolomic assays. The sampling collection, processing, and storage are described in the 'Transcriptomic data and analysis' in the 'Online methods' section. Please refer to this section for details regarding sampling methods.

2.2 Sample preparation and extraction

All freeze-dried samples were crushed using a mixer mill (MM 400, Retsch) with zirconia beads for 1.5 min at 30 Hz. One hundred milligrams of powder of each sample was weighed and extracted overnight at 4°C with 1.0 mL 70% aqueous methanol. Following centrifugation at 10000 × *g* for 10 min, the extracts were absorbed (CNWBOND Carbon-GCB SPE Cartridge, 250 mg, 3 mL; ANPEL, Shanghai, China, www.anpel.com.cn/cnw) and filtered (SCAA-104, 0.22 μm pore size; ANPEL, Shanghai, China, http://www.anpel.com.cn/) before LC-MS analysis.

2.3 HPLC and ESI-Q TRAP-MS/MS conditions

The sample extracts were analysed using an LC-ESI-MS/MS system (Shim-pack UFLC SHIMADZU CBM30A system, www.shimadzu.com.cn/; MS, Applied Biosystems 4500 Q TRAP, www.appliedbiosystems.com.cn/). The analytical conditions were as follows, HPLC: column, Waters ACQUITY UPLC HSS T3 C18 (1.8 μm, 2.1 mm × 100 mm); solvent system, water (0.04% acetic acid): acetonitrile (0.04% acetic acid); gradient program, 95:5 V/V at 0 min, 5:95 V/V at 11.0 min, 5:95 V/V at 12.0 min, 95:5 V/V at 12.1 min, 95:5 V/V at 15.0 min; flow rate, 0.40 mL/min; temperature, 40°C; injection volume: 5 μL. The effluent was alternately connected to an ESI-triple quadrupole linear ion trap-MS (QTRAP)-MS.

LIT and triple quadrupole (QQQ) scans were acquired on a triple quadrupole-linear ion trap mass spectrometer (Q TRAP), API 4500 Q TRAP LC/MS/MS System, equipped with an ESI Turbo Ion-Spray interface, operating in positive ion mode, and controlled by the Analyst 1.6 software (AB Sciex). The ESI source operation parameters were as follows: an ion source, turbo spray; source temperature, 550°C; ion-spray voltage (IS), 5500 V; ion source gas I (GSI), gas II(GSII), and curtain gas (CUR) were set at 55, 60, and 25.0 psi, respectively; the collision gas (CAD) was high. Instrument tuning and mass calibration were performed with 10 and 100 μmol/L polypropylene glycol solutions in QQQ and LIT modes, respectively. QQQ scans were acquired as multiple reaction monitoring (MRM)

experiments with the collision gas (nitrogen) set at 5 psi. The DP and CE for individual MRM transitions were further DP and CE optimization.

2.4 Qualitative and quantitative analyses of metabolites

Based on the self-built database and public databases (HMDB, http://www.hmdb.ca/; METLIN, https://metlin.scripps.edu/; and KEGG, http://www.kegg.jp/kegg/compound/), qualitative analysis was carried out according to the second spectral information. The isotope signals, repeated signals containing K^+, Na^+, and $NH4^+$ ions, and repeated signals of fragment ions with other larger-molecular-weight substances were removed.

Quantification was performed using the MRM model with a triple four-stage rod mass spectrometer. After obtaining the metabolite spectrum analysis data of different samples, the peak integral area was calculated for all mass spectrum peaks and integral correction was performed for the spectrum peaks of the same metabolite in different samples.

2.5 Identification and verification of different expressive metabolites

Analyst v1.6.1 software was used to process raw mass spectrometry data. The MultiaQuant software was used to integrate and correct the chromatographic peaks. The area of each chromatographic peak represented the relative content of the corresponding compounds. Finally, chromatographic peak integral area data were derived. Principal component analysis, orthogonal partial least squares discriminant analysis (OPLS-DA), and Pearson correlation coefficient calculations were performed using RStudio software (www.r-project.org/).

Metabolites from different groups were preliminarily screened based on variable importance in the project (VIP) value obtained from the OPLS-DA model. The fold-change values were combined to screen differentially expressed metabolites (DEMs). The metabolites with FC of ≥ 2.0 or FC of ≤ 0.5 were considered to differ more than twice or less than 0.5 between the Astringent Seed (AS) and Germinate Seed (GS) groups, while a VIP value of ≥ 1.0 was indicative of a significant effect on the classification of samples in each group in the OPLS-DA model. Metabolites that fit both conditions were selected as DEMs.

To verify the accuracy of the relative quantification results generated from LC-MS signals, 16 metabolites were randomly selected for further HPLC quantification. The HPLC conditions were the same as those described in Section 2.3. Standards for the selected metabolites were obtained from the Shinemro Chemical Platform (http://www.shinemro.com).

Method S3. Transgenic verification in *A. thaliana*.

We employed the Agrobacterium-mediated transformation method to introduce candidate genes associated with astringent seed formation regulation into *Arabidopsis thaliana*. These included *ClLAR* and *ClANR* from the flavonoid biosynthesis pathway, and *ClDET2* and *ClBZR1* from the brassinosteroid signaling pathway. Phenotypic changes following overexpression of these genes were then observed. The procedure is outlined below:

Primers flanking the *Spe I* restriction sites in the CDS region of the candidate genes were designed based on genomic data (**Table S31**). After PCR amplification, the products were cloned using the Infusion cloning method and sequenced for alignment. Amplification was then carried out using Tks Gflex DNA Polymerase. The desired PCR bands were purified with the MiniBEST Agarose Gel DNA Extraction Kit V.4.0. Plasmid pCambia3301 was digested with Spe I, and the vector DNA was purified. In-Fusion reactions were conducted following the In-Fusion HD Cloning Kit protocol, with the following components: 2.0 μl of vector DNA (50 ng/μl), 1.0 μl of PCR product (50 ng/μl), 2.0 μl of 5×

In-Fusion HD Enzyme Premix, and 5.0 μl of dH2O. The reaction was incubated at 50°C for 15 minutes. A 2.5 μL aliquot of the reaction mixture was heat-transformed into E. coli Competent Cells JM109 and incubated overnight at 37°C. After positive colonies appeared, the bacteria were cultured, and the recombinant plasmid was extracted using the MiniBEST Plasmid Purification Kit.

Agrobacterium transformation was performed using the freeze-thaw method. The GV3101 strain was cultured in autoclaved YEB medium until the OD600 reached 0.8-1.0, indicating activation. Recombinant plasmid (800 ng) was mixed with 100 μl of activated competent cells, vortexed briefly, and incubated on ice for 30 minutes. The mixture was then frozen in liquid nitrogen for 1 minute, followed by a 5-minute heat shock at 37°C and a 3-minute incubation on ice. This procedure was repeated once. Next, 400 μl of antibiotic-free YEB medium was added, and the mixture was incubated in the dark at 28°C with shaking at 200 rpm for 4 hours. After a 1-minute centrifugation at 10,000 rpm, 100 μl of the supernatant was used to resuspend the Agrobacterium, which was then plated on YEB agar. Once positive colonies appeared, they were cultured in YEB medium until the OD600 reached 0.8-1.0, at which point the culture was ready for infection.

The inflorescence infection method was used to transform *A. thaliana* during its flowering stage. The plants were submerged for 1 minute in an infection solution mixed with L/LSilwet L-77 transformation liquid and then incubated in the dark for 24 hours. After incubation, the plants were cultured until seed maturation, at which point the seeds were harvested, sown, and grown to obtain T1 generation seedlings. Once cotyledons emerged, the seedlings underwent resistance screening with a 1,000-fold dilution of Basta solution. Resistant seedlings were transplanted, and successful gene integration was confirmed through PCR using the TransDirect Plant Tissue PCR Kit. The PCR reaction mixture contained 4.0 μl of lysis buffer, 0.4 μl of each primer, 10 μl of 2 × TransDirect PCR SuperMix, and 5.2 μl of dH_2O.

Then we allowed the T1 generation transgenic *A. thaliana* seedlings to mature and produce seeds. The seeds were then analyzed phenotypically, including evaluations of seed appearance and germination rate, to explore the potential role of the candidate genes in reproductive growth.

Method S4. Determination of aluminum in *C. lanceolata*.

We analysed the aluminum content of Chinese fir leaves at different developmental stages. All materials from ten individuals were randomly selected from a third-generation seed orchard at the Youxi National Forest Farm, Fujian Province, China. Five groups of materials were collected, with 10 replicates per group. The five materials were as follows: young (YL) and old leaves from living branches, leaves that survived for one year (WL1) and five years (WL2) in withered branches, and two months of fallen leaves (WL3) from withered branches.

After 1 h of deactivation of enzymes at 105°C, all the samples were dried at 70°C until a constant weight was obtained. The dry samples were ground, crushed, and then filtered through a 0.5 mm sieve. Samples from each group were accurately weighted (0.2 g respectively, and digested with nitric acid and hydrogen peroxide solution using a microwave digestion system (Multiwave ECO). The digested solution was diluted with pure water to a final volume of 50 mL and cooled to room temperature. Subsequently, the concentration of Al ions in each sample group was determined using an inductively coupled plasma method with a PE Optima 8000 system (PerkinElmer, USA).
